# 1,3,4-Oxadiazole *N*-Mannich Bases: Synthesis, Antimicrobial, and Anti-Proliferative Activities

**DOI:** 10.3390/molecules26082110

**Published:** 2021-04-07

**Authors:** Lamya H. Al-Wahaibi, Ahmed A. B. Mohamed, Samar S. Tawfik, Hanan M. Hassan, Ali A. El-Emam

**Affiliations:** 1 Department of Chemistry, College of Sciences, Princess Nourah Bint Abdulrahman University, Riyadh 11671, Saudi Arabia; lhalwahaibi@pnu.edu.sa; 2 Department of Medicinal Chemistry, Faculty of Pharmacy, Mansoura University, Mansoura 35516, Egypt; ahmed_smt@yahoo.com; 3 Department of Pharmaceutical Organic Chemistry, Faculty of Pharmacy, Mansoura University, Mansoura 35516, Egypt; samarsamir1984@gmail.com; 4 Department of Pharmacology and Biochemistry, Faculty of Pharmacy, Delta University for Science and Technology, International Costal Road, Gamasa City, Mansoura 11152, Egypt; hananhafila@hotmail.com

**Keywords:** 1,3,4-oxadiazoles, *N*-Mannich bases, antimicrobial activity, anti-proliferative activity

## Abstract

The reaction of 5-(3,4-dimethoxyphenyl)-1,3,4-oxadiazole-2(3*H*)-thione **3** with formaldehyde solution and primary aromatic amines or 1-substituted piperazines, in ethanol at room temperature yielded the corresponding *N*-Mannich bases 3-arylaminomethyl-5-(3,4-dimethoxyphenyl)-1,3,4-oxadiazole-2(3*H*)-thiones **4a**–**l** or 3-[(4-substituted piperazin-1-yl)methyl]-5-(3,4-dimethoxyphenyl)-1,3,4-oxadiazole-2(3*H*)-thiones **5a**–**d**, respectively. The in vitro inhibitory activity of compounds **4a**–**l** and **5a**–**d** was assessed against pathogenic Gram-positive, Gram-negative bacteria, and the yeast-like pathogenic fungus *Candida albicans*. The piperazinomethyl derivatives **5c** and **5d** displayed broad-spectrum antibacterial activities the minimal inhibitory concentration (MIC) 0.5–8 μg/mL) and compounds **4j**, **4l**, **5a,** and **5b** showed potent activity against the tested Gram-positive bacteria. In addition, the anti-proliferative activity of the compounds was evaluated against prostate cancer (PC3), human colorectal cancer (HCT-116), human hepatocellular carcinoma (HePG-2), human epithelioid carcinoma (HeLa), and human breast cancer (MCF7) cell lines. The optimum anti-proliferative activity was attained by compounds **4l**, **5a**, **5c,** and **5d**.

## 1. Introduction

The 1,3,4-Oxadiazoles are an important class of heterocyclic compounds with diverse pharmacological properties [1,2,3]. The 1,3,4-Oxadiazole nucleus represents an essential building unit in several drugs including the broad spectrum antibacterial drug furamizole [4], the antiretroviral drug raltegravir [5], the anticancer agent zibotentan [6], the anti-obesity/antidiabetic agent AZD 3988 [7], and the antihypertensive drugs tiodazosin [8], and nesadipil [9].

In addition, there is a growing interest in the chemotherapeutic activities of 2,5-disubstituted-1,3,4-oxadiazoles as antibacterial [10,11,12], antifungal [13,14,15], antitubercular [16,17], and antiviral [18,19,20,21] agents. Moreover, 1,3,4-Oxadiazole-2(3*H*)-thiones, their thioether derivatives and 3-aminomethyl analogues (*N*-Mannich bases) are the most interesting for their anticancer activities [21,22]. The 1,3,4-Oxadiazole derivatives exert their anticancer activities via different mechanisms, such as targeting epidermal growth factor receptors (EGFR) [23], vascular endothelial growth factor receptors (VEGF) [24], focal-adhesion kinase (FAK) [25,26], histone deacetylases (HDAC) [27,28], methionine aminopeptidase (MetAP) [29], NF-κB (nuclear factor κB) [30], poly(ADP-ribose) polymerase (PARP-1) [31], thymidine phosphorylase (TP) [32], telomerase [33], thymidylate synthase (TS) [34], zinc-finger protein 143 (ZNF143) [35], and tubulin polymerase [36].

Furthermore, 1,3,4-oxadiazoles were proved to exhibit potent anti-inflammatory [37,38,39], antioxidant [40], antidiabetic [41,42], and monoamine oxidase (MAO) inhibitory activities [43,44]. Besides, 1,3,4-oxadiazole derivatives are highly attractive compounds in the development of organic light-emitting diodes (OLEDs) [45,46].

Furthermore, 3,4-dimethoxyphenyl moiety represents an essential motif in various chemotherapeutic agents with anticancer [47,48,49,50,51,52], antibacterial [53,54], and antiviral [55] activities.

Motivated by the above-mentioned findings and following an ongoing interest in the pharmacological [56] and structural properties [57,58,59] of 1,3,4-oxadiazole-2(3*H*)-thione *N*-Mannich bases, we report herein the synthesis, characterization, antibacterial, antifungal, and anti-proliferative activities of related series of 5-(3,4-dimethoxyphenyl)-1,3,4-oxadiazole-2(3*H*)-thione *N*-Mannich bases.

## 2. Results and Discussion

### 2.1. Chemical Synthesis

**Scheme 1:**
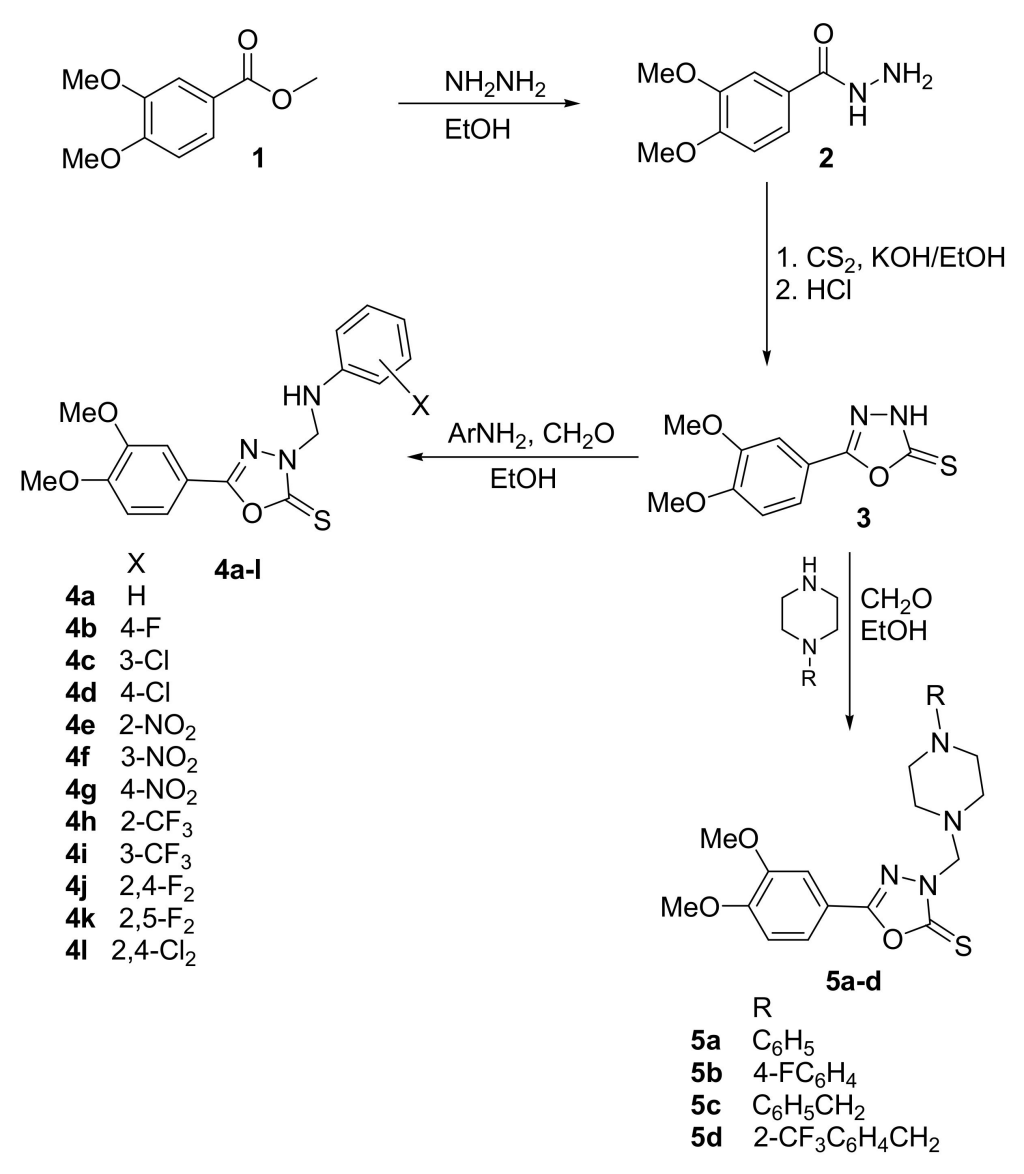
Synthesis of compounds **4a**–**l** and **5a**–**d**.

The 3,4-Dimethoxybenzohydrazide **2** was prepared from the commercially-available methyl 3,4-dimethoxybenzoate **1** via treatment with hydrazine in ethanol [60]. 1,3,4-Oxadiazole-2(3*H*)-thione **3** was obtained via reaction of the carbohydrazide **2** with carbon disulfide in ethanolic potassium hydroxide as previously described [61]. Furthermore, 1,3,4-Oxadiazole-2(3*H*)-thiones were reported to undergo aminomethylation through reaction with primary aromatic amines and formaldehyde to yield the corresponding *N*-Mannich bases [62]. Consequently, treatment of **3** with formaldehyde solution and various primary aromatic amines or 1-substituted piperazines, in ethanol at room temperature yielded their corresponding 3-arylaminomethyl **4a**–**l** or 3-piperazinomethyl **5a**–**d** *N*-Mannich bases, respectively, in good yields (Scheme 1, Table 1).

The structures of compounds **4a**–**l** and **5a**–**d** were confirmed by elemental analyses, ^1^H NMR and ^13^C NMR spectral data.

### 2.2. In Vitro Antibacterial and Antifungal Activities

The in vitro growth inhibitory activity of compounds **4a**–**l** and **5a**–**d** was evaluated towards the standard Gram-positive bacterial strains *Staphylococcus aureus* American type culture collection (ATCC) 6571, *Bacillus subtilis* ATCC 5256 and *Micrococcus luteus* ATCC 27141, Gram-negative bacterial strains *Escherichia coli* ATCC 8726, and *Pseudomonas aeruginosa* ATCC 27853, and the yeast-like pathogenic fungus *Candida albicans* MTCC 227. The initial screening was performed by the semi-quantitative agar-disc diffusion method using Müller-Hinton agar medium [63]. The results of the preliminary screening of compounds **4a**–**l** and **5a**–**d** (200 μg/disc); the antibacterial antibiotics Gentamicin sulfate, Ampicillin trihydrate, and the antifungal drug Clotrimazole (100 μg/disc); and the calculated log *P* values (Clog *P*) are depicted in Table 2.

The results revealed that potent antibacterial activity was displayed by the compounds **4j**, **4l**, **5a**, **5b**, **5c,** and **5d,** which displayed growth inhibition zones ≥ 18 mm against one or more of the tested microorganisms. In addition, compounds **4a**, **4b**, **4c**, **4d**, **4h**, **4i,** and **4k** showed medium activity (growth inhibition zones 14–17 mm) and compounds **4e**, **4f,** and **4g** showed poor antibacterial activity (growth inhibition zones 10–13 mm) against the tested microorganisms. In general, the activity against the tested Gram-positive bacteria is higher than the activity against the tested Gram-negative bacteria. The optimal antibacterial activity was attained by compounds **5c** and **5d,** which showed potent and broad-spectrum antibacterial activity against all the tested bacterial strains. The inhibitory activity of the compounds against *Candida albicans* was generally lower than their antibacterial activity, compounds **4g** displayed medium activity, and all other compounds were either poorly active or inactive compared with Clotrimazole.

The minimal inhibitory concentrations (MICs) of the most active compounds **4j**, **4l**, **5a**, **5b**, **5c,** and **5d**, and the antibacterial antibiotics Gentamicin sulfate, Ampicillin trihydrate, and the antifungal drug Clotrimazole were determined by the microdilution susceptibility method in Müller–Hinton broth and Sabouraud liquid medium [64]. The MIC values were highly consistent with their growth inhibition zones.

Based on the results of the antibacterial activity, it could be concluded that the activity of the piperazinomethyl *N*-Mannich bases **5a**–**d** is superior to their arylaminomethyl analogues **4a**–**l**. Considering the anilinomethyl analogue **4a** as the basic structure of the arylaminomethyl analogues **4a**–**l**, the antibacterial activity of the monohalo derivatives **4b**–**d** was slightly improved against the tested Gram-positive bacteria. Meanwhile, the antibacterial activity of the nitro derivatives **4e**–**g** was greatly declined with general improvement of the antifungal activity. Despite the high lipophilicity of the trifluoromethyl derivatives **4h** and **4i**, the compounds only retained moderate activity against *Staphylococcus aureus* and lacked activity against the tested Gram-positive bacteria and *Candida albicans*. Introduction a difluoro- or dichlorophenyl moieties (compounds **4j**–**l**) greatly enhanced the Gram-positive antibacterial activity and these derivatives endowed moderate or marginal activity against the tested Gram-negative bacteria and *Candida albicans*.

The replacement of the arylaminomethyl moiety with a piperazinomethyl moiety greatly enhanced the antibacterial activity and the piperazinomethyl derivatives **5a**–**d** exhibited higher potency and broader antibacterial spectrum compared to their arylaminomethyl analogues **4a**–**l**, with no antifungal activity. In addition, the antibacterial activity of the piperazinomethyl derivatives seems correlated to their lipophilicity as the optimum antibacterial activity against the tested Gram-negative bacteria was displayed by compounds **5c** and **5d**.

### 2.3. In Vitro Anti-proliferative Activity

The 3-(4,5-dimethylthiazol-2-yl)-2,5-diphenyltetrazolium bromide (MTT) colorimetric assay [65,66] was adopted to evaluate the in vitro anti-proliferative activity of compounds **4a**–**l** and **5a**–**d** against five human cancer cell lines namely; prostate cancer (PC3), human colorectal cancer (HCT-116), human hepatocellular carcinoma (HePG-2), human epithelioid carcinoma (HeLa) and human breast cancer (MCF7) cell lines. The results of the anti-proliferative activity of compounds **4a**–**l**, **5a**–**d,** and the anticancer drug Doxorubicin [67] are displayed in Table 3.

According to the anti-proliferative activity results, the tested compounds exhibited variable degrees of activity against the tested cancer cell lines. In general, the activity against MCF-7 and HeLa seems higher than PC-3, HCT-116, and HePG-2. In addition, the activity of the piperazinomethyl analogues is higher than their arylaminomethyl analogues. The optimal activity was attained by compounds **4l**, **5a**, **5c,** and **5d** with IC_50_ < 25 μM against the tested cell lines.

Within the arylaminomethyl analogues **4a**–**l**, the compounds were inactive against PC3 cell lines (IC_50_ > 100 μM) or weakly active (IC_50_ = 51–100 μM) except compounds **4l,** which retained moderate activity (IC_50_ 34.60 μM). The anti-proliferative activity of the arylaminomethyl analogues **4a**–**l** seems dependent on the aryl substituents, the 2,4-dichlorophenyl is the optimal substituents while the nitrophenyl substitution at 2- or 4- position (compounds **4e**,**g**) greatly deteriorated the anti-proliferative activity. The anilinomethyl analogue **4a**, monohalophenylaminomethyl analogues **4b**–**d**, the trifluoromethylphenyl aminomethyl analogues **4h,i** and the difluorophenylaminomethyl analogues **4j,k** analogues only retained moderate anti-proliferative activity (IC_50_ = 26–50 μM) or poor activity (IC_50_ = 51–100 μM).

Concerning the anti-proliferative activity of the piperazinomethyl derivatives **5a**–**d**, the 4-phenylpiperazinomethyl derivative **5a** exhibited potent activity against HeLa and MFC7 cell lines, moderate activity against HCT-116 cell lines and weak activity against PC3 cell lines. Substitution of the phenyl group of compound **5a** with 4-fluorophenyl group (compound **5b**) dramatically deteriorated the anti-proliferative activity. The optimum antiproliferative activity was attained by the 4-benzylpiperazinomethyl derivative **5c**, which showed potent activity against all the tested cell lines. Replacement of the benzyl group of compound **5c** with 2-trifluoromethylbenzyl group (compound **5d**) reduced the anti-proliferative activity against the tested cancer cell lines.

## 3. Materials and Methods

### 3.1. General Information

Melting points (°C, uncorrected) were determined in open glass capillaries using a Barnstead 9100 electro-thermal melting point apparatus. Nuclear magnetic resonance (NMR) spectra were determined in DMSO-*d*_6_ on a JEOL ECA 500 III at 500.16 MHz for ^1^H and 125.77 MHz for ^13^C. Elemental analyses (C, H, N, and S) were in agreement with the proposed structures within ± 0.4% of the theoretical values (Table S1). Monitoring of the reactions and checking of the purity of the final products were carried out by thin layer chromatography (TLC) using silica gel pre-coated aluminum sheets (60 F_254_; Merck) and visualization with ultraviolet light (UV) at 365 and 254 nm and/or stained with an anisaldehyde solution and a phosphomolybdic acid solution. All chemicals and solvents were purchased from Alfa Aesar (Germany), and used without additional purification. The reference drugs Gentamicin sulfate (CAS 1405-41-0), Ampicillin trihydrate (CAS 7177-48-2), Clotrimazole (CAS 23593-75-1) and Doxorubicin (CAS 23214-92-8) were purchased from Sigma-Aldrich Chemie GmbH (Germany). Compound **3** was prepared according to the previously reported procedure [61].

### 3.2. Synthesis of 3-(Arylaminomethyl)-5-(3,4-Dimethoxyphenyl)-1,3,4-Oxadiazole-2(3H)-Thiones **4a–l** and 3-[(4-Substituted Piperazin-1-yl)Methyl]-5-(3,4-Dimethoxyphenyl}-1,3,4-Oxadiazole- 2(3H)-Thiones **5a–d**

The appropriate primary aromatic amine or 1-substituted piperazine (0.01 mole) and 37% formaldehyde solution (1.0 mL) were added to a hot solution of 5-(3,4-dimethoxyphenyl)-1,3,4-oxadiazole-2(3*H*)-thione **3** (1.19 g, 5.0 mmole), in ethanol (10 mL), and the mixture was stirred at room temperature for 5 h and allowed to stand overnight. Water (5 mL) was then added drop-wisely to the reaction mixture with continuous stirring for one hour. The separated precipitate was filtered, washed with water, dried, and crystallized.

*3-Anilinomethyl-5-(3,4-dimethoxyphenyl)-1,3,4-oxadiazole-2(3H)-thione***4a**. Fine colorless needle crystals. ^1^H NMR: *δ* 3.84–3.90 (m, 7H, OCH_3_ & NH), 6.01–6.09 (m, 2H, CH_2_), 6.69 (t, 1H, Ar-H, *J* = 7.0 Hz), 6.91 (d, 2H, Ar-H, *J* = 8.5 Hz), 7.08–7.33 (m, 5H, Ar-H). ^13^C NMR: *δ* 55.6, 55.8 (OCH_3_), 57.9 (CH_2_), 108.4, 112.1, 112.9, 114.0, 117.9, 120.0, 129.1, 145.3, 149.1, 152.4 (Ar-C), 159.0 (Oxadiazole C5), 175.3 (C=S).

*3-[(4-Fluorophenylamino)methyl]-5-(3,4-dimethoxyphenyl)-1,3,4-oxadiazole-2(3H)-thione***4b**. Fine colorless needle crystals. ^1^H NMR: *δ* 3.86–3.90 (m, 7H, OCH_3_ & NH), 5.49 (d, 2H, CH_2_, *J* = 7.0 Hz), 6.89–6.94 (m, 2H, Ar-H), 6.99–7.06 (m, 2H, Ar-H), 7.21–7.25 (m, 1H, Ar-H), 7.32 (d, 1H, Ar-H, *J* = 1.5 Hz), 7.51 (d, 1H, Ar-H, *J* = 1.5 Hz). ^13^C NMR: *δ* 55.7, 55.8 (OCH_3_), 58.3 (CH_2_), 108.4, 112.1, 114.0 (d, *J*_C-F_ = 5.0 Hz), 115.6 (d, *J*_C-F_ = 23.0 Hz), 119.1 (d, *J*_C-F_ = 7.0 Hz), 120.1, 141.9, 149.1, 152.4, 155.4 (d, *J*_C-F_ = 232.5 Hz) (Ar-C), 159.0 (Oxadiazole C5), 175.4 (C=S).

*3-[(3-Chlorophenylamino)methyl]-5-(3,4-dimethoxyphenyl)-1,3,4-oxadiazole-2(3H)-thione***4c**. Fine colorless needle crystals. ^1^H NMR: *δ* 3.87–3.88 (m, 7H, OCH_3_ & NH), 5.50 (d, 2H, CH_2_, *J* = 7.0 Hz), 6.73 (d, 1H, Ar-H, *J* = 1.5 Hz), 6.87 (d, 1H, Ar-H, *J* = 1.5 Hz), 7.02–7.05 (m, 1H, Ar-H), 7.17–7.19 (m, 1H, Ar-H), 7.33 (d, 1H, Ar-H, *J* = 1.5 Hz), 7.51 (d, 1H, Ar-H, *J* = 1.5 Hz), 7.55 (t, 1 H, Ar-H, *J* = 7.0 Hz). ^13^C NMR: *δ* 55.6, 55.8 (OCH_3_), 57.5 (CH_2_), 108.4, 111.8, 112.1, 112.5, 113.9, 117.5, 120.1, 130.7, 133.8, 147.1, 149.1, 152.4 (Ar-C), 159.1 (Oxadiazole C5), 175.4 (C=S).

*3-[(4-Chlorophenylamino)methyl]-5-(3,4-dimethoxyphenyl)-1,3,4-oxadiazole-2(3H)-thione***4d**. Fine colorless needle crystals. ^1^H NMR: *δ* 3.87–3.88 (s, 7H, OCH_3_ & NH), 5.49 (d, 2H, CH_2_, *J* = 7.0 Hz), 6.93 (d, 2H, Ar-H, *J* = 9.0 Hz), 7.20–7.22 (m, 2H, Ar-H), 7.32 (d, 1 H, Ar-H, *J* = 1.5 Hz), 7.42 (d, 1 H, Ar-H, *J* = 8.0 Hz), 7.50 (d, 1 H, Ar-H, *J* = 1.5, 8.0 Hz). ^13^C NMR: *δ* 55.7, 55.8 (OCH_3_), 57.8 (CH_2_), 108.4, 112.1, 113.9, 114.5, 120.1, 121.5, 128.8, 144.4, 149.1, 152.4, 159.1 (Oxadiazole C5), 175.4 (C=S).

*3-[(2-Nitrophenylamino)methyl]-5-(3,4-dimethoxyphenyl)-1,3,4-oxadiazole-2(3H)-thione***4e**. Pale yellow block crystals. ^1^H NMR: *δ* 3.85–3.89 (m, 7H, OCH_3_ & NH), 5.77 (d, 2H, CH_2_, *J* = 7.0 Hz), 6.89–6.95 (m, 1H, Ar-H), 7.16–7.21 (m, 1H, Ar-H), 7.44–7.48 (m, 1H, Ar-H), 7.49–7.54 (m, 1H, Ar-H), 7.65–7.70 (m, 1H, Ar-H), 8.16 (d, 1H, Ar-H, *J* = 1.5 Hz), 8.85–8.91 (m, 1H, Ar-H). ^13^C NMR: *δ* 55.7, 55.8 (OCH_3_), 56.6 (CH_2_), 108.5, 112.1, 115.4, 117.9, 119.8, 120.0, 125.4, 126.5, 136.6, 142.2, 149.2, 152.5 (Ar-C), 159.3 (Oxadiazole C5), 175.5 (C=S).

*3-[(3-Nitrophenylamino)methyl]-5-(3,4-dimethoxyphenyl)-1,3,4-oxadiazole-2(3H)-thione***4f**. Pale yellow block crystals. ^1^H NMR: *δ* 3.86–3.90 (m, 7H, OCH_3_ & NH), 5.59 (d, 2H, CH_2_, *J* = 7.0 Hz), 7.19 (d, 1H, Ar-H, *J* = 9.0 Hz), 7.32–7.36 (m, 1H, Ar-H), 7.45 (t, 1 H, Ar-H, *J* = 8.0 Hz), 7.51 (d, 1H, Ar-H, *J* = 1.5 Hz), 7.55 (d, 1H, Ar-H, *J* = 1.5 Hz), 7.87–7.94 (m, 2H, Ar-H). ^13^C NMR: *δ* 55.6, 55.8 (OCH_3_), 57.4 (CH_2_), 106.9, 108.4, 112.0, 112.4, 113.9, 119.6, 120.1, 130.2, 146.9, 148.8, 149.1, 152.4 (Ar-C), 159.0 (Oxadiazole C5), 175.5 (C=S).

*3-[(4-Nitrophenylamino)methyl]-5-(3,4-dimethoxyphenyl)-1,3,4-oxadiazole-2(3H)-thione***4g**. Pale yellow block crystals. ^1^H NMR: *δ* 3.86–3.90 (m, 7H, OCH_3_ & NH), 5.61 (d, 2H, CH_2_, *J* = 7.0 Hz), 7.07 (d, 2H, Ar-H, *J* = 9.0 Hz), 7.19 (d, 1H, Ar-H, *J* = 8.5 Hz), 7.33 (d, 1H, Ar-H, *J* = 2.0 Hz), 7.52 (d, 1H, Ar-H, *J* = 2.0 Hz), 8.12 (d, 2H, Ar-H, *J* = 9.0 Hz). ^13^C NMR: *δ* 55.7, 55.8 (OCH_3_), 56.7 (CH_2_), 108.5, 112.1, 112.5, 113.9, 120.2, 126.0, 138.1, 149.1, 152.1, 152.5 (Ar-C), 159.2 (Oxadiazole C5), 175.5 (C=S).

*3-[(2-Trifluoromethylphenylamino)methyl]-5-(3,4-dimethoxyphenyl)-1,3,4-oxadiazole-2(3H)-thione***4h**. Colorless prism crystals. ^1^H NMR: *δ* 3.86–3.90 (m, 7H, OCH_3_ & NH), 5.61 (d, 2H, CH_2_, *J* = 6.5 Hz), 6.89 (t, 2H, Ar-H, *J* = 8.0 Hz), 7.24 (d, 2H, Ar-H, *J* = 9.0 Hz), 7.31–7.37 (m, 2H, Ar-H), 7.50–7.52 (m, 2H, Ar-H). ^13^C NMR: *δ* 55.7, 55.8 (OCH_3_), 57.3 (CH_2_), 108.5, 112.0, 114.6, 115.0, 116.7, 117.8, 119.8, 120.1, 126.6, 133.0, 133.7, 149.1, 152.1 (Ar-C & CF_3_), 160.6 (Oxadiazole C5), 177.2 (C=S).

*3-[(3-Trifluoromethylphenylamino)methyl]-5-(3,4-dimethoxyphenyl)-1,3,4-oxadiazole-2(3H)-thione***4i**. White amorphous powder. ^1^H NMR: *δ* 3.86–3.90 (m, 7H, OCH_3_ & NH), 5.56 (d, 2H, CH_2_, *J* = 7.5 Hz), 7.02 (d, 1H, Ar-H, *J* = 7.5 Hz), 7.17–7.21 (m, 1H, Ar-H), 7.31–7.34 (m, 2H, Ar-H), 7.37–7.42 (m, 1H, Ar-H), 7.51 (d, 1H, Ar-H, *J* = 1.5 Hz), 7.71 (t, 1 H, Ar-H, *J* = 7.5 Hz). ^13^C NMR: *δ* 55.6, 55.8 (OCH_3_), 57.4 (CH_2_), 108.3, 109.0, 112.0, 113.9, 115.4, 117.0, 120.1, 123.3, 125.5, 130.1, 146.2, 149.1, 152.4 (Ar-C & CF_3_), 159.0 (Oxadiazole C5), 175.5 (C=S).

*3-[(2,4-Difluorophenylamino)methyl]-5-(3,4-dimethoxyphenyl)-1,3,4-oxadiazole-2(3H)-thione***4j**. Colorless needle crystals. ^1^H NMR: *δ* 3.86-3.90 (m, 7H, OCH_3_ & NH), 5.52 (d, 2H, CH_2_, *J* = 7.0 Hz), 6.95–7.04 (m, 2H, Ar-H), 7.17–7.21 (m, 2H, Ar-H), 7.32 (d, 1H, Ar-H, *J* = 1.5 Hz), 7.49–7.52 (m, 1H, Ar-H). ^13^C NMR: *δ* 55.6, 55.8 (OCH_3_), 57.9 (CH_2_), 104.0 (d, *J*_C-F_ = 23.05 Hz), 108.4, 111.0 (d, *J*_C-F_ = 19.0 Hz), 112.0, 113.8 (d, *J*_C-F_ = 8.5 Hz), 113.9, 120.1, 130.2 (d, *J*_C-F_ = 13.0 Hz), 149.1, 152.4, 154.3 (d, *J*_C-F_ = 225.5 Hz), 159.7 d, *J*_C-F_ = 237.5 Hz) (Ar-C), 159.2 (Oxadiazole C5), 175.4 (C=S).

*3-[(2,5-Difluorophenylamino)methyl]-5-(3,4-dimethoxyphenyl)-1,3,4-oxadiazole-2(3H)-thione***4k**. Colorless needle crystals. ^1^H NMR: *δ* 3.86–3.90 (m, 7H, OCH_3_ & NH), 5.51 (d, 2H, CH_2_, *J* = 6.5 Hz), 7.02–7.07 (m, 1H, Ar-H), 7.12–7.16 (m, 1H, Ar-H), 7.18–7.21 (m, 1H, Ar-H), 7.33 (d, 1H, Ar-H, *J* = 1.5 Hz), 7.39–7.44 (m, 1H, Ar-H), 7.49–7.53 (m, 1H, Ar-H). ^13^C NMR: *δ* 55.7, 55.8 (OCH_3_), 57.1 (CH_2_), 100.5 (d, *J*_C-F_ = 27.5 Hz), 103.2 (d, *J*_C-F_ = 7.0 Hz), 108.4, 111.9, 112.0, 114.6, 115.6 (d, *J*_C-F_ = 11.0 Hz), 119.8, 120.1, 149.1, 152.1, 159.0 (d, *J*_C-F_ = 241.0 Hz) (Ar-C), 159.7 (Oxadiazole C5), 175.5 (C=S).

*3-[(2,4-Dichlorophenylamino)methyl]-5-(3,4-dimethoxyphenyl)-1,3,4-oxadiazole-2(3H)-thione***4l**. Fine colorless needle crystals. ^1^H NMR: *δ* 3.86–3.90 (m, 7H, OCH_3_ & NH), 5.58 (d, 2H, CH2, *J* = 7.0 Hz), 7.04–7.08 (m, 1H, Ar-H), 7.16–7.20 (m, 1H, Ar-H), 7.28–7.33 (m, 2H, Ar-H), 7.47–7.52 (m, 2H, Ar-H). ^13^C NMR: *δ* 55.7, 55.8 (OCH_3_), 57.4 (CH_2_), 108.5, 112.0, 113.8, 113.9, 119.1, 120.1, 121.7, 127.9, 128.8, 140.5, 149.1, 152.4 (Ar-C), 159.2 (Oxadiazole C5), 175.4 (C=S).

*5-(3,4-Dimethoxyphenyl)-3-[(4-phenylpiperazin-1-yl)methyl]-1,3,4-oxadiazole-2(3H)-thione***5a**. Fine colorless needle crystals. ^1^H NMR: *δ* 2.89-2.99 (m, 4H, Piperazine-H), 3.13–3.23 (m, 4H, Piperazine-H), 3.89 (s, 6H, OCH_3_), 5.13 (s, 2H, CH_2_), 6.80 (t, 1H, Ar-H, *J* = 7.0 Hz), 6.96 (d, 2H, Ar-H, *J* = 8.0 Hz), 7.18–7.26 (m, 3H, Ar-H), 7.36–7.39 (m, 1H, Ar-H), 7.55 (d, 1H, Ar-H, *J* = 8.0 Hz). ^13^C NMR: *δ* 48.3, 49.6 (Piperazine-C), 55.7, 55.8 (OCH_3_), 69.7 (CH_2_), 108.5, 112.0, 114.1, 115.7, 119.0, 120.1, 128.9, 149.1, 151.0, 152.3 (Ar-C), 158.7 (Oxadiazole C5), 177.2 (C=S).

*5-(3,4-Dimethoxyphenyl)-3-{4-[(4-fluorophenyl)piperazin-1-yl]methyl}-1,3,4-oxadiazole-2(3H)-thione***5b**. Colorless needle crystals. ^1^H NMR: *δ* 2.92–2.95 (m, 4H, Piperazine-H), 3.09–3.16 (m, 4H, Piperazine-H), 3.89 (s, 6H, OCH_3_), 5.12 (s, 2H, CH_2_), 6.96–6.98 (m, 2H, Ar-H), 7.05-7.07 (m, 1H, Ar-H), 7.08-7.19 (m, 1H, Ar-H), 7.21 (d, 1H, Ar-H, *J* = 1.5 Hz), 7.37 (d, 2H, Ar-H, *J* = 8.0 Hz). ^13^C NMR: *δ* 49.1, 49.6 (Piperazine-C), 55.7, 55.8 (OCH_3_), 69.7 (CH_2_), 108.5, 112.0, 114.2, 115.3 (d, *J*_C-F_ = 21.5 Hz), 117.4 (d, *J*_C-F_ = 7.0 Hz), 120.1, 147.0, 149.1, 152.3, 156.1 (d, *J*_C-F_ = 236.5 Hz) (Ar-C),158.6 (Oxadiazole C5), 177.2 (C=S).

*5-(3,4-Dimethoxyphenyl)-3-[(4-benzylpiperazin-1-yl)methyl]-1,3,4-oxadiazole-2(3H)-thione***5c**. White amorphous powder. ^1^H NMR: *δ* 2.30–2.50 (m, 4H, Piperazine-H), 2.74–2.88 (m, 4H, Piperazine-H), 3.50 (s, 2H, Benzylic CH_2_), 3.89 (s, 6H, OCH_3_), 5.05 (s, 2H, CH_2_), 7.19 (d, 1H, Ar-H, *J* = 9.0 Hz), 7.24–7.41 (m, 6H, Ar-H), 7.53 (d, 1H, Ar-H, *J* = 8.0 Hz). ^13^C NMR: *δ* 49.5, 52.3 (Piperazine-C), 55.7, 55.8 (OCH_3_), 61.9 (Benzylic CH_2_), 69.7 (CH_2_), 108.5, 112.0, 114.3, 120.0, 127.0, 128.1, 128.8, 137.8, 149.1, 152.2 (Ar-C), 158.6 (Oxadiazole C5), 177.2 (C=S).

*5-(3,4-Dimethoxyphenyl)-3-{4-[(2-trifluorobenzyl)piperazin-1-yl]methyl}-1,3,4-oxadiazole-2(3H)-thione***5d**. Colorless needle crystals. ^1^H NMR: *δ* 2.38–2.52 (m, 4H, Piperazine-H), 2.78-2.90 (m, 4H, Piperazine-H), 3.64 (s, 2H, Benzylic CH_2_), 3.89 (s, 6H, OCH_3_), 5.06 (s, 2H, CH_2_), 7.20 (d, 1H, Ar-H, *J* = 8.0 Hz), 7.37 (d, 1H, Ar-H, *J* = 1.5 Hz), 7.47 (t, 1H, Ar-H, *J* = 7.0 Hz), 7.55 (d, 1H, Ar-H, *J* = 8.0 Hz), 7.64 (t, 1H, Ar-H, *J* = 8.0 Hz), 7.71 (d, 1H, Ar-H, *J* = 8.0 Hz), 7.76 (d, 1H, Ar-H, *J* = 8.0 Hz). ^13^C NMR: *δ* 49.7, 52.6 (Piperazine-C), 55.7, 55.8 (OCH_3_), 57.5 (Benzylic CH_2_), 69.8 (CH_2_), 108.5, 112.0, 114.2, 118.5, 120.1, 123.4, 125.7, 127.3, 130.3, 132.4, 137.2, 149.1, 152.3 (Ar-C & CF_3_), 158.6 (Oxadiazole C5), 177.2 (C=S).

## 4. Conclusions

Sixteen 1,3,4-oxadiazole-linked *N*-Mannich bases namely; 3-arylaminomethyl-5-(3,4- dimethoxyphenyl)-1,3,4-oxadiazole-2(3*H*)-thiones **4a**–**l** and 3-[(4-substituted piperazin-1- yl)-methyl]-5-(3,4-dimethoxyphenyl)-1,3,4-oxadiazole-2(3*H*)-thiones **5a**–**d** were synthesized and their structures were confirmed by ^1^H NMR, ^13^C NMR and elemental analysis. The in vitro inhibitory activity of compounds **4a**–**l** and **5a**–**d** was assessed against a panel of standard pathogenic Gram-positive bacteria, Gram-negative bacteria, and the yeast-like pathogenic fungus *Candida albicans*. Compounds **5c** and **5d** displayed potent broad spectrum antibacterial activities and compounds **4j**, **4l**, **5a,** and **5b** showed potent activity against the tested Gram-positive bacteria. The anti-proliferative activity of the compounds was evaluated against prostate cancer (PC3), human colorectal cancer (HCT-116), human hepatocellular carcinoma (HePG-2), human epithelioid carcinoma (HeLa), and human breast cancer (MCF7) cell lines. Compounds **4l**, **5a**, **5c,** and **5d** showed potent inhibition of cell proliferation in almost all the tested cancer cell lines. The prepared 1,3,4-oxadiazole-linked *N*-Mannich bases could be considered good antibacterial and anticancer drug candidates. The biological testing results are considered as preliminary and further investigations, including experimental and theoretical investigations for the exploration of their targets, are required for optimization of their chemotherapeutic activities.

## Figures and Tables

**Table 1:** Crystallization solvents, melting points (M.P.), yield percentages, molecular (Mol.) formulae, and molecular weights (Wt.) of compounds **4a**–**l** and **5a**–**d**.

Compound No.	X/R	Crystallization Solvents	M.P. (°C)	Yield (%)	Mol. Formula (Mol. Wt.)
**4a**	H	EtOH/H_2_O	147–149	82	C_17_H_17_N_3_O_3_S (343.40)
**4b**	4-F	EtOH/H_2_O	135–137	84	C_17_H_16_FN_3_O_3_S (361.39)
**4c**	3-Cl	EtOH	143–145	77	C_17_H_16_ClN_3_O_3_S (377.85)
**4d**	4-Cl	EtOH	166–168	79	C_17_H_16_ClN_3_O_3_S (377.85)
**4e**	2-NO_2_	EtOH/CHCl_3_	211–213	88	C_17_H_16_N_4_O_5_S (388.40)
**4f**	3-NO_2_	EtOH/CHCl_3_	181–183	85	C_17_H_16_N_4_O_5_S (388.40)
**4g**	4-NO_2_	EtOH/CHCl_3_	222–224	92	C_17_H_16_N_4_O_5_S (388.40)
**4h**	2-CF_3_	EtOH/H_2_O	220–222	90	C_18_H_16_F_3_N_3_O_3_S (411.40)
**4i**	3-CF_3_	EtOH/H_2_O	206–208	86	C_18_H_16_F_3_N_3_O_3_S (411.40)
**4j**	2,4-F_2_	EtOH	169–171	92	C_17_H_15_F_2_N_3_O_3_S (379.38)
**4k**	2,5-F_2_	EtOH	212–214	90	C_17_H_15_F_2_N_3_O_3_S (379.38)
**4l**	2,4-Cl_2_	EtOH	227–229	94	C_17_H_15_Cl_2_N_3_O_3_S (412.29)
**5a**	C_6_H_5_	EtOH	151–153	85	C_21_H_24_N_4_O_3_S (412.51)
**5b**	4-FC_6_H_4_	EtOH	118–120	78	C_21_H_23_FN_4_O_3_S (430.50)
**5c**	C_6_H_5_CH_2_	EtOH/H_2_O	121–123	75	C_22_H_26_N_4_O_3_S (426.53)
**5d**	2-CF_3_C_6_H_4_CH_2_	EtOH	141–143	89	C_23_H_25_F_3_N_4_O_3_S (494.53)

**Table 2:** In vitro activity of compounds **4a**–**l** and **5a**–**d** (200 μg/8 mm disc); the broad-spectrum antibacterial drugs Gentamicin sulfate, Ampicillin trihydrate, and the antifungal drug Clotrimazole (100 μg/8 mm disc) against *Staphylococcus aureus* American type culture collection (ATCC) 6571 (SA), *Bacillus subtilis* ATCC 5256 (BS), *Micrococcus luteus* ATCC 27,141 (ML), *Escherichia coli* ATCC 8726 (EC), *Pseudomonas aeruginosa* ATCC 27,853 (PA), and the yeast-like pathogenic fungus *Candida albicans* MTCC 227 (CA).

Comp. No.	C log *P*^c^	Diameter of Growth Inhibition Zone (mm) ^a^					
		SA	BS	ML	EC	PA	CA
**4a**	3.701	14	13	15	-	-	10
**4b**	4.146	15	16	17	-	-	12
**4c**	4.716	14	17	13	-	-	-
**4d**	4.716	15	13	14	-	-	-
**4e**	4.242	12	11	10	-	-	13
**4f**	4.092	11	12	-	-	-	12
**4g**	4.092	13	12	11	-	-	16
**4h**	5.113	15	12	-	-	-	-
**4i**	5.113	16	12	-	-	-	-
**4j**	4.395	**19 (4) ^b^**	12	16	12	13	11
**4k**	4.395	17	14	15	14	-	12
**4l**	5.535	**19 (4) ^b^**	**21 (2) ^b^**	**18 (16) ^b^**	15	12	11
**5a**	3.789	**23 (1) ^b^**	**26 (1) ^b^**	**21 (1) ^b^**	14	13	-
**5b**	4.103	**20 (8) ^b^**	**23 (1) ^b^**	**20 (1) ^b^**	16	17	-
**5c**	4.712	**26 (1) ^b^**	**30 (0.5) ^b^**	**22 (1) ^b^**	**19 (4) ^b^**	**18 (4) ^b^**	-
**5d**	5.595	**28 (1) ^b^**	**29 (1) ^b^**	**26 (1) ^b^**	**22 (1) ^b^**	**20 (2) ^b^**	-
Gentamicin sulfate		**27 (1) ^b^**	**26 (2) ^b^**	**20 (2) ^b^**	**22 (0.5) ^b^**	**21 (0.5) ^b^**	NT
Ampicillin trihydrate		**22 (2) ^b^**	**23 (1) ^b^**	**20 (2) ^b^**	**16 (8) ^b^**	**16 (8) ^b^**	NT
Clotrimazole		NT	NT	NT	NT	NT	**21 (4) ^b^**

^a^ (-): inactive (inhibition zone < 10 mm), ^b^ Figures shown in parentheses represent the minimal inhibitory concentration (MIC) values (μg/mL), ^c^ Calculated using the CS ChemOffice Ultra version 8.0, CambridgeSoft, Cambridge, MA, USA), NT: not tested. SA, *Staphylococcus aureus*; BS, *Bacillus subtilis*; ML, *Micrococcus luteus*; EC, *Escherichia coli*; PA, *Pseudomonas aeruginosa*; CA, *Candida albicans*. High activity (>18 mm) values have been bolded for emphasis.

**Table 3:** In vitro anti-proliferative activity of the tested compounds **4a**–**l**, **5a**–**d,** and Doxorubicin expressed as IC_50_ values against prostate cancer (PC3), human colorectal cancer (HCT-116), human hepatocellular carcinoma (HePG-2), human epithelioid carcinoma (HeLa), and MCF7 human cancer cell line.

Comp. No.	IC_50_ (µM) ^a^				
	PC3	HCT-116	HePG-2	HeLa	MCF7
**4a**	63.94 ± 3.8	53.17 ± 3.3	47.23 ± 3.1	28.31 ± 2.0	55.34 ± 2.8
**4b**	71.80 ± 4.0	77.52 ± 4.1	56.34 ± 3.3	49.47 ± 3.0	68.26 ± 3.4
**4c**	84.52 ± 4.8	39.44 ± 2.6	78.86 ± 4.1	44.69 ± 2.8	33.86 ± 2.3
**4d**	78.35 ± 4.5	48.30 ± 3.0	73.80 ± 3.9	54.02 ± 3.2	40.52 ± 2.5
**4e**	>100	92.11 ± 4.9	>100	75.61 ± 3.9	88.33 ± 3.9
**4f**	74.67 ± 4.4	69.38 ± 3.8	58.41 ± 3.5	57.26 ± 2.5	65.35 ± 3.2
**4g**	>100	92.11 ± 4.9	>100	75.61 ± 3.9	88.33 ± 3.9
**4h**	59.48 ± 3.5	35.01 ± 2.7	42.74 ± 2.9	31.72 ± 2.2	29.10 ± 2.1
**4i**	59.48 ± 3.5	35.01 ± 2.7	42.74 ± 2.9	31.72 ± 2.2	29.10 ± 2.1
**4j**	95.61 ± 5.1	64.07 ± 3.5	86.45 ± 4.5	61.98 ± 3.5	59.87 ± 2.7
**4k**	75.22 ± 4.1	61.07 ± 3.4	77.40 ± 3.6	49.55 ± 3.2	29.56 ± 3.6
**4l**	34.60 ± 2.3	**19.95 ± 1.8**	**17.42 ± 1.4**	**10.96 ± 1.1**	**12.97 ± 1.0**
**5a**	52.53 ± 3.3	27.49 ± 2.3	36.08 ± 2.5	**24.09 ± 1.8**	**17.80 ± 1.3**
**5b**	>100	89.26 ± 4.6	91.78 ± 4.9	67.53 ± 3.7	79.16 ± 3.6
**5c**	**23.92 ± 1.9**	**14.69 ± 1.2**	**11.93 ± 1.0**	**9.50 ± 0.8**	**6.49 ± 0.4**
**5d**	38.02 ± 2.5	32.81 ± 2.6	**22.91 ± 1.6**	**18.37 ± 1.4**	**24.33 ± 1.9**
**Doxorubicin**	**8.87 ± 0.6**	**5.23 ± 0.3**	**4.50 ± 0.2**	**5.57 ± 0.4**	**4.17 ± 0.2**

^a^ IC_50_ values presented as the mean ± SD of three separate determinations. Significant values (<25 µm) have been bolded for emphasis.

## Supplementary Materials

The micro-analytical data (C, H, N, and S), the experimental details of the determination of in vitro antimicrobial activity, in vitro anti-proliferative activity, and the NMR spectra can be found online.

## Sample Availability

Samples of the compounds **4a**–**l** and **5a**–**d** are available from the corresponding author.
