# Supplementary material for: 1,3,4-Oxadiazole N-Mannich Bases: Synthesis, Antimicrobial, and Anti-Proliferative Activities
Source: Molecules. 2021 Apr 7;26(8):2110. doi: 10.3390/molecules26082110 (PMC8067589; doi:10.3390/molecules26082110)
Supplement: Supplementary file 1 [file molecules-26-02110-s001.pdf]

**Supplementary Materials**

# 1,3,4-Oxadiazole *N*-Mannich Bases: Synthesis, Antimicrobial, and Anti-Proliferative Activities

**Table S1.** Molecular formulae, molecular weights and elemental analyses data of compounds **4a-l** and **5a-d**.

| Comp. No. | Mol. Formula (Mol. Wt.) | Analysis: % Calcd. (Found) | | | |
|---|---|---|---|---|---|
| | | C | H | N | S |
| **4a** | $C_{17}H_{17}N_3O_3S$ (343.40) | 59.46 (59.23) | 4.99 (5.02) | 12.24 (12.13) | 9.34 (9.33) |
| **4b** | $C_{17}H_{16}FN_3O_3S$ (361.39) | 56.50 (56.44) | 4.46 (4.52) | 11.63 (11.60) | 8.87 (8.85) |
| **4c** | $C_{17}H_{16}ClN_3O_3S$ (377.85) | 54.04 (53.88) | 4.27 (4.29) | 11.12 (11.02) | 8.49 (8.50) |
| **4d** | $C_{17}H_{16}ClN_3O_3S$ (377.85) | 54.04 (54.0) | 4.27 (4.32) | 11.12 (11.04) | 8.49 (8.47) |
| **4e** | $C_{17}H_{16}N_4O_5S$ (388.40) | 52.57 (52.44) | 4.15 (4.22) | 14.43 (14.41) | 8.26 (8.12) |
| **4f** | $C_{17}H_{16}N_4O_5S$ (388.40) | 52.57 (52.50) | 4.15 (4.18) | 14.43 (14.43) | 8.26 (8.25) |
| **4g** | $C_{17}H_{16}N_4O_5S$ (388.40) | 52.57 (52.52) | 4.15 (4.23) | 14.43 (14.25) | 8.26 (8.22) |
| **4h** | $C_{18}H_{16}F_3N_3O_3S$ (411.40) | 52.55 (52.32) | 3.92 (4.02) | 10.21 (10.22) | 7.79 (7.60) |
| **4i** | $C_{18}H_{16}F_3N_3O_3S$ (411.40) | 52.55 (52.48) | 3.92 (3.94) | 10.21 (10.15) | 7.79 (7.72) |
| **4j** | $C_{17}H_{15}F_2N_3O_3S$ (379.38) | 53.82 (53.72) | 3.99 (4.04) | 11.08 (11.01) | 8.45 (8.44) |
| **4k** | $C_{17}H_{15}F_2N_3O_3S$ (379.38) | 53.82 (53.66) | 3.99 (4.12) | 11.08 (10.88) | 8.45 (8.45) |
| **4l** | $C_{17}H_{15}Cl_2N_3O_3S$ (412.29) | 49.52 (49.34) | 3.67 (3.75) | 10.19 (10.20) | 7.78 (7.76) |
| **5a** | $C_{21}H_{24}N_4O_3S$ (412.51) | 61.14 (61.14) | 5.86 (5.92) | 13.58 (13.42) | 7.77 (7.68) |
| **5b** | $C_{21}H_{23}FN_4O_3S$ (430.50) | 58.59 (58.44) | 6.96 (6.99) | 5.52 (5.38) | 6.32 (6.30) |
| **5c** | $C_{22}H_{26}N_4O_3S$ (426.53) | 61.95 (61.72) | 6.14 (6.30) | 13.14 (13.0) | 7.52 (7.48) |
| **5d** | $C_{23}H_{25}F_3N_4O_3S$ (494.53) | 55.86 (55.64) | 5.10 (5.18) | 11.33 (11.24) | 6.48 (6.44) |

## Determination of *in vitro* antimicrobial activity (agar disc diffusion method) [1]

Sterile filter paper discs (8 mm diameter) were moistened with the compound solution in dimethyl sulfoxide of specific concentration (200 μg/disc), and the antibacterial drugs, Gentamicin sulphate and Ampicillin trihydrate (100 μg/disc) and the antifungal drug Clotrimazole (100 μg/disc), were carefully placed on agar culture plates that had been previously inoculated separately with the microorganisms. The plates were incubated at 37 °C, and the diameters of the growth inhibition zones were measured after 24 hours for bacteria and 48 hours for *C. albicans*.

**Determination of the minimal inhibitory concentration (MIC) [2]**

Compounds **4j**, **4l**, **5a**, **5b**, **5c**, **5d**, Gentamicin sulfate, Ampicillin trihydrate, and Clotrimazole were dissolved in dimethyl sulphoxide at a concentration of 128 μg/mL. The two-fold dilutions of the solution were prepared (128, 64, 32, …, 0.25 μg/mL). Suspensions of the microorganisms at concentrations of 106 colony-forming units per mL were inoculated in the corresponding wells. The plates were then incubated at 36°C for 24 hours. The MIC values were determined as the lowest concentrations that completely inhibited visible growth of the microorganism as detected by the unaided eye.

**Determination of *in vitro* anti-proliferative activity [3,4]**

The *in vitro* anti-proliferative activity screening of compounds **4a-l** and **5a-d** was measured against three human cancer cell lines namely; prostate cancer (PC3), human colorectal cancer (HCT-116), human hepatocellular carcinoma (HePG-2), human epitheliod carcinoma (HeLa) and human breast cancer (MCF7) cell lines using the standard MTT assay. PC3, HCT-116, HePG-2, HeLa and MCF7 cells (3000 cells per well) were cultured and seeded into 96-well plates and the plates were incubated for 24 hours. The cells were then treated with compounds **4a-l**, **5a-d** and Doxorubicin at different concentrations in DMSO (0.1 μM to 100 μM) at 37 °C in an atmosphere of 5% $CO_2$ for 48 hours. Freshly prepared MTT was added to each well at a terminal concentration of 5 μg/mL and incubated with cells at 37 °C for 4 hours. The formazan crystals were dissolved in 100 μL of DMSO in each well, and the absorbency at 492 nm (for absorbance of MTT formazan) and 630 nm (for the reference wavelength) was measured with an enzyme linked immunosorbent assay (ELISA) reader (ChroMate-4300, FL, USA). All compounds were tested three times in each of the cell lines. The $IC_{50}$ values were calculated according to the equation for Boltzmann sigmoidal concentration response curve using the nonlinear regression fitting models (Graph Pad, Prism Version 5). The results reported are means of

three separate experiments. Statistical differences were analysed according to one way ANOVA test wherein the differences were considered to be significant at $p < 0.05$.

## References

1. Woods, G.L.; Washington, J.A. Antibacterial susceptibility tests: dilution and disk diffusion methods. In *Manual of Clinical Microbiology*, Murray, P.R., Baron, E.J., Pfaller, M.A., Tenover, F.C., Yolken, R.H., Eds.; American Society of Microbiology: Washington, DC, USA, 1995.
2. National Committee for Clinical Laboratory Standards. *Approved standard document M-7A*; NCCS: Villanova, PA, USA, 1985.
3. Mosmann, T. Rapid colorimetric assay for cellular growth and survival: Application to proliferation and cytotoxicity assays. *J. Immunol. Methods* **1983**, *65*, 55-63. DOI: 10.1016/0022-1759(83)90303-4.
4. Berridge, M.V.; Tan, A.S. Characterisation of the cellular reduction of 3-(4,5-dimethylthiazol-2-yl)-2,5-diphenyltetrazolium bromide (MTT): Subcellular localization, substrate dependence, and involvement of mitochondrial electron transport in MTT reduction. *Arch. Biochem. Biophys.* **1993**, *303*, 474-482, DOI: 10.1006/abbi.1993.1311.

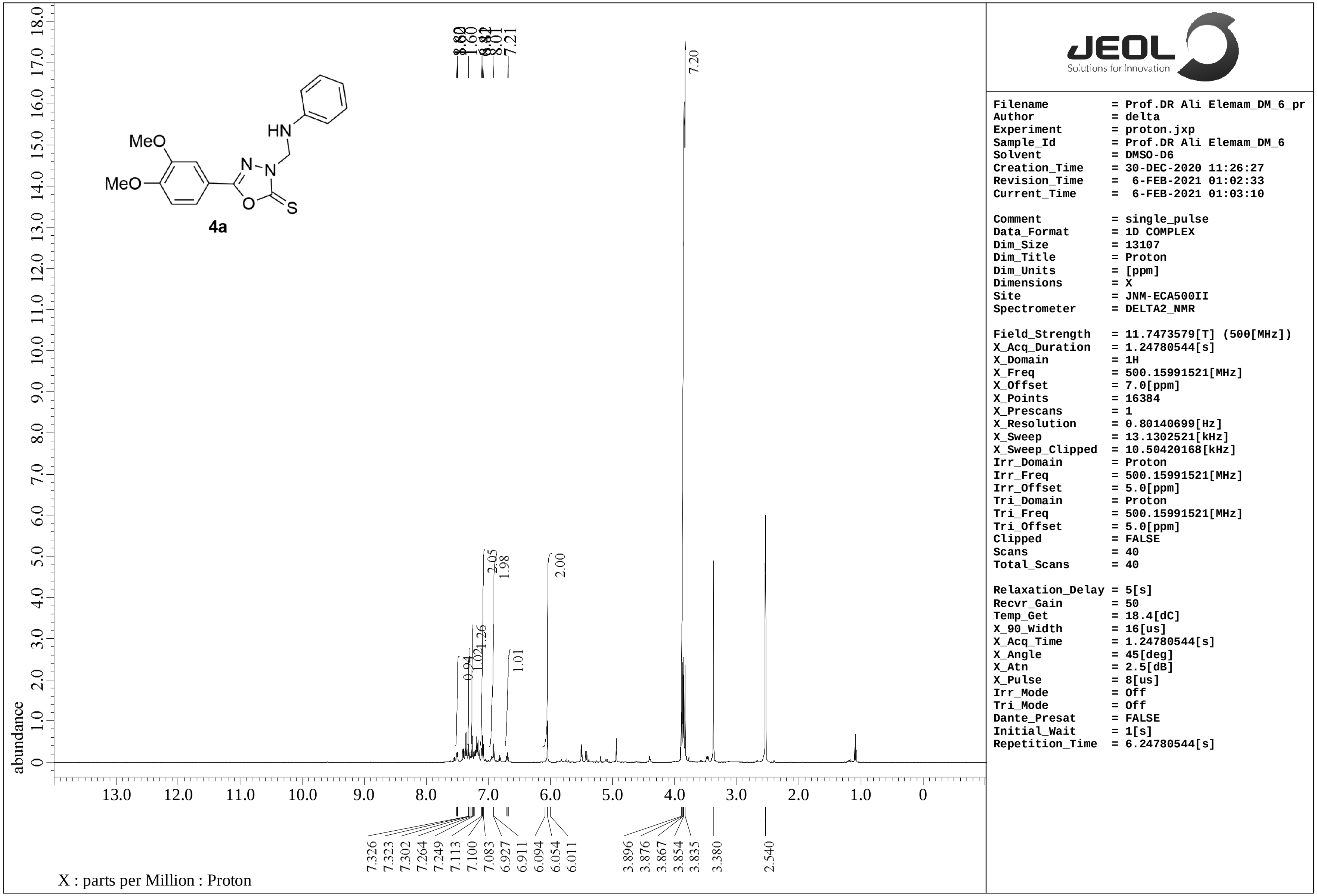
MeO
MeO
N
N
O
S
HN
4a
abundance
X : parts per Million : Proton
7.326 7.323 7.302 7.264 7.249 7.113 7.100 7.083 6.927 6.911 6.094 6.054 6.011 3.896 3.876 3.867 3.854 3.835 3.380 2.540
0.94 1.02 1.26 2.05 1.98 1.01 2.00 7.20
JEOL
Solutions for Innovation
Filename = Prof.DR Ali Elemam_DM_6_pr
Author = delta
Experiment = proton.jxp
Sample_Id = Prof.DR Ali Elemam_DM_6
Solvent = DMSO-D6
Creation_Time = 30-DEC-2020 11:26:27
Revision_Time = 6-FEB-2021 01:02:33
Current_Time = 6-FEB-2021 01:03:10
Comment = single_pulse
Data_Format = 1D COMPLEX
Dim_Size = 13107
Dim_Title = Proton
Dim_Units = [ppm]
Dimensions = X
Site = JNM-ECA500II
Spectrometer = DELTA2_NMR
Field_Strength = 11.7473579[T] (500[MHz])
X_Acq_Duration = 1.24780544[s]
X_Domain = 1H
X_Freq = 500.15991521[MHz]
X_Offset = 7.0[ppm]
X_Points = 16384
X_Prescans = 1
X_Resolution = 0.80140699[Hz]
X_Sweep = 13.1302521[kHz]
X_Sweep_Clipped = 10.50420168[kHz]
Irr_Domain = Proton
Irr_Freq = 500.15991521[MHz]
Irr_Offset = 5.0[ppm]
Tri_Domain = Proton
Tri_Freq = 500.15991521[MHz]
Tri_Offset = 5.0[ppm]
Clipped = FALSE
Scans = 40
Total_Scans = 40
Relaxation_Delay = 5[s]
Recvr_Gain = 50
Temp_Get = 18.4[dC]
X_90_Width = 16[us]
X_Acq_Time = 1.24780544[s]
X_Angle = 45[deg]
X_Atn = 2.5[dB]
X_Pulse = 8[us]
Irr_Mode = Off
Tri_Mode = Off
Dante_Presat = FALSE
Initial_Wait = 1[s]
Repetition_Time = 6.24780544[s]

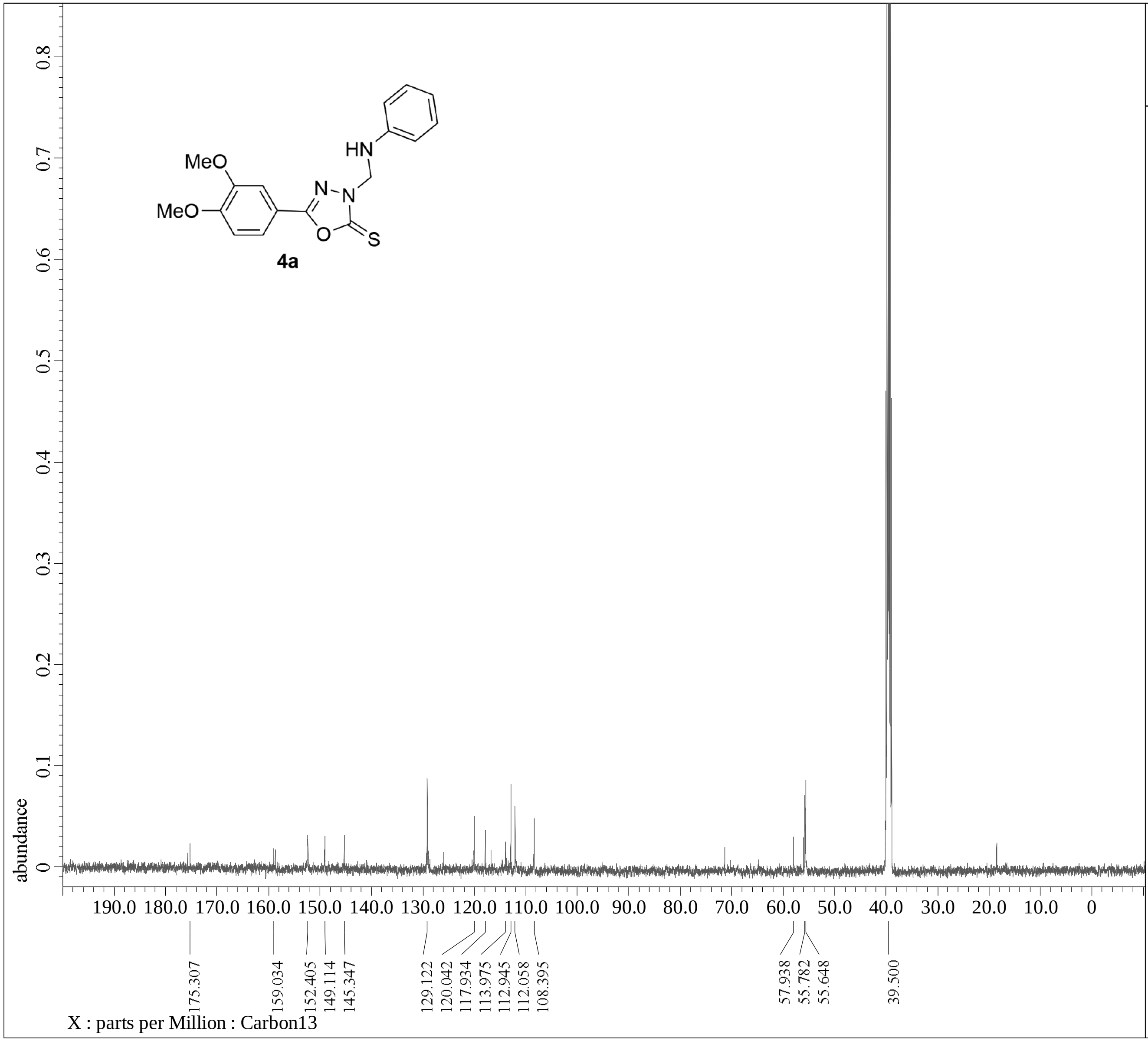


```
Filename          = Prof.DR Ali Elemam_DM_6_ca
Author            = delta
Experiment        = carbon.jxp
Sample_Id         = Prof.DR Ali Elemam_DM_6
Solvent           = DMSO-D6
Creation_Time     = 25-JAN-2021 19:15:38
Revision_Time     =  5-FEB-2021 07:54:05
Current_Time      =  5-FEB-2021 07:54:24

Comment           = single pulse decoupled gat
Data_Format       = 1D COMPLEX
Dim_Size          = 26214
Dim_Title         = Carbon13
Dim_Units         = [ppm]
Dimensions        = X
Site              = JNM-ECA500II
Spectrometer      = DELTA2_NMR

Field_Strength    = 11.7473579[T] (500[MHz])
X_Acq_Duration    = 0.83361792[s]
X_Domain          = 13C
X_Freq            = 125.76529768[MHz]
X_Offset          = 100[ppm]
X_Points          = 32768
X_Prescans        = 4
X_Resolution      = 1.19959034[Hz]
X_Sweep           = 39.3081761[kHz]
X_Sweep_Clipped   = 31.44654088[kHz]
Irr_Domain        = Proton
Irr_Freq          = 500.15991521[MHz]
Irr_Offset        = 5.0[ppm]
Clipped           = FALSE
Scans             = 1024
Total_Scans       = 1024

Relaxation_Delay  = 2[s]
Recvr_Gain        = 58
Temp_Get          = 19.9[dC]
X_90_Width        = 32.3[us]
X_Acq_Time        = 0.83361792[s]
X_Angle           = 30[deg]
X_Atn             = 10[dB]
X_Pulse           = 10.76666667[us]
Irr_Atn_Dec       = 17.693[dB]
Irr_Atn_Noe       = 17.693[dB]
Irr_Noise         = WALTZ
Irr_Pwidth        = 92[us]
Decoupling        = TRUE
Initial_Wait      = 1[s]
Noe               = TRUE
Noe_Time          = 2[s]
Repetition_Time   = 2.83361792[s]
```

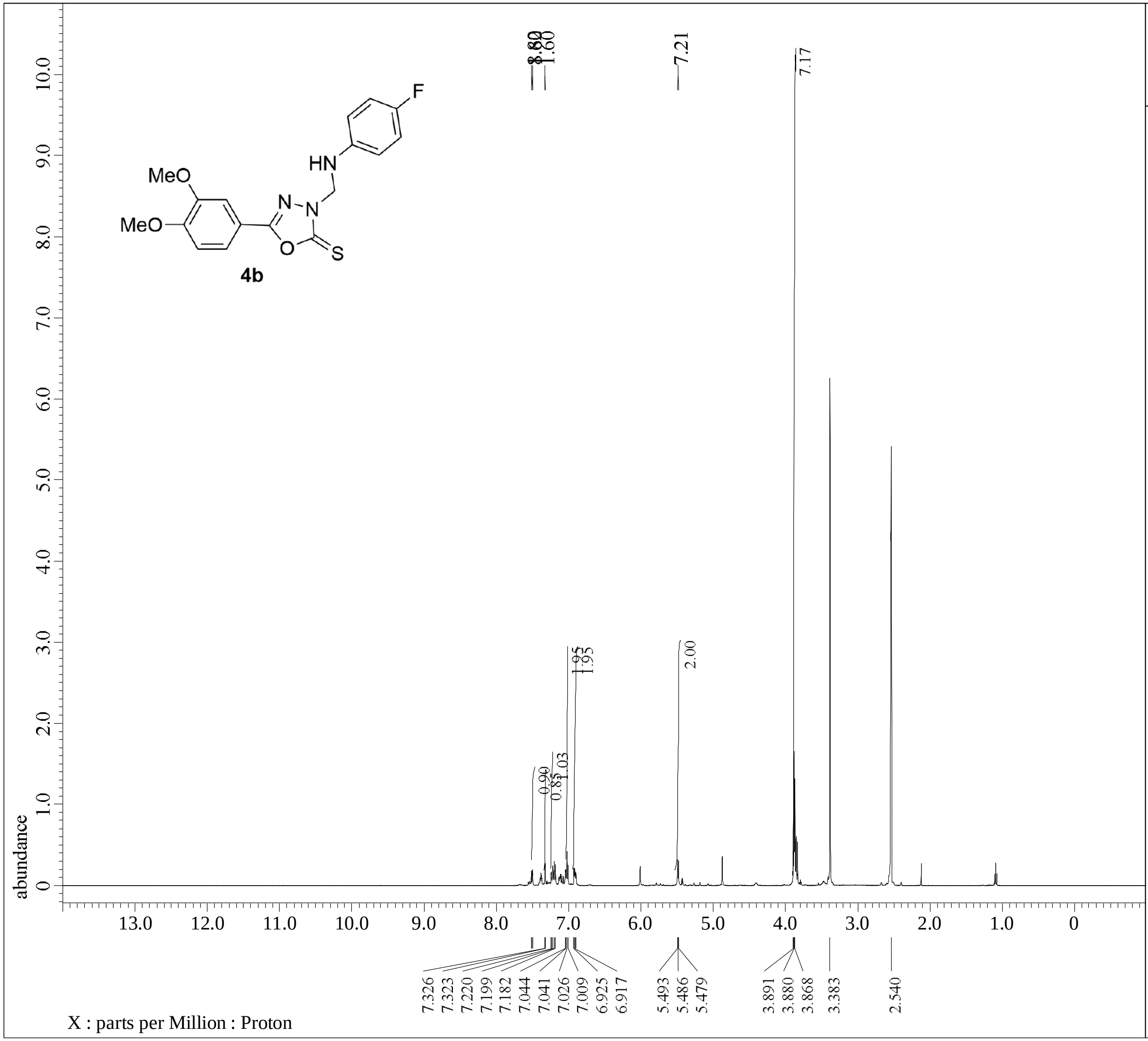


JEOL Solutions for Innovation

| | |
|---|---|
| Filename | = Prof.DR Ali Elemam_DM_7_pr |
| Author | = delta |
| Experiment | = proton.jxp |
| Sample_Id | = Prof.DR Ali Elemam_DM_7 |
| Solvent | = DMSO-D6 |
| Creation_Time | = 30-DEC-2020 11:45:19 |
| Revision_Time | = 6-FEB-2021 00:58:53 |
| Current_Time | = 6-FEB-2021 00:59:14 |
| Comment | = single_pulse |
| Data_Format | = 1D COMPLEX |
| Dim_Size | = 13107 |
| Dim_Title | = Proton |
| Dim_Units | = [ppm] |
| Dimensions | = X |
| Site | = JNM-ECA500II |
| Spectrometer | = DELTA2_NMR |
| Field_Strength | = 11.7473579[T] (500[MHz]) |
| X_Acq_Duration | = 1.24780544[s] |
| X_Domain | = 1H |
| X_Freq | = 500.15991521[MHz] |
| X_Offset | = 7.0[ppm] |
| X_Points | = 16384 |
| X_Prescans | = 1 |
| X_Resolution | = 0.80140699[Hz] |
| X_Sweep | = 13.1302521[kHz] |
| X_Sweep_Clipped | = 10.50420168[kHz] |
| Irr_Domain | = Proton |
| Irr_Freq | = 500.15991521[MHz] |
| Irr_Offset | = 5.0[ppm] |
| Tri_Domain | = Proton |
| Tri_Freq | = 500.15991521[MHz] |
| Tri_Offset | = 5.0[ppm] |
| Clipped | = FALSE |
| Scans | = 40 |
| Total_Scans | = 40 |
| Relaxation_Delay | = 5[s] |
| Recvr_Gain | = 50 |
| Temp_Get | = 18.1[dC] |
| X_90_Width | = 16[us] |
| X_Acq_Time | = 1.24780544[s] |
| X_Angle | = 45[deg] |
| X_Atn | = 2.5[dB] |
| X_Pulse | = 8[us] |
| Irr_Mode | = Off |
| Tri_Mode | = Off |
| Dante_Presat | = FALSE |
| Initial_Wait | = 1[s] |
| Repetition_Time | = 6.24780544[s] |

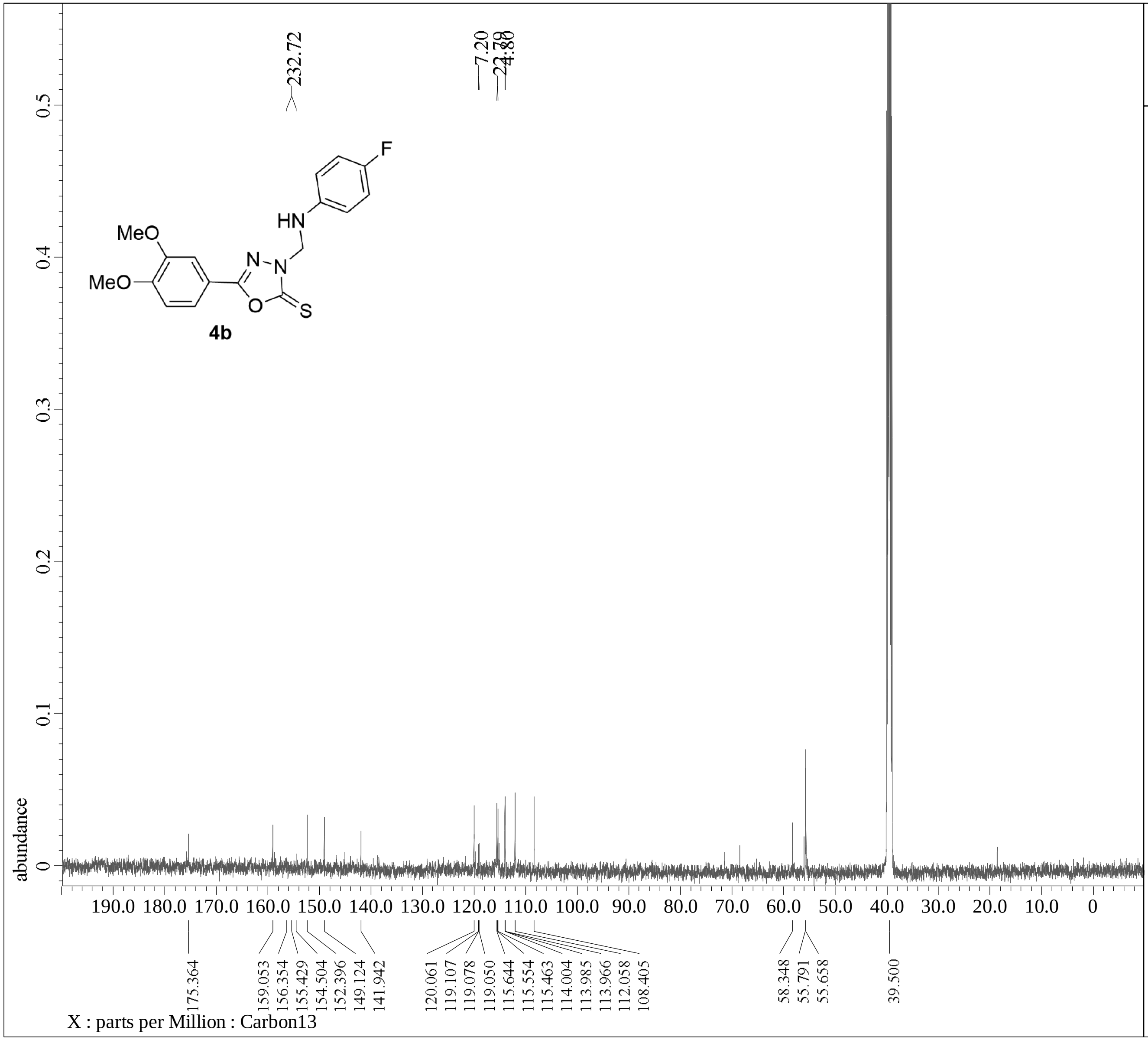


JEOL
Solutions for Innovation

Filename = Prof.DR Ali Elemam_DM_7_ca
Author = delta
Experiment = carbon.jxp
Sample_Id = Prof.DR Ali Elemam_DM_7
Solvent = DMSO-D6
Creation_Time = 25-JAN-2021 20:12:49
Revision_Time = 5-FEB-2021 08:13:23
Current_Time = 5-FEB-2021 08:13:58

Comment = single pulse decoupled gat
Data_Format = 1D COMPLEX
Dim_Size = 26214
Dim_Title = Carbon13
Dim_Units = [ppm]
Dimensions = X
Site = JNM-ECA500II
Spectrometer = DELTA2_NMR

Field_Strength = 11.7473579[T] (500[MHz])
X_Acq_Duration = 0.83361792[s]
X_Domain = 13C
X_Freq = 125.76529768[MHz]
X_Offset = 100[ppm]
X_Points = 32768
X_Prescans = 4
X_Resolution = 1.19959034[Hz]
X_Sweep = 39.3081761[kHz]
X_Sweep_Clipped = 31.44654088[kHz]
Irr_Domain = Proton
Irr_Freq = 500.15991521[MHz]
Irr_Offset = 5.0[ppm]
Clipped = FALSE
Scans = 1024
Total_Scans = 1024

Relaxation_Delay = 2[s]
Recvr_Gain = 58
Temp_Get = 20.2[dC]
X_90_Width = 32.3[us]
X_Acq_Time = 0.83361792[s]
X_Angle = 30[deg]
X_Atn = 10[dB]
X_Pulse = 10.76666667[us]
Irr_Atn_Dec = 17.693[dB]
Irr_Atn_Noe = 17.693[dB]
Irr_Noise = WALTZ
Irr_Pwidth = 92[us]
Decoupling = TRUE
Initial_Wait = 1[s]
Noe = TRUE
Noe_Time = 2[s]
Repetition_Time = 2.83361792[s]

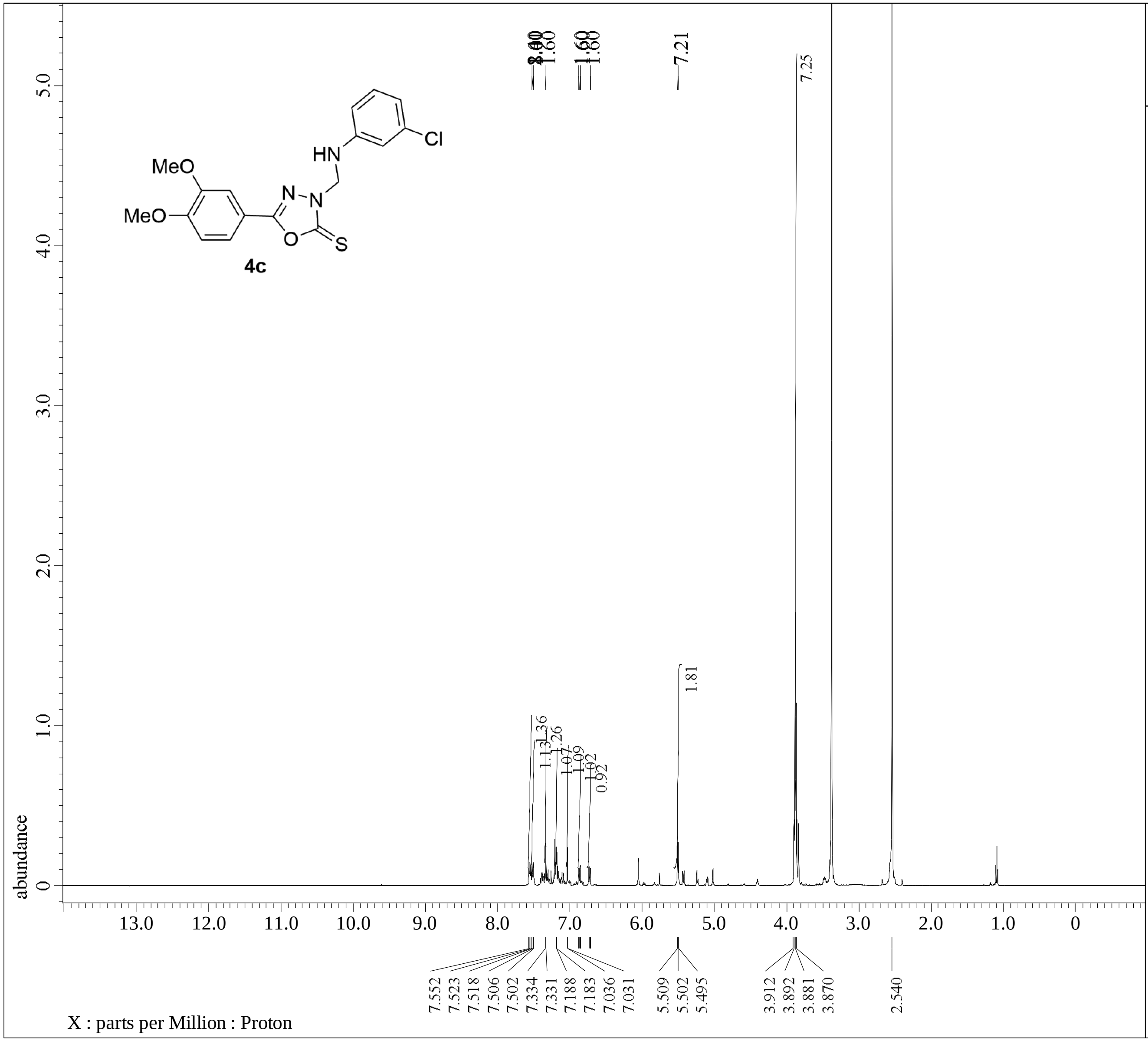


Solutions for Innovation

```
Filename          = Prof.DR Ali Elemam_DM_11_p
Author            = delta
Experiment        = proton.jxp
Sample_Id         = Prof.DR Ali Elemam_DM_11
Solvent           = DMSO-D6
Creation_Time     = 30-DEC-2020 14:12:33
Revision_Time     =  6-FEB-2021 09:12:15
Current_Time      =  6-FEB-2021 09:12:41

Comment           = single_pulse
Data_Format       = 1D COMPLEX
Dim_Size          = 13107
Dim_Title         = Proton
Dim_Units         = [ppm]
Dimensions        = X
Site              = JNM-ECA500II
Spectrometer      = DELTA2_NMR

Field_Strength    = 11.7473579[T] (500[MHz])
X_Acq_Duration    = 1.24780544[s]
X_Domain          = 1H
X_Freq            = 500.15991521[MHz]
X_Offset          = 7.0[ppm]
X_Points          = 16384
X_Prescans        = 1
X_Resolution      = 0.80140699[Hz]
X_Sweep           = 13.1302521[kHz]
X_Sweep_Clipped   = 10.50420168[kHz]
Irr_Domain        = Proton
Irr_Freq          = 500.15991521[MHz]
Irr_Offset        = 5.0[ppm]
Tri_Domain        = Proton
Tri_Freq          = 500.15991521[MHz]
Tri_Offset        = 5.0[ppm]
Clipped           = FALSE
Scans             = 40
Total_Scans       = 40

Relaxation_Delay  = 5[s]
Recvr_Gain        = 50
Temp_Get          = 18.8[dC]
X_90_Width        = 16[us]
X_Acq_Time        = 1.24780544[s]
X_Angle           = 45[deg]
X_Atn             = 2.5[dB]
X_Pulse           = 8[us]
Irr_Mode          = Off
Tri_Mode          = Off
Dante_Presat      = FALSE
Initial_Wait      = 1[s]
Repetition_Time   = 6.24780544[s]
```

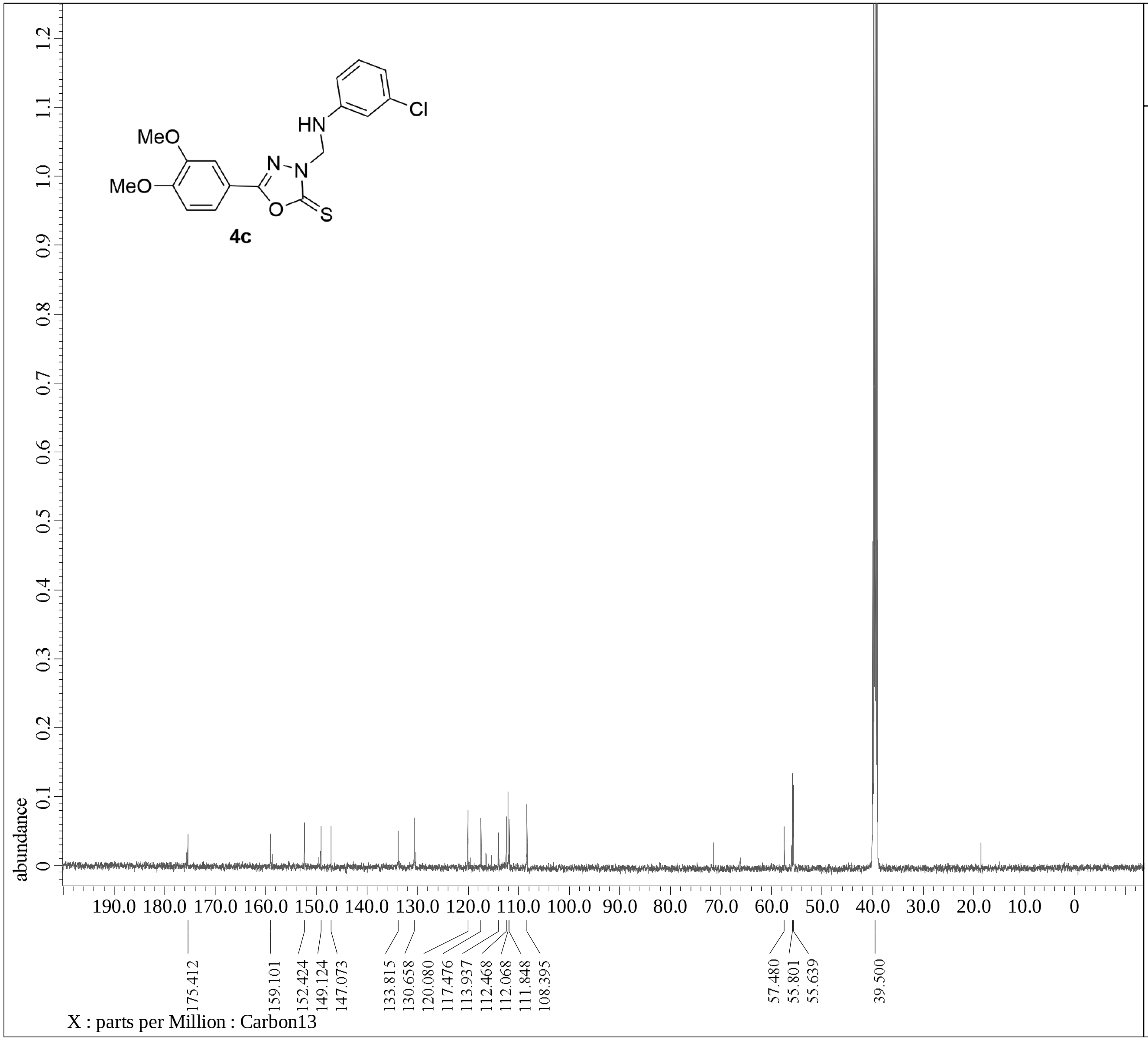


JEOL
Solutions for Innovation

```
Filename          = Prof.DR Ali Elemam_DM_11_c
Author            = delta
Experiment        = carbon.jxp
Sample_Id         = Prof.DR Ali Elemam_DM_11
Solvent           = DMSO-D6
Creation_Time     = 25-JAN-2021 22:07:07
Revision_Time     =  5-FEB-2021 04:17:14
Current_Time      =  5-FEB-2021 04:17:59

Comment           = single pulse decoupled gat
Data_Format       = 1D COMPLEX
Dim_Size          = 26214
Dim_Title         = Carbon13
Dim_Units         = [ppm]
Dimensions        = X
Site              = JNM-ECA500II
Spectrometer      = DELTA2_NMR

Field_Strength    = 11.7473579[T] (500[MHz])
X_Acq_Duration    = 0.83361792[s]
X_Domain          = 13C
X_Freq            = 125.76529768[MHz]
X_Offset          = 100[ppm]
X_Points          = 32768
X_Prescans        = 4
X_Resolution      = 1.19959034[Hz]
X_Sweep           = 39.3081761[kHz]
X_Sweep_Clipped   = 31.44654088[kHz]
Irr_Domain        = Proton
Irr_Freq          = 500.15991521[MHz]
Irr_Offset        = 5.0[ppm]
Clipped           = TRUE
Scans             = 1024
Total_Scans       = 1024

Relaxation_Delay  = 2[s]
Recvr_Gain        = 58
Temp_Get          = 20.3[dC]
X_90_Width        = 32.3[us]
X_Acq_Time        = 0.83361792[s]
X_Angle           = 30[deg]
X_Atn             = 10[dB]
X_Pulse           = 10.76666667[us]
Irr_Atn_Dec       = 17.693[dB]
Irr_Atn_Noe       = 17.693[dB]
Irr_Noise         = WALTZ
Irr_Pwidth        = 92[us]
Decoupling        = TRUE
Initial_Wait      = 1[s]
Noe               = TRUE
Noe_Time          = 2[s]
Repetition_Time   = 2.83361792[s]
```

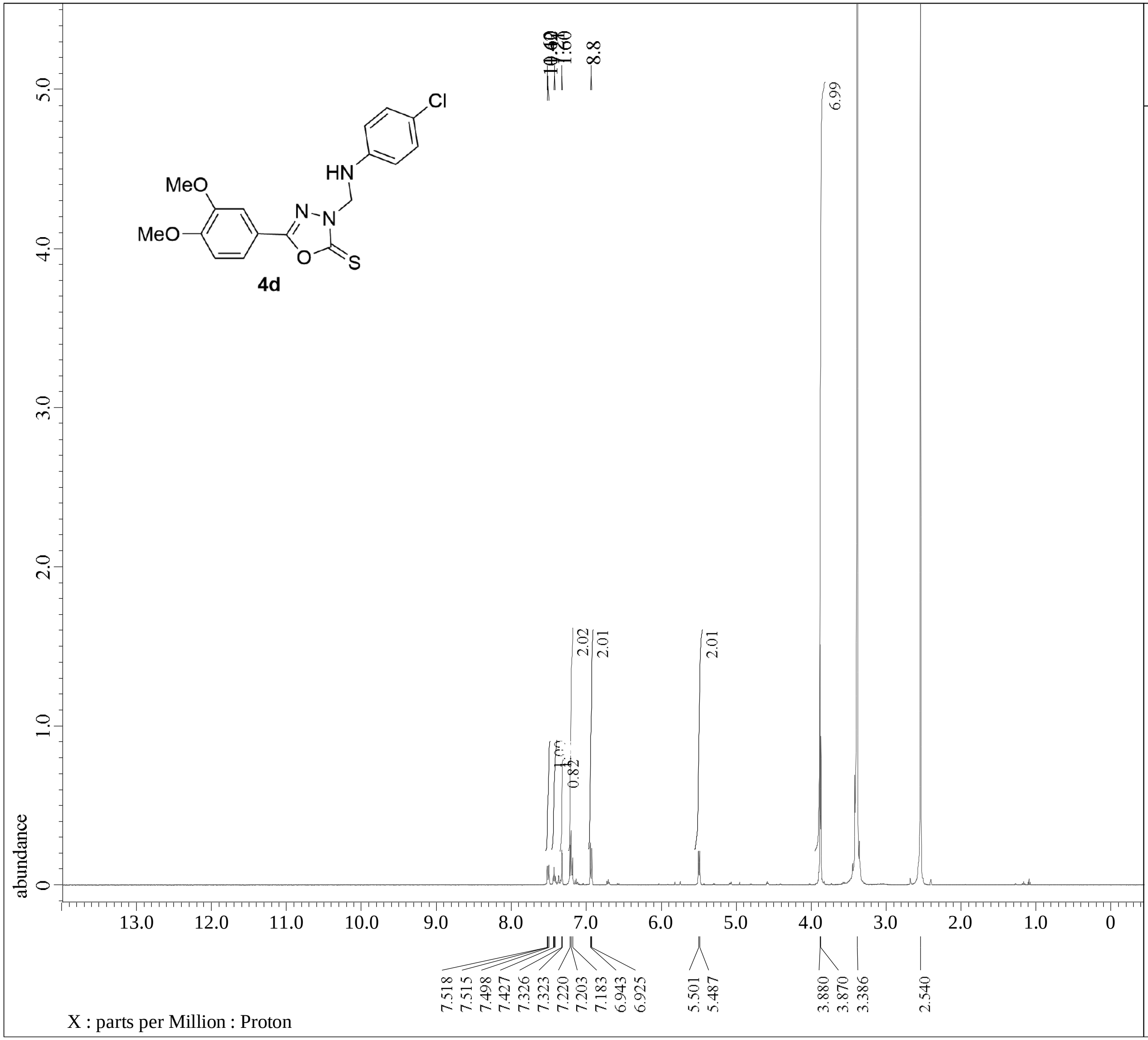


```
Filename          = Prof.DR Ali Elemam_DM_5_pr
Author            = delta
Experiment        = proton.jxp
Sample_Id         = Prof.DR Ali Elemam_DM_5
Solvent           = DMSO-D6
Creation_Time     = 14-DEC-2020 12:23:13
Revision_Time     =  5-FEB-2021 23:41:06
Current_Time      =  5-FEB-2021 23:41:30

Comment           = single_pulse
Data_Format       = 1D COMPLEX
Dim_Size          = 13107
Dim_Title         = Proton
Dim_Units         = [ppm]
Dimensions        = X
Site              = JNM-ECA500II
Spectrometer      = DELTA2_NMR

Field_Strength    = 11.7473579[T] (500[MHz])
X_Acq_Duration    = 1.24780544[s]
X_Domain          = 1H
X_Freq            = 500.15991521[MHz]
X_Offset          = 7.0[ppm]
X_Points          = 16384
X_Prescans        = 1
X_Resolution      = 0.80140699[Hz]
X_Sweep           = 13.1302521[kHz]
X_Sweep_Clipped   = 10.50420168[kHz]
Irr_Domain        = Proton
Irr_Freq          = 500.15991521[MHz]
Irr_Offset        = 5.0[ppm]
Tri_Domain        = Proton
Tri_Freq          = 500.15991521[MHz]
Tri_Offset        = 5.0[ppm]
Clipped           = FALSE
Scans             = 40
Total_Scans       = 40

Relaxation_Delay  = 5[s]
Recvr_Gain        = 50
Temp_Get          = 18.8[dC]
X_90_Width        = 16[us]
X_Acq_Time        = 1.24780544[s]
X_Angle           = 45[deg]
X_Atn             = 2.5[dB]
X_Pulse           = 8[us]
Irr_Mode          = Off
Tri_Mode          = Off
Dante_Presat      = FALSE
Initial_Wait      = 1[s]
Repetition_Time   = 6.24780544[s]
```

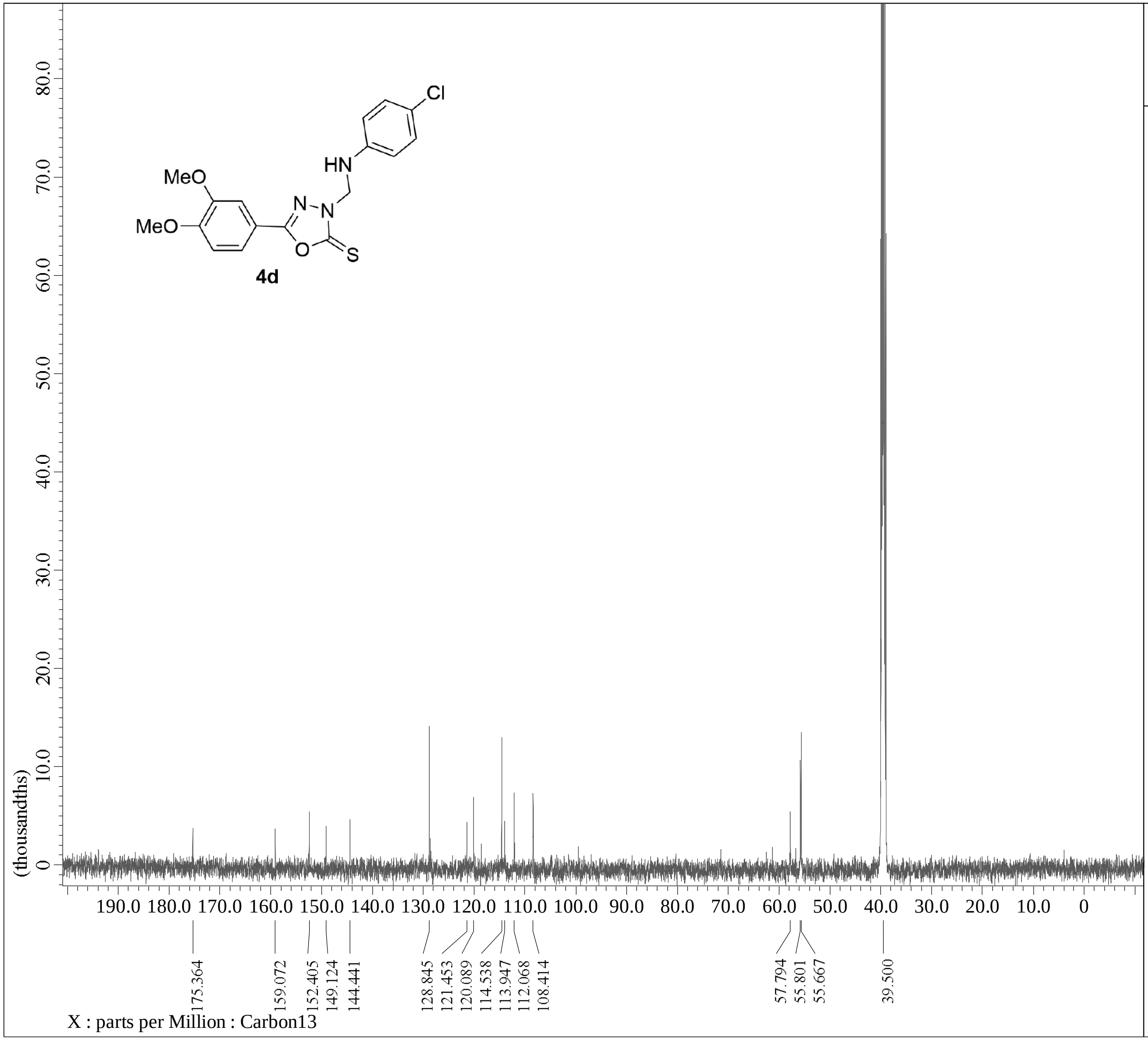


JEOL
Solutions for Innovation

```
Filename          = Prof.DR Ali Elemam_DM_5_ca
Author            = delta
Experiment        = carbon.jxp
Sample_Id         = Prof.DR Ali Elemam_DM_5
Solvent           = DMSO-D6
Creation_Time     = 22-DEC-2020 10:14:42
Revision_Time     =  5-FEB-2021 08:28:26
Current_Time      =  5-FEB-2021 08:29:17

Comment           = single pulse decoupled gat
Data_Format       = 1D COMPLEX
Dim_Size          = 26214
Dim_Title         = Carbon13
Dim_Units         = [ppm]
Dimensions        = X
Site              = JNM-ECA500II
Spectrometer      = DELTA2_NMR

Field_Strength    = 11.7473579[T] (500[MHz])
X_Acq_Duration    = 0.83361792[s]
X_Domain          = 13C
X_Freq            = 125.76529768[MHz]
X_Offset          = 100[ppm]
X_Points          = 32768
X_Prescans        = 4
X_Resolution      = 1.19959034[Hz]
X_Sweep           = 39.3081761[kHz]
X_Sweep_Clipped   = 31.44654088[kHz]
Irr_Domain        = Proton
Irr_Freq          = 500.15991521[MHz]
Irr_Offset        = 5.0[ppm]
Clipped           = FALSE
Scans             = 1500
Total_Scans       = 1500

Relaxation_Delay  = 2[s]
Recvr_Gain        = 50
Temp_Get          = 19.6[dC]
X_90_Width        = 32.3[us]
X_Acq_Time        = 0.83361792[s]
X_Angle           = 30[deg]
X_Atn             = 10[dB]
X_Pulse           = 10.76666667[us]
Irr_Atn_Dec       = 17.693[dB]
Irr_Atn_Noe       = 17.693[dB]
Irr_Noise         = WALTZ
Irr_Pwidth        = 92[us]
Decoupling        = TRUE
Initial_Wait      = 1[s]
Noe               = TRUE
Noe_Time          = 2[s]
Repetition_Time   = 2.83361792[s]
```

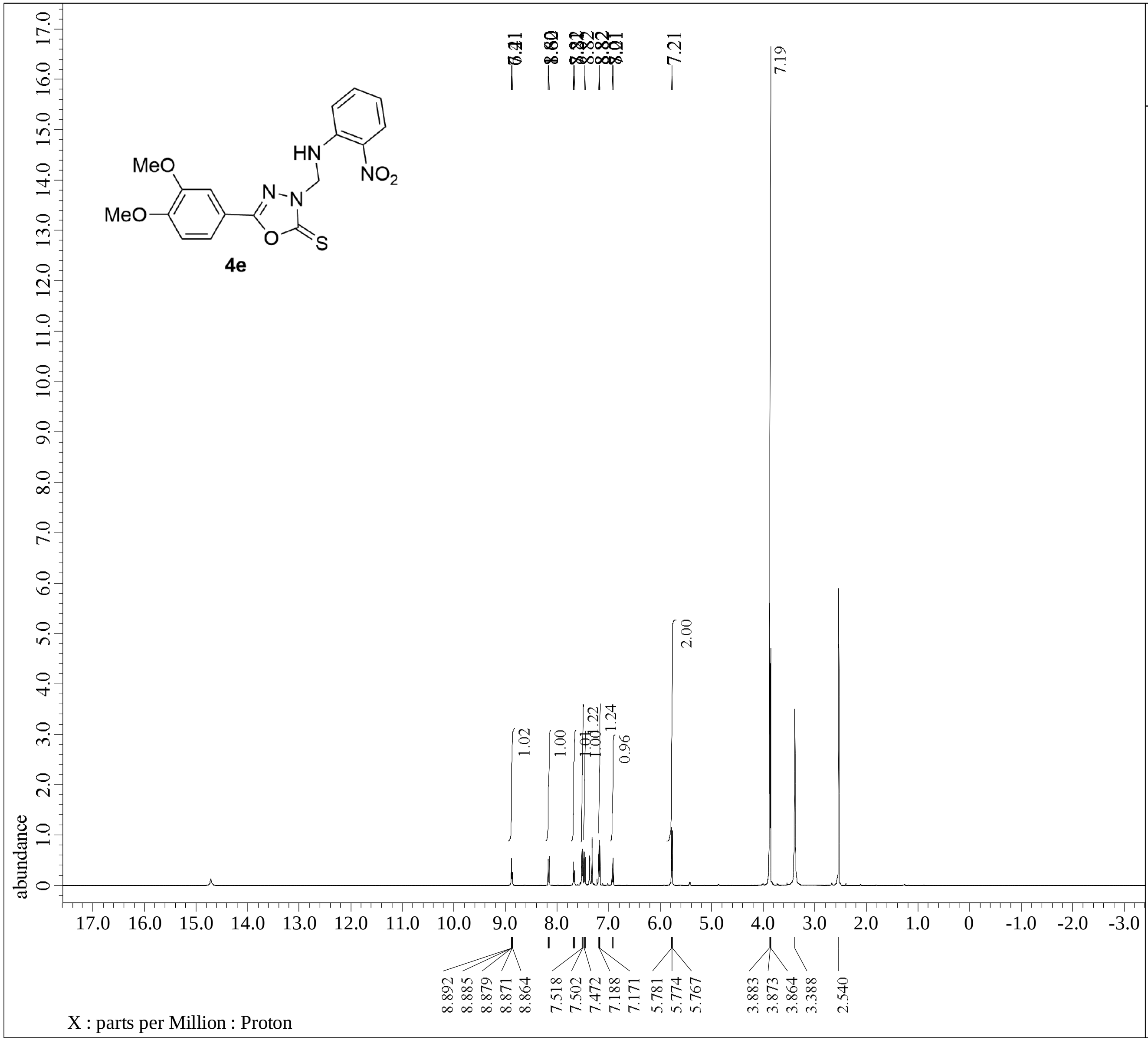


JEOL
Solutions for Innovation

```
Filename          = Prof.DR Ali Elemam_DM_8_pr
Author            = delta
Experiment        = proton.jxp
Sample_Id         = Prof.DR Ali Elemam_DM_8
Solvent           = DMSO-D6
Creation_Time     = 30-DEC-2020 11:54:18
Revision_Time     =  6-FEB-2021 01:29:21
Current_Time      =  6-FEB-2021 01:29:26

Comment           = single_pulse
Data_Format       = 1D COMPLEX
Dim_Size          = 13107
Dim_Title         = Proton
Dim_Units         = [ppm]
Dimensions        = X
Site              = JNM-ECA500II
Spectrometer      = DELTA2_NMR

Field_Strength    = 11.7473579[T] (500[MHz])
X_Acq_Duration    = 1.24780544[s]
X_Domain          = 1H
X_Freq            = 500.15991521[MHz]
X_Offset          = 7.0[ppm]
X_Points          = 16384
X_Prescans        = 1
X_Resolution      = 0.80140699[Hz]
X_Sweep           = 13.1302521[kHz]
X_Sweep_Clipped   = 10.50420168[kHz]
Irr_Domain        = Proton
Irr_Freq          = 500.15991521[MHz]
Irr_Offset        = 5.0[ppm]
Tri_Domain        = Proton
Tri_Freq          = 500.15991521[MHz]
Tri_Offset        = 5.0[ppm]
Clipped           = FALSE
Scans             = 40
Total_Scans       = 40

Relaxation_Delay  = 5[s]
Recvr_Gain        = 50
Temp_Get          = 18[dC]
X_90_Width        = 16[us]
X_Acq_Time        = 1.24780544[s]
X_Angle           = 45[deg]
X_Atn             = 2.5[dB]
X_Pulse           = 8[us]
Irr_Mode          = Off
Tri_Mode          = Off
Dante_Presat      = FALSE
Initial_Wait      = 1[s]
Repetition_Time   = 6.24780544[s]
```

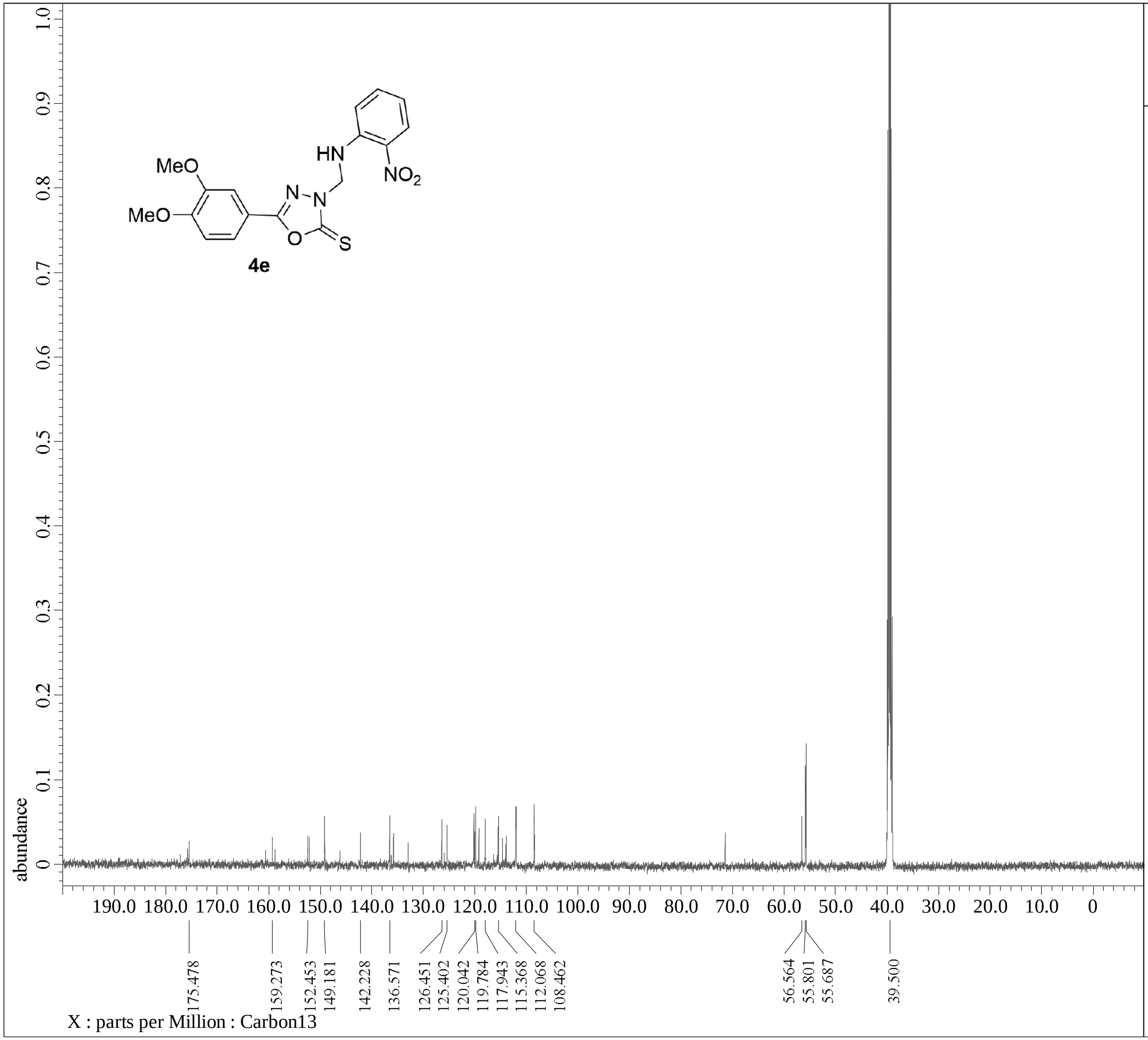


```
Filename         = Prof.DR Ali Elemam_DM_8_ca
Author           = delta
Experiment       = carbon.jxp
Sample_Id        = Prof.DR Ali Elemam_DM_8
Solvent          = DMSO-D6
Creation_Time    = 25-JAN-2021 17:23:30
Revision_Time    =  5-FEB-2021 08:20:39
Current_Time     =  5-FEB-2021 08:23:10

Comment          = single pulse decoupled gat
Data_Format      = 1D COMPLEX
Dim_Size         = 26214
Dim_Title        = Carbon13
Dim_Units        = [ppm]
Dimensions       = X
Site             = JNM-ECA500II
Spectrometer     = DELTA2_NMR

Field_Strength   = 11.7473579[T] (500[MHz])
X_Acq_Duration   = 0.83361792[s]
X_Domain         = 13C
X_Freq           = 125.76529768[MHz]
X_Offset         = 100[ppm]
X_Points         = 32768
X_Prescans       = 4
X_Resolution     = 1.19959034[Hz]
X_Sweep          = 39.3081761[kHz]
X_Sweep_Clipped  = 31.44654088[kHz]
Irr_Domain       = Proton
Irr_Freq         = 500.15991521[MHz]
Irr_Offset       = 5.0[ppm]
Clipped          = FALSE
Scans            = 1024
Total_Scans      = 1024

Relaxation_Delay = 2[s]
Recvr_Gain       = 58
Temp_Get         = 20.1[dC]
X_90_Width       = 32.3[us]
X_Acq_Time       = 0.83361792[s]
X_Angle          = 30[deg]
X_Atn            = 10[dB]
X_Pulse          = 10.76666667[us]
Irr_Atn_Dec      = 17.693[dB]
Irr_Atn_Noe      = 17.693[dB]
Irr_Noise        = WALTZ
Irr_Pwidth       = 92[us]
Decoupling       = TRUE
Initial_Wait     = 1[s]
Noe              = TRUE
Noe_Time         = 2[s]
Repetition_Time  = 2.83361792[s]
```

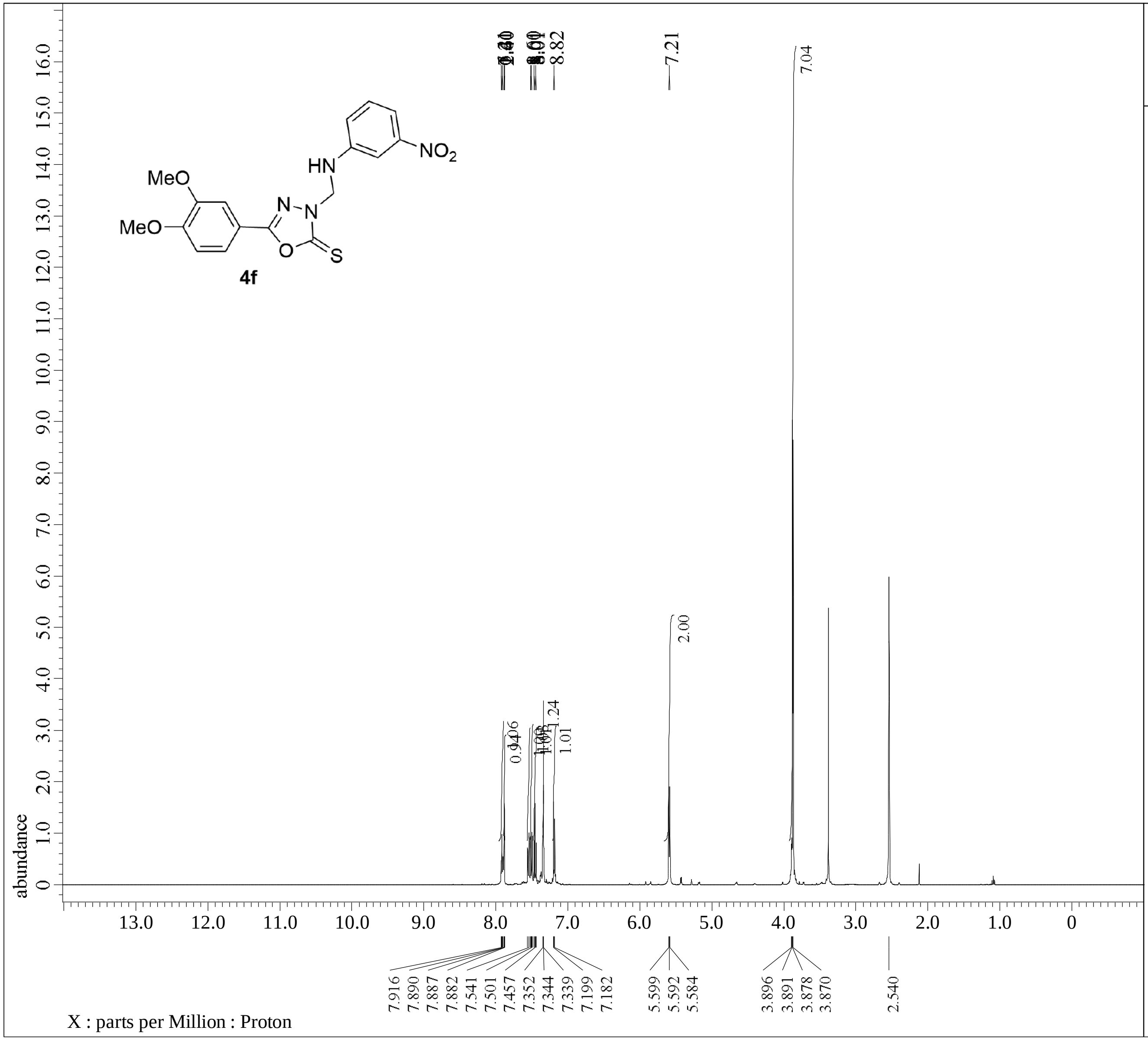


```
Filename          = Prof.DR Ali Elemam_DM_9_pr
Author            = delta
Experiment        = proton.jxp
Sample_Id         = Prof.DR Ali Elemam_DM_9
Solvent           = DMSO-D6
Creation_Time     = 30-DEC-2020 12:04:05
Revision_Time     =  6-FEB-2021 08:22:16
Current_Time      =  6-FEB-2021 08:23:07

Comment           = single_pulse
Data_Format       = 1D COMPLEX
Dim_Size          = 13107
Dim_Title         = Proton
Dim_Units         = [ppm]
Dimensions        = X
Site              = JNM-ECA500II
Spectrometer      = DELTA2_NMR

Field_Strength    = 11.7473579[T] (500[MHz])
X_Acq_Duration    = 1.24780544[s]
X_Domain          = 1H
X_Freq            = 500.15991521[MHz]
X_Offset          = 7.0[ppm]
X_Points          = 16384
X_Prescans        = 1
X_Resolution      = 0.80140699[Hz]
X_Sweep           = 13.1302521[kHz]
X_Sweep_Clipped   = 10.50420168[kHz]
Irr_Domain        = Proton
Irr_Freq          = 500.15991521[MHz]
Irr_Offset        = 5.0[ppm]
Tri_Domain        = Proton
Tri_Freq          = 500.15991521[MHz]
Tri_Offset        = 5.0[ppm]
Clipped           = FALSE
Scans             = 40
Total_Scans       = 40

Relaxation_Delay  = 5[s]
Recvr_Gain        = 50
Temp_Get          = 18.3[dC]
X_90_Width        = 16[us]
X_Acq_Time        = 1.24780544[s]
X_Angle           = 45[deg]
X_Atn             = 2.5[dB]
X_Pulse           = 8[us]
Irr_Mode          = Off
Tri_Mode          = Off
Dante_Presat      = FALSE
Initial_Wait      = 1[s]
Repetition_Time   = 6.24780544[s]
```

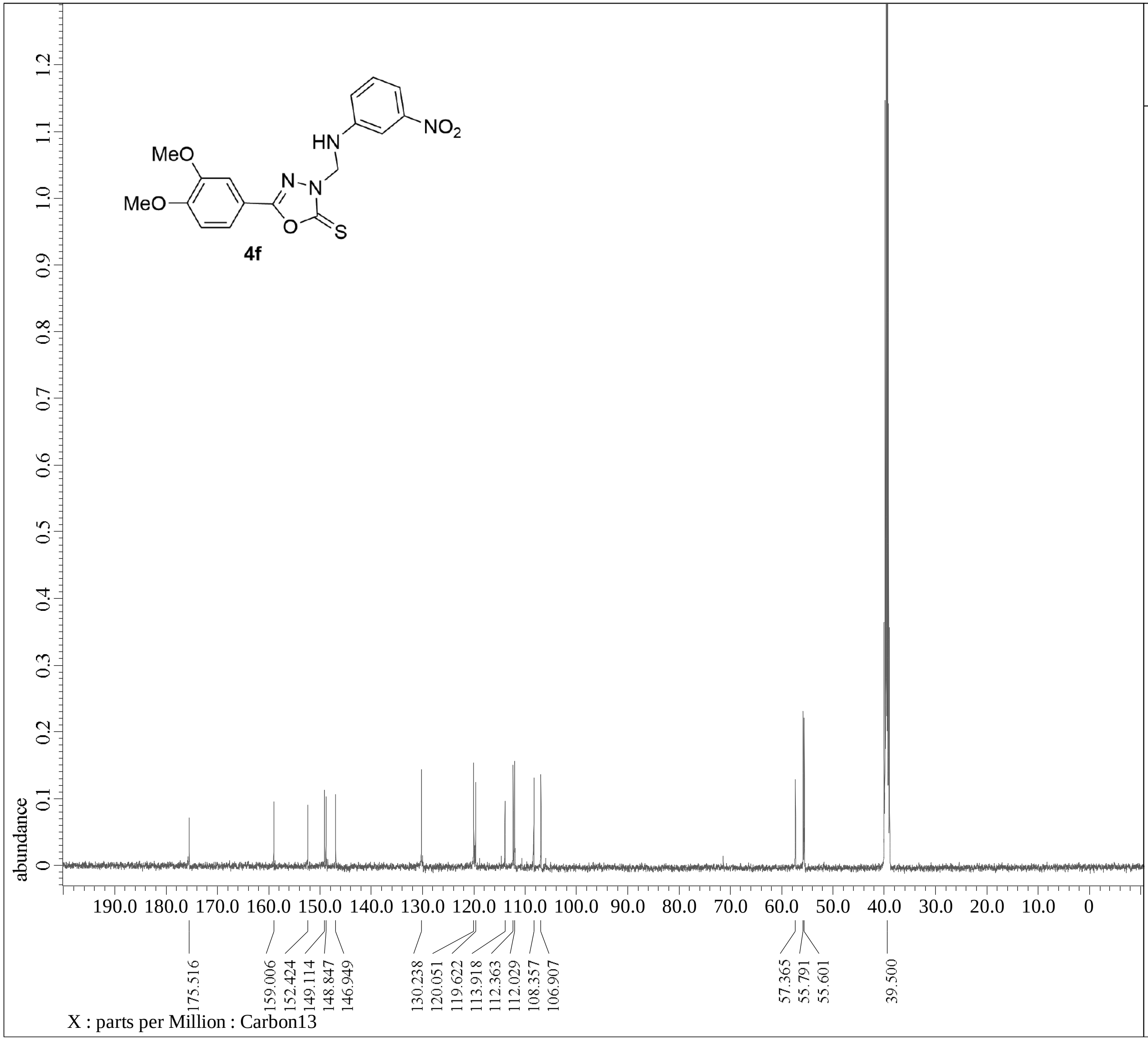


JEOL Solutions for Innovation

```
Filename           = Prof.DR Ali Elemam_DM_9_ca
Author             = delta
Experiment         = carbon.jxp
Sample_Id          = Prof.DR Ali Elemam_DM_9
Solvent            = DMSO-D6
Creation_Time      = 25-JAN-2021 18:19:54
Revision_Time      =  5-FEB-2021 03:37:02
Current_Time       =  5-FEB-2021 03:47:42

Comment            = single pulse decoupled gat
Data_Format        = 1D COMPLEX
Dim_Size           = 26214
Dim_Title          = Carbon13
Dim_Units          = [ppm]
Dimensions         = X
Site               = JNM-ECA500II
Spectrometer       = DELTA2_NMR

Field_Strength     = 11.7473579[T] (500[MHz])
X_Acq_Duration     = 0.83361792[s]
X_Domain           = 13C
X_Freq             = 125.76529768[MHz]
X_Offset           = 100[ppm]
X_Points           = 32768
X_Prescans         = 4
X_Resolution       = 1.19959034[Hz]
X_Sweep            = 39.3081761[kHz]
X_Sweep_Clipped    = 31.44654088[kHz]
Irr_Domain         = Proton
Irr_Freq           = 500.15991521[MHz]
Irr_Offset         = 5.0[ppm]
Clipped            = TRUE
Scans              = 1024
Total_Scans        = 1024

Relaxation_Delay   = 2[s]
Recvr_Gain         = 58
Temp_Get           = 19.6[dC]
X_90_Width         = 32.3[us]
X_Acq_Time         = 0.83361792[s]
X_Angle            = 30[deg]
X_Atn              = 10[dB]
X_Pulse            = 10.76666667[us]
Irr_Atn_Dec        = 17.693[dB]
Irr_Atn_Noe        = 17.693[dB]
Irr_Noise          = WALTZ
Irr_Pwidth         = 92[us]
Decoupling         = TRUE
Initial_Wait       = 1[s]
Noe                = TRUE
Noe_Time           = 2[s]
Repetition_Time    = 2.83361792[s]
```

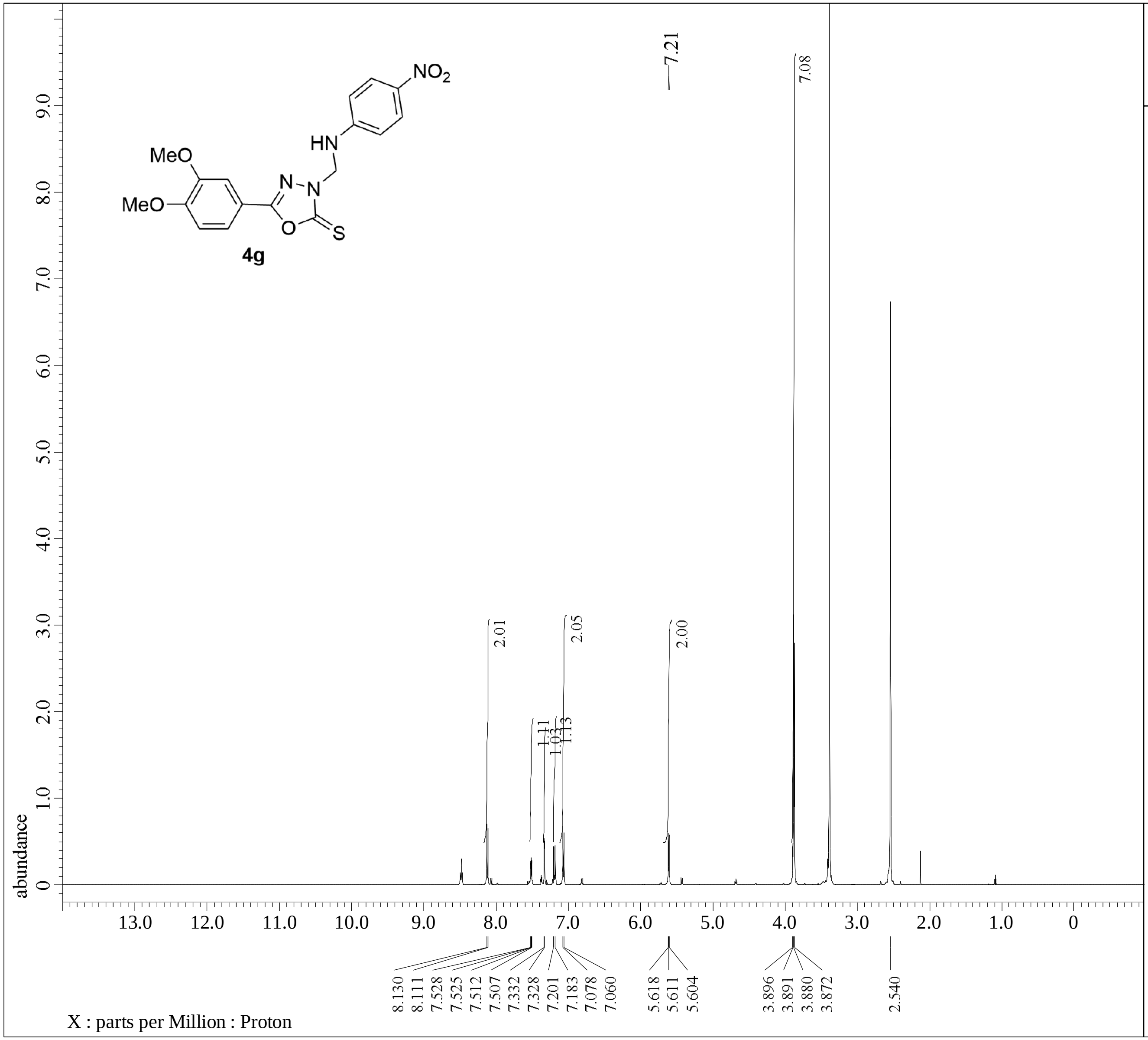


JEOL
Solutions for Innovation

```
Filename          = Prof.DR Ali Elemam_DM_10_p
Author            = delta
Experiment        = proton.jxp
Sample_Id         = Prof.DR Ali Elemam_DM_10
Solvent           = DMSO-D6
Creation_Time     = 30-DEC-2020 12:13:04
Revision_Time     =  6-FEB-2021 08:54:27
Current_Time      =  6-FEB-2021 08:55:41

Comment           = single_pulse
Data_Format       = 1D COMPLEX
Dim_Size          = 13107
Dim_Title         = Proton
Dim_Units         = [ppm]
Dimensions        = X
Site              = JNM-ECA500II
Spectrometer      = DELTA2_NMR

Field_Strength    = 11.7473579[T] (500[MHz])
X_Acq_Duration    = 1.24780544[s]
X_Domain          = 1H
X_Freq            = 500.15991521[MHz]
X_Offset          = 7.0[ppm]
X_Points          = 16384
X_Prescans        = 1
X_Resolution      = 0.80140699[Hz]
X_Sweep           = 13.1302521[kHz]
X_Sweep_Clipped   = 10.50420168[kHz]
Irr_Domain        = Proton
Irr_Freq          = 500.15991521[MHz]
Irr_Offset        = 5.0[ppm]
Tri_Domain        = Proton
Tri_Freq          = 500.15991521[MHz]
Tri_Offset        = 5.0[ppm]
Clipped           = FALSE
Scans             = 40
Total_Scans       = 40

Relaxation_Delay  = 5[s]
Recvr_Gain        = 50
Temp_Get          = 18.3[dC]
X_90_Width        = 16[us]
X_Acq_Time        = 1.24780544[s]
X_Angle           = 45[deg]
X_Atn             = 2.5[dB]
X_Pulse           = 8[us]
Irr_Mode          = Off
Tri_Mode          = Off
Dante_Presat      = FALSE
Initial_Wait      = 1[s]
Repetition_Time   = 6.24780544[s]
```

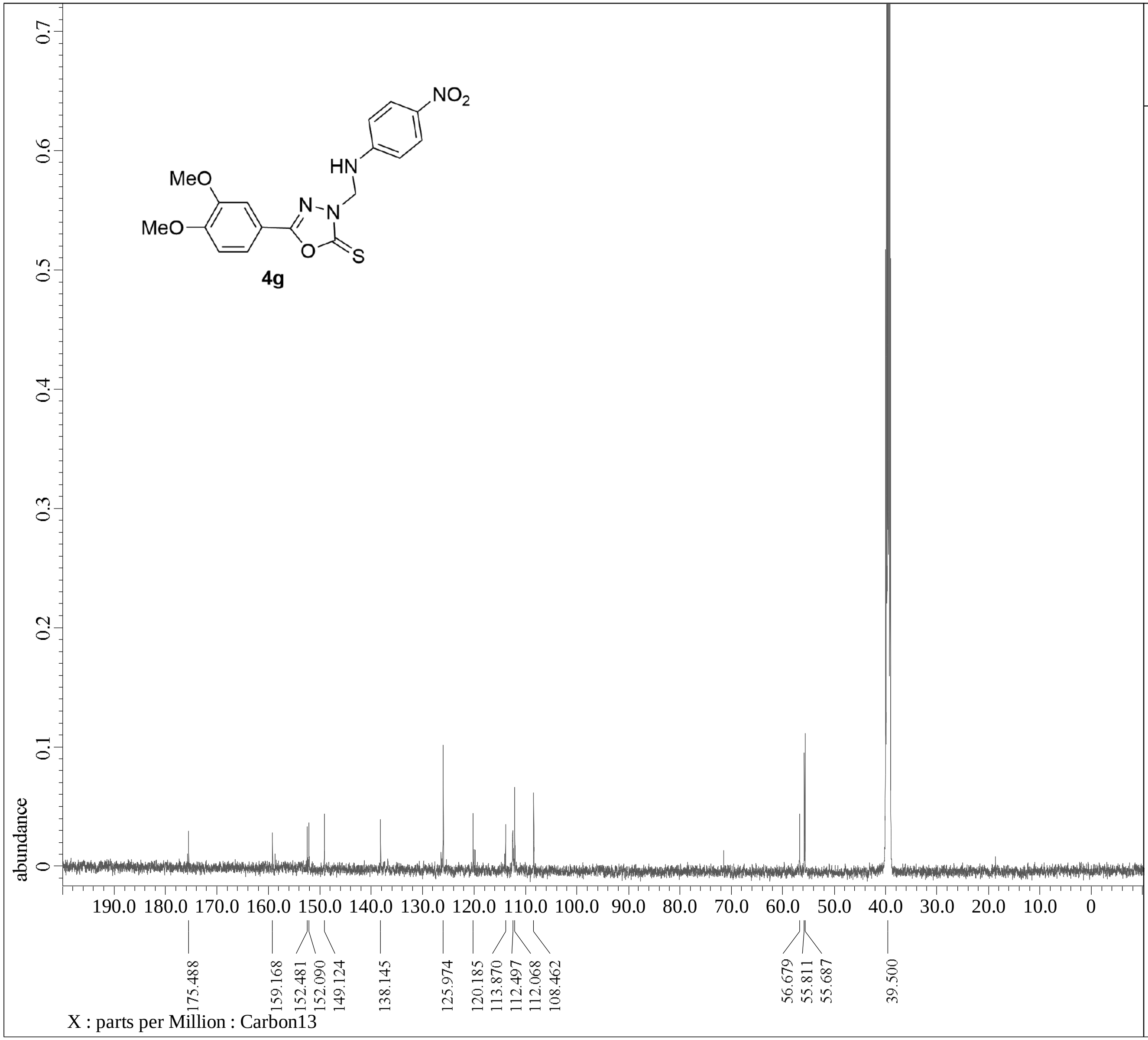


```
Filename          = Prof.DR Ali Elemam_DM_10_c
Author            = delta
Experiment        = carbon.jxp
Sample_Id         = Prof.DR Ali Elemam_DM_10
Solvent           = DMSO-D6
Creation_Time     = 25-JAN-2021 21:09:57
Revision_Time     =  5-FEB-2021 04:11:51
Current_Time      =  5-FEB-2021 04:13:33

Comment           = single pulse decoupled gat
Data_Format       = 1D COMPLEX
Dim_Size          = 26214
Dim_Title         = Carbon13
Dim_Units         = [ppm]
Dimensions        = X
Site              = JNM-ECA500II
Spectrometer      = DELTA2_NMR

Field_Strength    = 11.7473579[T] (500[MHz])
X_Acq_Duration    = 0.83361792[s]
X_Domain          = 13C
X_Freq            = 125.76529768[MHz]
X_Offset          = 100[ppm]
X_Points          = 32768
X_Prescans        = 4
X_Resolution      = 1.19959034[Hz]
X_Sweep           = 39.3081761[kHz]
X_Sweep_Clipped   = 31.44654088[kHz]
Irr_Domain        = Proton
Irr_Freq          = 500.15991521[MHz]
Irr_Offset        = 5.0[ppm]
Clipped           = FALSE
Scans             = 1024
Total_Scans       = 1024

Relaxation_Delay  = 2[s]
Recvr_Gain        = 58
Temp_Get          = 19.7[dC]
X_90_Width        = 32.3[us]
X_Acq_Time        = 0.83361792[s]
X_Angle           = 30[deg]
X_Atn             = 10[dB]
X_Pulse           = 10.76666667[us]
Irr_Atn_Dec       = 17.693[dB]
Irr_Atn_Noe       = 17.693[dB]
Irr_Noise         = WALTZ
Irr_Pwidth        = 92[us]
Decoupling        = TRUE
Initial_Wait      = 1[s]
Noe               = TRUE
Noe_Time          = 2[s]
Repetition_Time   = 2.83361792[s]
```

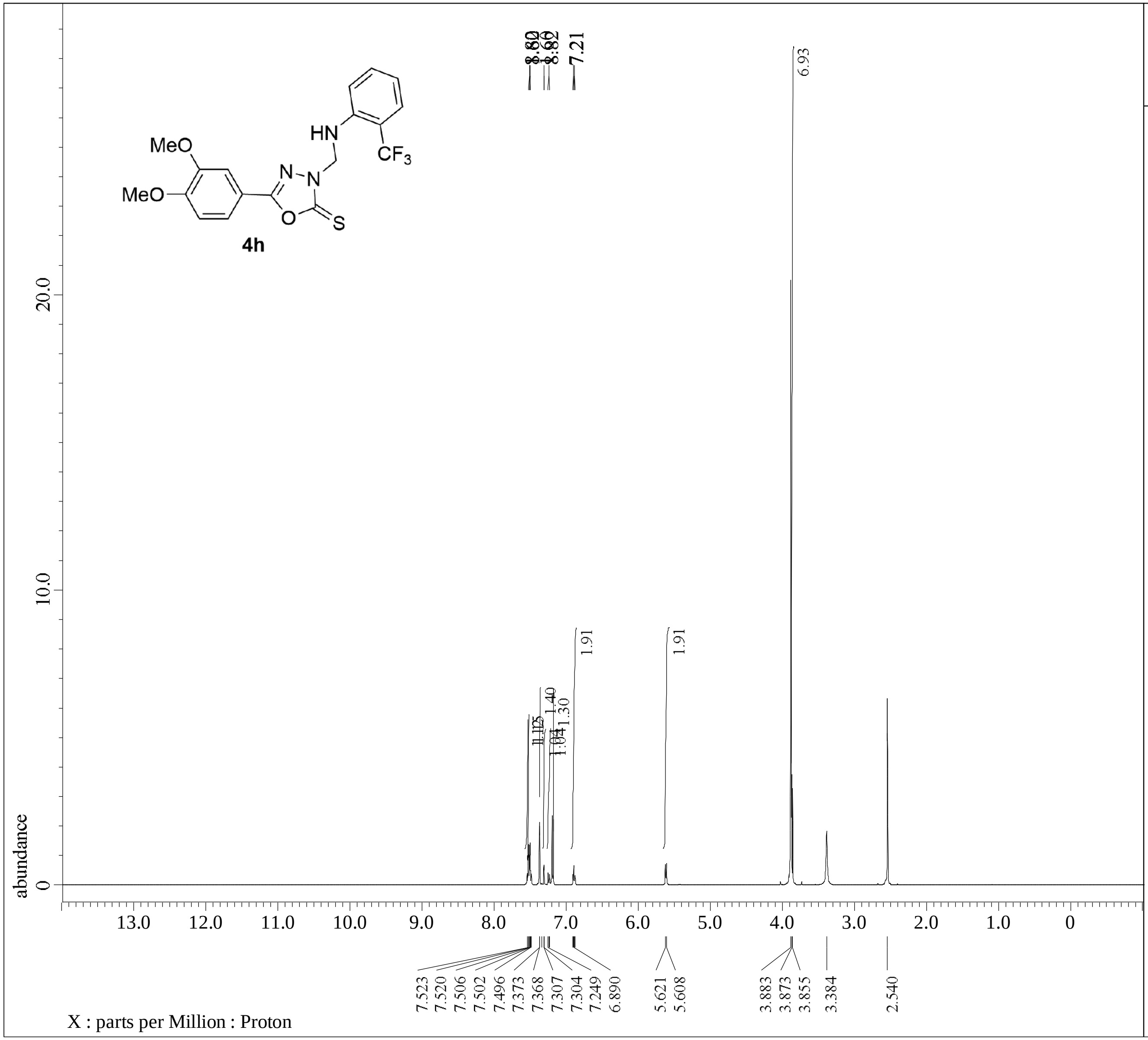


JEOL
Solutions for Innovation

```
Filename          = Prof.DR Ali Elemam_DM_12_p
Author            = delta
Experiment        = proton.jxp
Sample_Id         = Prof.DR Ali Elemam_DM_12
Solvent           = DMSO-D6
Creation_Time     = 30-DEC-2020 14:22:18
Revision_Time     =  6-FEB-2021 09:38:13
Current_Time      =  6-FEB-2021 09:38:30

Comment           = single_pulse
Data_Format       = 1D COMPLEX
Dim_Size          = 13107
Dim_Title         = Proton
Dim_Units         = [ppm]
Dimensions        = X
Site              = JNM-ECA500II
Spectrometer      = DELTA2_NMR

Field_Strength    = 11.7473579[T] (500[MHz])
X_Acq_Duration    = 1.24780544[s]
X_Domain          = 1H
X_Freq            = 500.15991521[MHz]
X_Offset          = 7.0[ppm]
X_Points          = 16384
X_Prescans        = 1
X_Resolution      = 0.80140699[Hz]
X_Sweep           = 13.1302521[kHz]
X_Sweep_Clipped   = 10.50420168[kHz]
Irr_Domain        = Proton
Irr_Freq          = 500.15991521[MHz]
Irr_Offset        = 5.0[ppm]
Tri_Domain        = Proton
Tri_Freq          = 500.15991521[MHz]
Tri_Offset        = 5.0[ppm]
Clipped           = FALSE
Scans             = 40
Total_Scans       = 40

Relaxation_Delay  = 5[s]
Recvr_Gain        = 50
Temp_Get          = 19.2[dC]
X_90_Width        = 16[us]
X_Acq_Time        = 1.24780544[s]
X_Angle           = 45[deg]
X_Atn             = 2.5[dB]
X_Pulse           = 8[us]
Irr_Mode          = Off
Tri_Mode          = Off
Dante_Presat      = FALSE
Initial_Wait      = 1[s]
Repetition_Time   = 6.24780544[s]
```

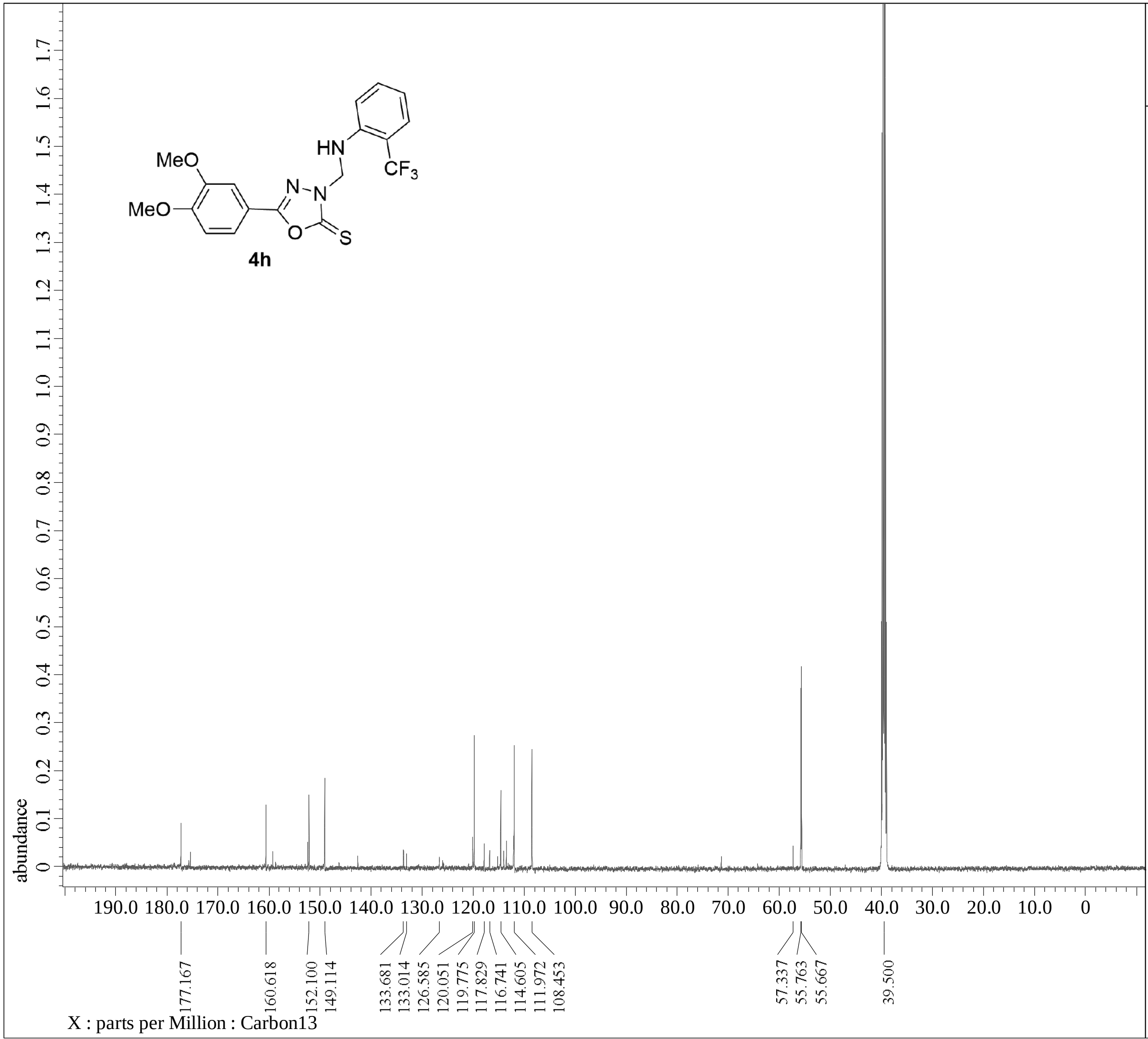


JEOL
Solutions for Innovation

```
Filename          = Prof.DR Ali Elemam_DM_12_c
Author            = delta
Experiment        = carbon.jxp
Sample_Id         = Prof.DR Ali Elemam_DM_12
Solvent           = DMSO-D6
Creation_Time     = 25-JAN-2021 23:04:23
Revision_Time     =  5-FEB-2021 04:27:37
Current_Time      =  5-FEB-2021 04:30:28

Comment           = single pulse decoupled gat
Data_Format       = 1D COMPLEX
Dim_Size          = 26214
Dim_Title         = Carbon13
Dim_Units         = [ppm]
Dimensions        = X
Site              = JNM-ECA500II
Spectrometer      = DELTA2_NMR

Field_Strength    = 11.7473579[T] (500[MHz])
X_Acq_Duration    = 0.83361792[s]
X_Domain          = 13C
X_Freq            = 125.76529768[MHz]
X_Offset          = 100[ppm]
X_Points          = 32768
X_Prescans        = 4
X_Resolution      = 1.19959034[Hz]
X_Sweep           = 39.3081761[kHz]
X_Sweep_Clipped   = 31.44654088[kHz]
Irr_Domain        = Proton
Irr_Freq          = 500.15991521[MHz]
Irr_Offset        = 5.0[ppm]
Clipped           = FALSE
Scans             = 1024
Total_Scans       = 1024

Relaxation_Delay  = 2[s]
Recvr_Gain        = 58
Temp_Get          = 19.6[dC]
X_90_Width        = 32.3[us]
X_Acq_Time        = 0.83361792[s]
X_Angle           = 30[deg]
X_Atn             = 10[dB]
X_Pulse           = 10.76666667[us]
Irr_Atn_Dec       = 17.693[dB]
Irr_Atn_Noe       = 17.693[dB]
Irr_Noise         = WALTZ
Irr_Pwidth        = 92[us]
Decoupling        = TRUE
Initial_Wait      = 1[s]
Noe               = TRUE
Noe_Time          = 2[s]
Repetition_Time   = 2.83361792[s]
```

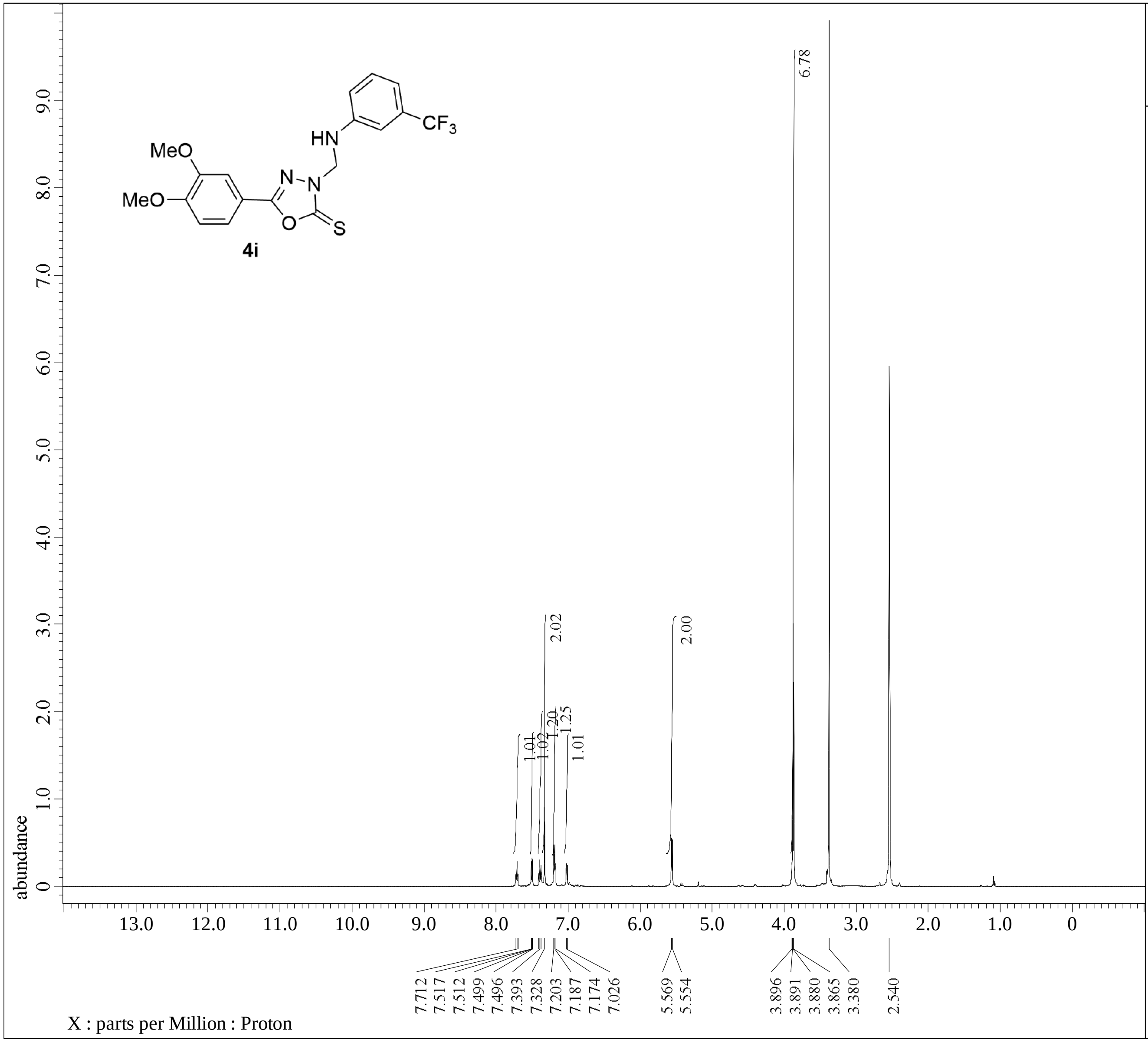


| | |
|---|---|
| Filename | = Prof.DR Ali Elemam_DM_13_p |
| Author | = delta |
| Experiment | = proton.jxp |
| Sample_Id | = Prof.DR Ali Elemam_DM_13 |
| Solvent | = DMSO-D6 |
| Creation_Time | = 30-DEC-2020 14:31:19 |
| Revision_Time | = 6-FEB-2021 09:42:39 |
| Current_Time | = 6-FEB-2021 09:44:24 |
| Comment | = single_pulse |
| Data_Format | = 1D COMPLEX |
| Dim_Size | = 13107 |
| Dim_Title | = Proton |
| Dim_Units | = [ppm] |
| Dimensions | = X |
| Site | = JNM-ECA500II |
| Spectrometer | = DELTA2_NMR |
| Field_Strength | = 11.7473579[T] (500[MHz]) |
| X_Acq_Duration | = 1.24780544[s] |
| X_Domain | = 1H |
| X_Freq | = 500.15991521[MHz] |
| X_Offset | = 7.0[ppm] |
| X_Points | = 16384 |
| X_Prescans | = 1 |
| X_Resolution | = 0.80140699[Hz] |
| X_Sweep | = 13.1302521[kHz] |
| X_Sweep_Clipped | = 10.50420168[kHz] |
| Irr_Domain | = Proton |
| Irr_Freq | = 500.15991521[MHz] |
| Irr_Offset | = 5.0[ppm] |
| Tri_Domain | = Proton |
| Tri_Freq | = 500.15991521[MHz] |
| Tri_Offset | = 5.0[ppm] |
| Clipped | = FALSE |
| Scans | = 40 |
| Total_Scans | = 40 |
| Relaxation_Delay | = 5[s] |
| Recvr_Gain | = 50 |
| Temp_Get | = 19.2[dC] |
| X_90_Width | = 16[us] |
| X_Acq_Time | = 1.24780544[s] |
| X_Angle | = 45[deg] |
| X_Atn | = 2.5[dB] |
| X_Pulse | = 8[us] |
| Irr_Mode | = Off |
| Tri_Mode | = Off |
| Dante_Presat | = FALSE |
| Initial_Wait | = 1[s] |
| Repetition_Time | = 6.24780544[s] |

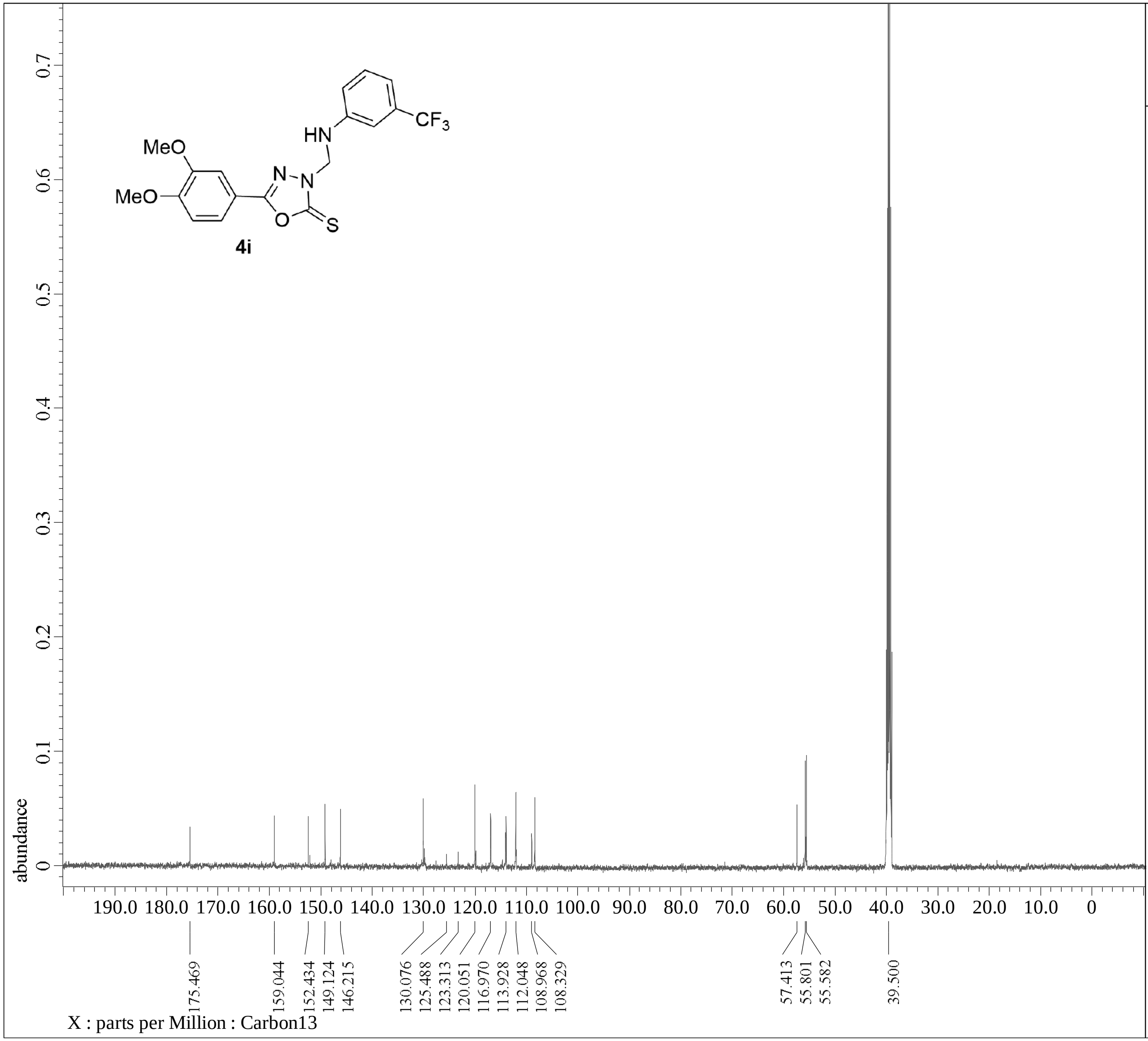


JEOL
Solutions for Innovation

```
Filename          = Prof.DR Ali Elemam_DM_13_c
Author            = delta
Experiment        = carbon.jxp
Sample_Id         = Prof.DR Ali Elemam_DM_13
Solvent           = DMSO-D6
Creation_Time     =  3-FEB-2021 20:42:59
Revision_Time     =  5-FEB-2021 04:40:24
Current_Time      =  5-FEB-2021 04:40:50

Comment           = single pulse decoupled gat
Data_Format       = 1D COMPLEX
Dim_Size          = 26214
Dim_Title         = Carbon13
Dim_Units         = [ppm]
Dimensions        = X
Site              = JNM-ECA500II
Spectrometer      = DELTA2_NMR

Field_Strength    = 11.7473579[T] (500[MHz])
X_Acq_Duration    = 0.83361792[s]
X_Domain          = 13C
X_Freq            = 125.76529768[MHz]
X_Offset          = 100[ppm]
X_Points          = 32768
X_Prescans        = 4
X_Resolution      = 1.19959034[Hz]
X_Sweep           = 39.3081761[kHz]
X_Sweep_Clipped   = 31.44654088[kHz]
Irr_Domain        = Proton
Irr_Freq          = 500.15991521[MHz]
Irr_Offset        = 5.0[ppm]
Clipped           = FALSE
Scans             = 1850
Total_Scans       = 1850

Relaxation_Delay  = 2[s]
Recvr_Gain        = 60
Temp_Get          = 20.4[dC]
X_90_Width        = 32.3[us]
X_Acq_Time        = 0.83361792[s]
X_Angle           = 30[deg]
X_Atn             = 10[dB]
X_Pulse           = 10.76666667[us]
Irr_Atn_Dec       = 17.693[dB]
Irr_Atn_Noe       = 17.693[dB]
Irr_Noise         = WALTZ
Irr_Pwidth        = 92[us]
Decoupling        = TRUE
Initial_Wait      = 1[s]
Noe               = TRUE
Noe_Time          = 2[s]
Repetition_Time   = 2.83361792[s]
```

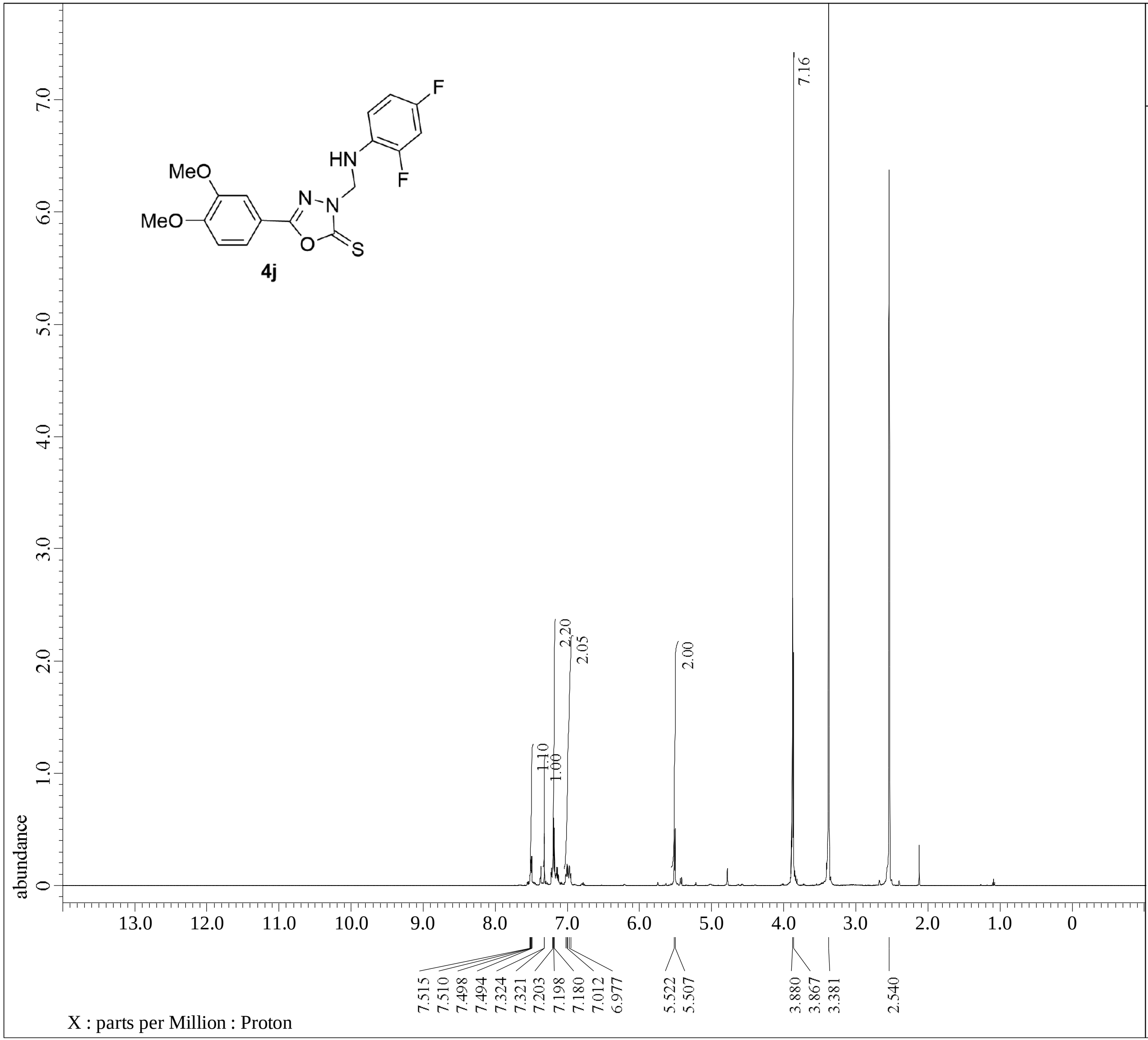


```
Filename         = Prof.DR Ali Elemam_DM_15_p
Author           = delta
Experiment       = proton.jxp
Sample_Id        = Prof.DR Ali Elemam_DM_15
Solvent          = DMSO-D6
Creation_Time    = 30-DEC-2020 13:00:20
Revision_Time    =  6-FEB-2021 10:22:51
Current_Time     =  6-FEB-2021 10:24:11

Comment          = single_pulse
Data_Format      = 1D COMPLEX
Dim_Size         = 13107
Dim_Title        = Proton
Dim_Units        = [ppm]
Dimensions       = X
Site             = JNM-ECA500II
Spectrometer     = DELTA2_NMR

Field_Strength   = 11.7473579[T] (500[MHz])
X_Acq_Duration   = 1.24780544[s]
X_Domain         = 1H
X_Freq           = 500.15991521[MHz]
X_Offset         = 7.0[ppm]
X_Points         = 16384
X_Prescans       = 1
X_Resolution     = 0.80140699[Hz]
X_Sweep          = 13.1302521[kHz]
X_Sweep_Clipped  = 10.50420168[kHz]
Irr_Domain       = Proton
Irr_Freq         = 500.15991521[MHz]
Irr_Offset       = 5.0[ppm]
Tri_Domain       = Proton
Tri_Freq         = 500.15991521[MHz]
Tri_Offset       = 5.0[ppm]
Clipped          = FALSE
Scans            = 40
Total_Scans      = 40

Relaxation_Delay = 5[s]
Recvr_Gain       = 50
Temp_Get         = 18.4[dC]
X_90_Width       = 16[us]
X_Acq_Time       = 1.24780544[s]
X_Angle          = 45[deg]
X_Atn            = 2.5[dB]
X_Pulse          = 8[us]
Irr_Mode         = Off
Tri_Mode         = Off
Dante_Presat     = FALSE
Initial_Wait     = 1[s]
Repetition_Time  = 6.24780544[s]
```

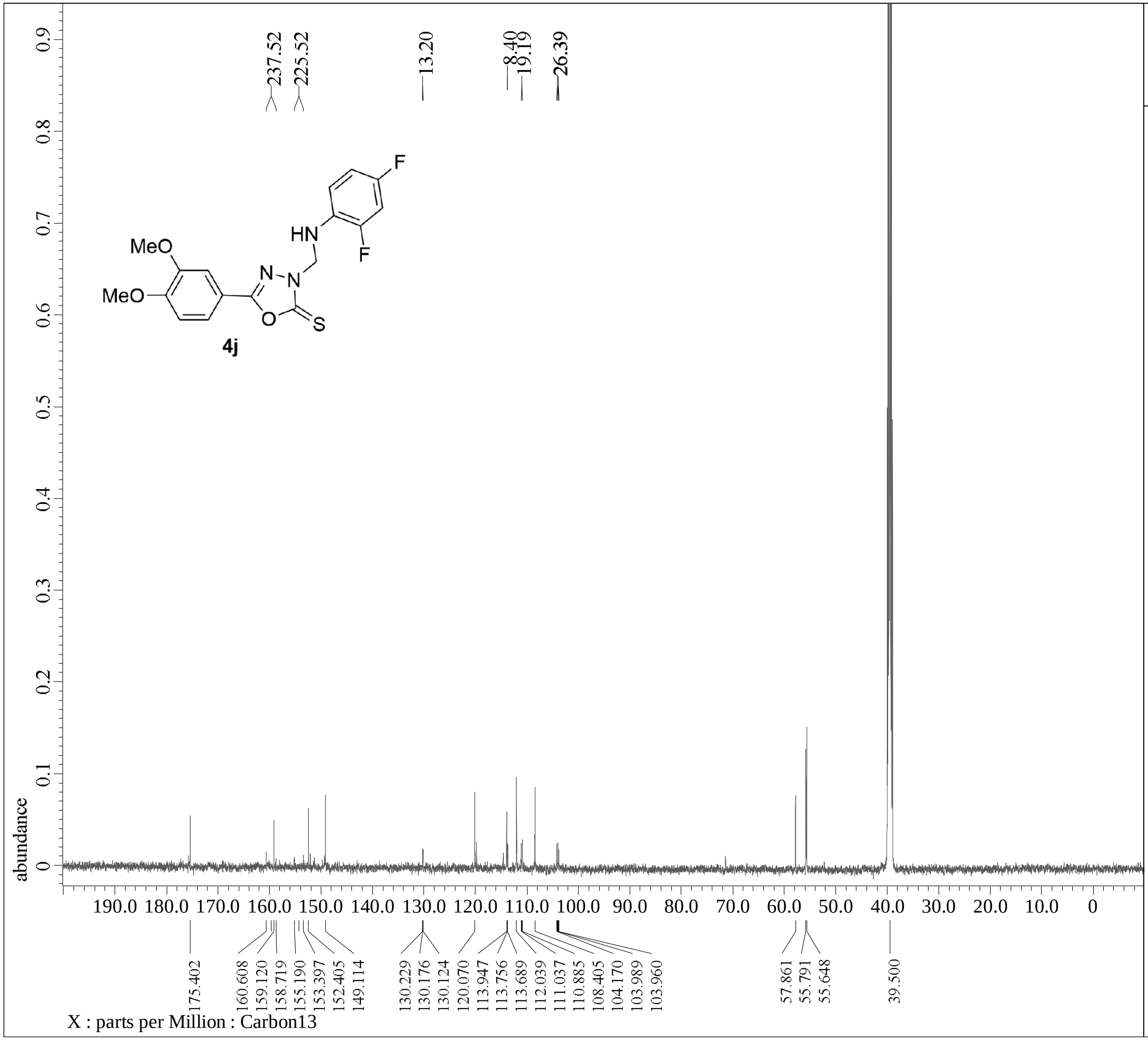


JEOL
Solutions for Innovation

```
Filename          = Prof.DR Ali Elemam_DM_15_c
Author            = delta
Experiment        = carbon.jxp
Sample_Id         = Prof.DR Ali Elemam_DM_15
Solvent           = DMSO-D6
Creation_Time     = 26-JAN-2021 01:54:58
Revision_Time     =  5-FEB-2021 05:09:19
Current_Time      =  5-FEB-2021 05:10:08

Comment           = single pulse decoupled gat
Data_Format       = 1D COMPLEX
Dim_Size          = 26214
Dim_Title         = Carbon13
Dim_Units         = [ppm]
Dimensions        = X
Site              = JNM-ECA500II
Spectrometer      = DELTA2_NMR

Field_Strength    = 11.7473579[T] (500[MHz])
X_Acq_Duration    = 0.83361792[s]
X_Domain          = 13C
X_Freq            = 125.76529768[MHz]
X_Offset          = 100[ppm]
X_Points          = 32768
X_Prescans        = 4
X_Resolution      = 1.19959034[Hz]
X_Sweep           = 39.3081761[kHz]
X_Sweep_Clipped   = 31.44654088[kHz]
Irr_Domain        = Proton
Irr_Freq          = 500.15991521[MHz]
Irr_Offset        = 5.0[ppm]
Clipped           = FALSE
Scans             = 1024
Total_Scans       = 1024

Relaxation_Delay  = 2[s]
Recvr_Gain        = 58
Temp_Get          = 20.1[dC]
X_90_Width        = 32.3[us]
X_Acq_Time        = 0.83361792[s]
X_Angle           = 30[deg]
X_Atn             = 10[dB]
X_Pulse           = 10.76666667[us]
Irr_Atn_Dec       = 17.693[dB]
Irr_Atn_Noe       = 17.693[dB]
Irr_Noise         = WALTZ
Irr_Pwidth        = 92[us]
Decoupling        = TRUE
Initial_Wait      = 1[s]
Noe               = TRUE
Noe_Time          = 2[s]
Repetition_Time   = 2.83361792[s]
```

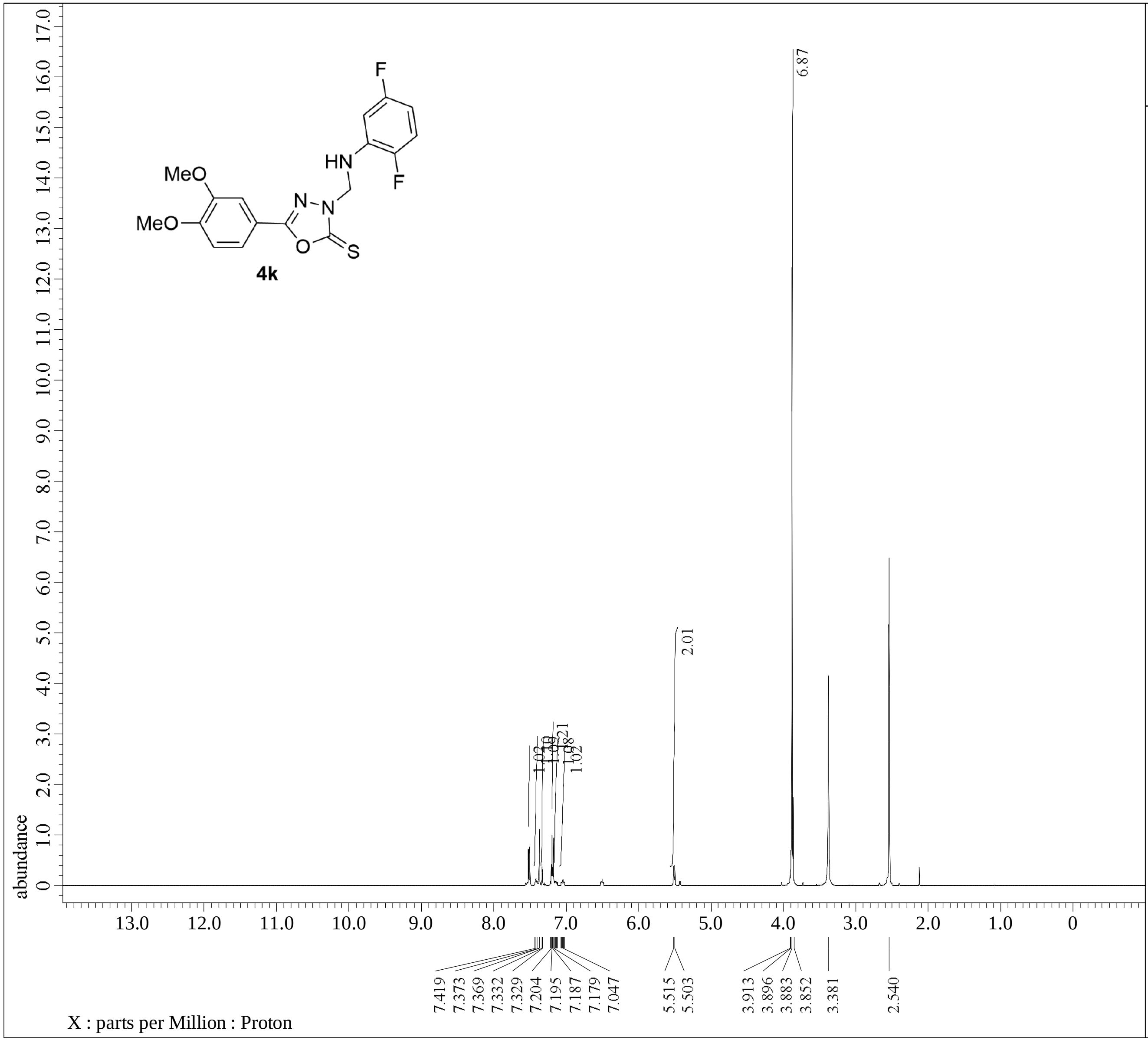


| | | |
|---|---|---|
| Filename | = | Prof.DR Ali Elemam_DM_16_p |
| Author | = | delta |
| Experiment | = | proton.jxp |
| Sample_Id | = | Prof.DR Ali Elemam_DM_16 |
| Solvent | = | DMSO-D6 |
| Creation_Time | = | 30-DEC-2020 13:10:51 |
| Revision_Time | = | 6-FEB-2021 10:36:20 |
| Current_Time | = | 6-FEB-2021 10:37:14 |
| Comment | = | single_pulse |
| Data_Format | = | 1D COMPLEX |
| Dim_Size | = | 13107 |
| Dim_Title | = | Proton |
| Dim_Units | = | [ppm] |
| Dimensions | = | X |
| Site | = | JNM-ECA500II |
| Spectrometer | = | DELTA2_NMR |
| Field_Strength | = | 11.7473579[T] (500[MHz]) |
| X_Acq_Duration | = | 1.24780544[s] |
| X_Domain | = | 1H |
| X_Freq | = | 500.15991521[MHz] |
| X_Offset | = | 7.0[ppm] |
| X_Points | = | 16384 |
| X_Prescans | = | 1 |
| X_Resolution | = | 0.80140699[Hz] |
| X_Sweep | = | 13.1302521[kHz] |
| X_Sweep_Clipped | = | 10.50420168[kHz] |
| Irr_Domain | = | Proton |
| Irr_Freq | = | 500.15991521[MHz] |
| Irr_Offset | = | 5.0[ppm] |
| Tri_Domain | = | Proton |
| Tri_Freq | = | 500.15991521[MHz] |
| Tri_Offset | = | 5.0[ppm] |
| Clipped | = | FALSE |
| Scans | = | 40 |
| Total_Scans | = | 40 |
| Relaxation_Delay | = | 5[s] |
| Recvr_Gain | = | 50 |
| Temp_Get | = | 18.8[dC] |
| X_90_Width | = | 16[us] |
| X_Acq_Time | = | 1.24780544[s] |
| X_Angle | = | 45[deg] |
| X_Atn | = | 2.5[dB] |
| X_Pulse | = | 8[us] |
| Irr_Mode | = | Off |
| Tri_Mode | = | Off |
| Dante_Presat | = | FALSE |
| Initial_Wait | = | 1[s] |
| Repetition_Time | = | 6.24780544[s] |

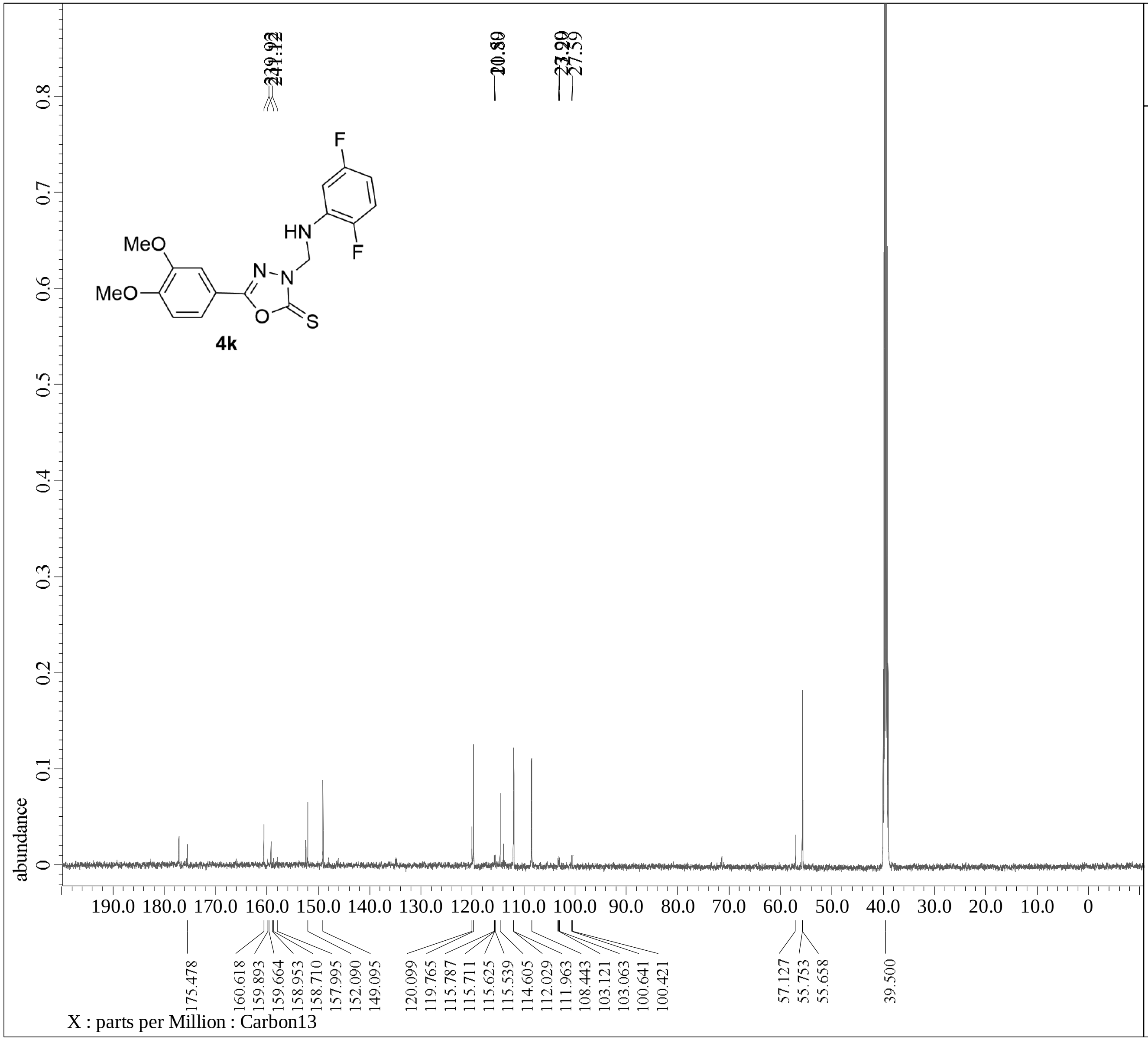


JEOL
Solutions for Innovation

```
Filename          = Prof.DR Ali Elemam_DM_16_c
Author            = delta
Experiment        = carbon.jxp
Sample_Id         = Prof.DR Ali Elemam_DM_16
Solvent           = DMSO-D6
Creation_Time     = 26-JAN-2021 02:51:44
Revision_Time     =  5-FEB-2021 05:52:07
Current_Time      =  5-FEB-2021 05:53:46

Comment           = single pulse decoupled gat
Data_Format       = 1D COMPLEX
Dim_Size          = 26214
Dim_Title         = Carbon13
Dim_Units         = [ppm]
Dimensions        = X
Site              = JNM-ECA500II
Spectrometer      = DELTA2_NMR

Field_Strength    = 11.7473579[T] (500[MHz])
X_Acq_Duration    = 0.83361792[s]
X_Domain          = 13C
X_Freq            = 125.76529768[MHz]
X_Offset          = 100[ppm]
X_Points          = 32768
X_Prescans        = 4
X_Resolution      = 1.19959034[Hz]
X_Sweep           = 39.3081761[kHz]
X_Sweep_Clipped   = 31.44654088[kHz]
Irr_Domain        = Proton
Irr_Freq          = 500.15991521[MHz]
Irr_Offset        = 5.0[ppm]
Clipped           = FALSE
Scans             = 1024
Total_Scans       = 1024

Relaxation_Delay  = 2[s]
Recvr_Gain        = 60
Temp_Get          = 20[dC]
X_90_Width        = 32.3[us]
X_Acq_Time        = 0.83361792[s]
X_Angle           = 30[deg]
X_Atn             = 10[dB]
X_Pulse           = 10.76666667[us]
Irr_Atn_Dec       = 17.693[dB]
Irr_Atn_Noe       = 17.693[dB]
Irr_Noise         = WALTZ
Irr_Pwidth        = 92[us]
Decoupling        = TRUE
Initial_Wait      = 1[s]
Noe               = TRUE
Noe_Time          = 2[s]
Repetition_Time   = 2.83361792[s]
```

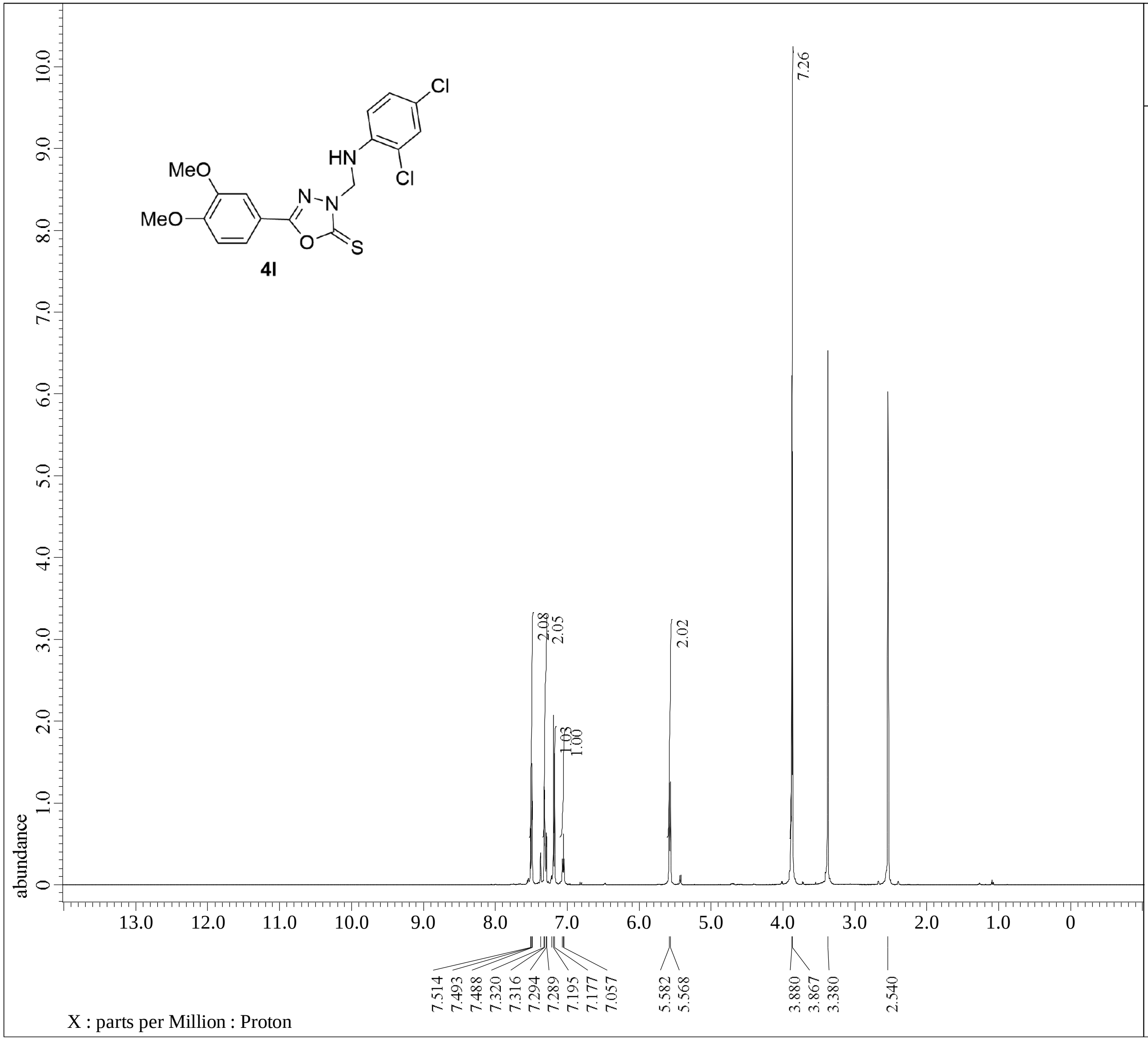


```
Filename          = Prof.DR Ali Elemam_DM_14_p
Author            = delta
Experiment        = proton.jxp
Sample_Id         = Prof.DR Ali Elemam_DM_14
Solvent           = DMSO-D6
Creation_Time     = 30-DEC-2020 14:40:18
Revision_Time     =  6-FEB-2021 10:07:38
Current_Time      =  6-FEB-2021 10:08:05

Comment           = single_pulse
Data_Format       = 1D COMPLEX
Dim_Size          = 13107
Dim_Title         = Proton
Dim_Units         = [ppm]
Dimensions        = X
Site              = JNM-ECA500II
Spectrometer      = DELTA2_NMR

Field_Strength    = 11.7473579[T] (500[MHz])
X_Acq_Duration    = 1.24780544[s]
X_Domain          = 1H
X_Freq            = 500.15991521[MHz]
X_Offset          = 7.0[ppm]
X_Points          = 16384
X_Prescans        = 1
X_Resolution      = 0.80140699[Hz]
X_Sweep           = 13.1302521[kHz]
X_Sweep_Clipped   = 10.50420168[kHz]
Irr_Domain        = Proton
Irr_Freq          = 500.15991521[MHz]
Irr_Offset        = 5.0[ppm]
Tri_Domain        = Proton
Tri_Freq          = 500.15991521[MHz]
Tri_Offset        = 5.0[ppm]
Clipped           = FALSE
Scans             = 40
Total_Scans       = 40

Relaxation_Delay  = 5[s]
Recvr_Gain        = 50
Temp_Get          = 18.8[dC]
X_90_Width        = 16[us]
X_Acq_Time        = 1.24780544[s]
X_Angle           = 45[deg]
X_Atn             = 2.5[dB]
X_Pulse           = 8[us]
Irr_Mode          = Off
Tri_Mode          = Off
Dante_Presat      = FALSE
Initial_Wait      = 1[s]
Repetition_Time   = 6.24780544[s]
```

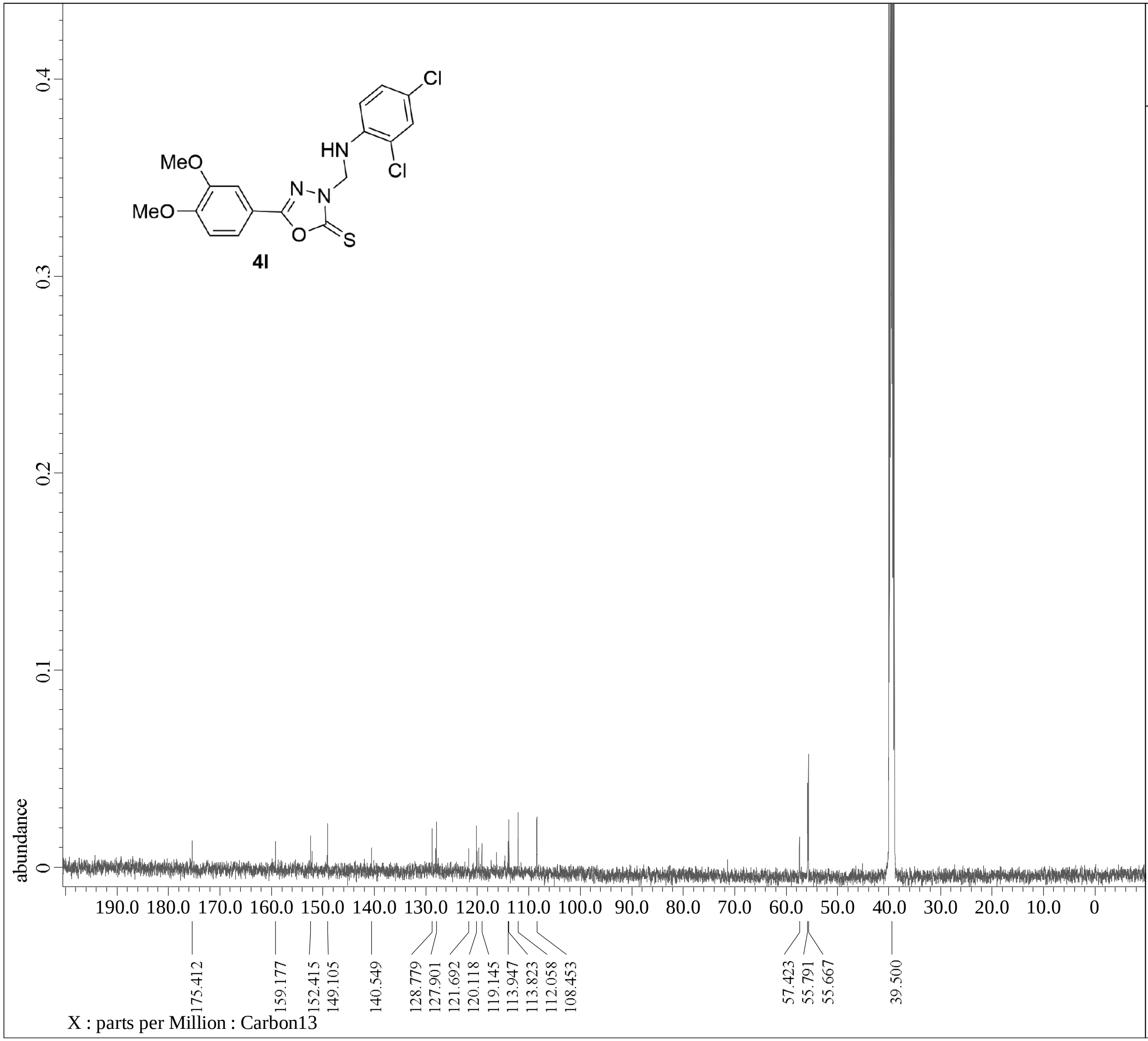


JEOL
Solutions for Innovation

| | |
|---|---|
| Filename | = Prof.DR Ali Elemam_DM_14_c |
| Author | = delta |
| Experiment | = carbon.jxp |
| Sample_Id | = Prof.DR Ali Elemam_DM_14 |
| Solvent | = DMSO-D6 |
| Creation_Time | = 3-FEB-2021 22:18:44 |
| Revision_Time | = 5-FEB-2021 04:46:58 |
| Current_Time | = 5-FEB-2021 04:48:17 |
| Comment | = single pulse decoupled gat |
| Data_Format | = 1D COMPLEX |
| Dim_Size | = 26214 |
| Dim_Title | = Carbon13 |
| Dim_Units | = [ppm] |
| Dimensions | = X |
| Site | = JNM-ECA500II |
| Spectrometer | = DELTA2_NMR |
| Field_Strength | = 11.7473579[T] (500[MHz]) |
| X_Acq_Duration | = 0.83361792[s] |
| X_Domain | = 13C |
| X_Freq | = 125.76529768[MHz] |
| X_Offset | = 100[ppm] |
| X_Points | = 32768 |
| X_Prescans | = 4 |
| X_Resolution | = 1.19959034[Hz] |
| X_Sweep | = 39.3081761[kHz] |
| X_Sweep_Clipped | = 31.44654088[kHz] |
| Irr_Domain | = Proton |
| Irr_Freq | = 500.15991521[MHz] |
| Irr_Offset | = 5.0[ppm] |
| Clipped | = FALSE |
| Scans | = 1850 |
| Total_Scans | = 1850 |
| Relaxation_Delay | = 2[s] |
| Recvr_Gain | = 58 |
| Temp_Get | = 19.8[dC] |
| X_90_Width | = 32.3[us] |
| X_Acq_Time | = 0.83361792[s] |
| X_Angle | = 30[deg] |
| X_Atn | = 10[dB] |
| X_Pulse | = 10.76666667[us] |
| Irr_Atn_Dec | = 17.693[dB] |
| Irr_Atn_Noe | = 17.693[dB] |
| Irr_Noise | = WALTZ |
| Irr_Pwidth | = 92[us] |
| Decoupling | = TRUE |
| Initial_Wait | = 1[s] |
| Noe | = TRUE |
| Noe_Time | = 2[s] |
| Repetition_Time | = 2.83361792[s] |

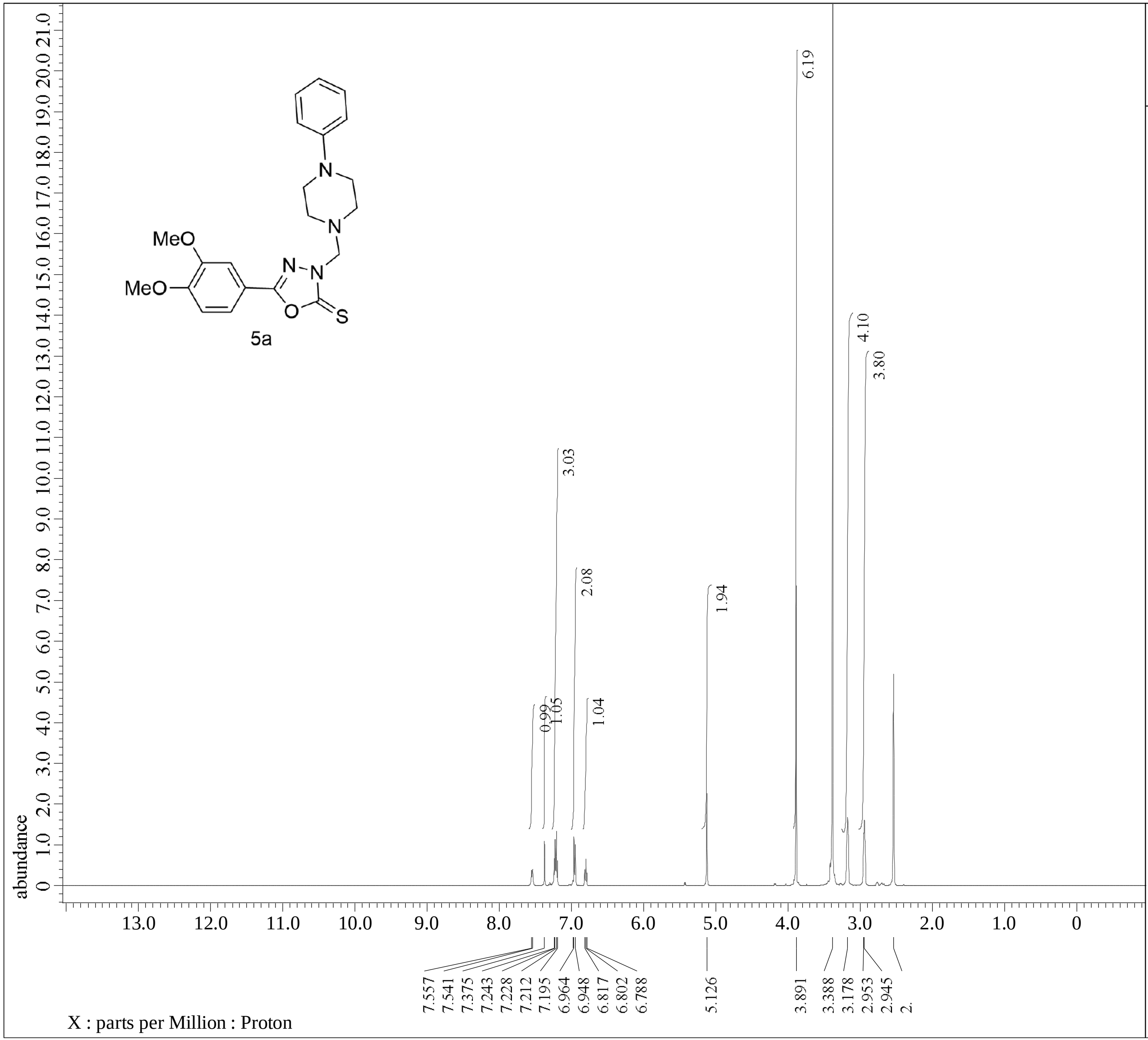


```
Filename          = Prof.DR Ali Elemam_DM_1_pr
Author            = delta
Experiment        = proton.jxp
Sample_Id         = Prof.DR Ali Elemam_DM_1
Solvent           = DMSO-D6
Creation_Time     = 14-DEC-2020 11:41:11
Revision_Time     =  5-FEB-2021 23:12:44
Current_Time      =  5-FEB-2021 23:13:51

Comment           = single_pulse
Data_Format       = 1D COMPLEX
Dim_Size          = 13107
Dim_Title         = Proton
Dim_Units         = [ppm]
Dimensions        = X
Site              = JNM-ECA500II
Spectrometer      = DELTA2_NMR

Field_Strength    = 11.7473579[T] (500[MHz])
X_Acq_Duration    = 1.24780544[s]
X_Domain          = 1H
X_Freq            = 500.15991521[MHz]
X_Offset          = 7.0[ppm]
X_Points          = 16384
X_Prescans        = 1
X_Resolution      = 0.80140699[Hz]
X_Sweep           = 13.1302521[kHz]
X_Sweep_Clipped   = 10.50420168[kHz]
Irr_Domain        = Proton
Irr_Freq          = 500.15991521[MHz]
Irr_Offset        = 5.0[ppm]
Tri_Domain        = Proton
Tri_Freq          = 500.15991521[MHz]
Tri_Offset        = 5.0[ppm]
Clipped           = FALSE
Scans             = 40
Total_Scans       = 40

Relaxation_Delay  = 5[s]
Recvr_Gain        = 50
Temp_Get          = 18.9[dC]
X_90_Width        = 16[us]
X_Acq_Time        = 1.24780544[s]
X_Angle           = 45[deg]
X_Atn             = 2.5[dB]
X_Pulse           = 8[us]
Irr_Mode          = Off
Tri_Mode          = Off
Dante_Presat      = FALSE
Initial_Wait      = 1[s]
Repetition_Time   = 6.24780544[s]
```

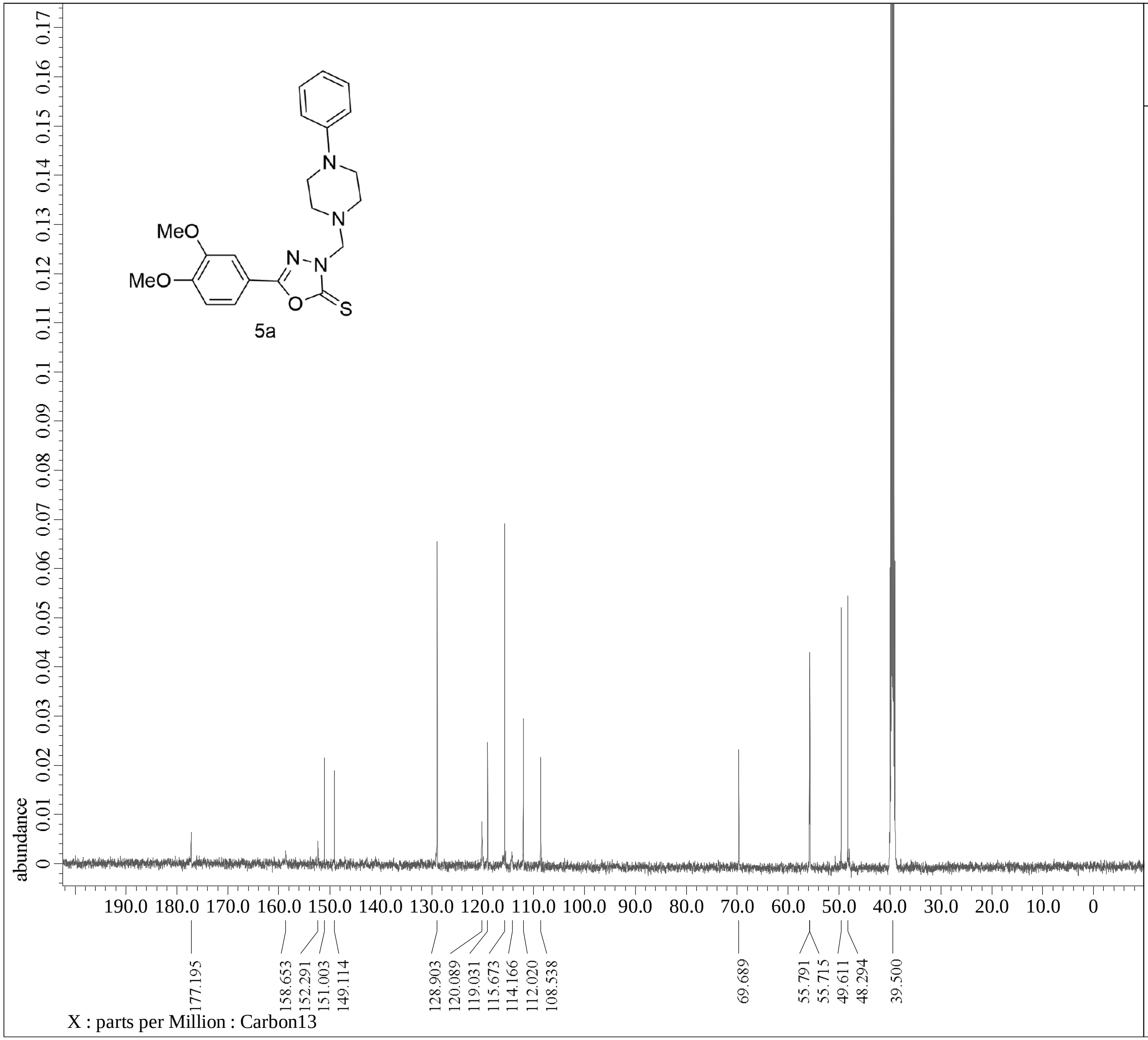


```
Filename          = Prof.DR Ali Elemam_DM_1_ca
Author            = delta
Experiment        = carbon.jxp
Sample_Id         = Prof.DR Ali Elemam_DM_1
Solvent           = DMSO-D6
Creation_Time     = 21-DEC-2020 19:28:08
Revision_Time     =  5-FEB-2021 08:35:11
Current_Time      =  5-FEB-2021 08:36:32

Comment           = single pulse decoupled gat
Data_Format       = 1D COMPLEX
Dim_Size          = 26214
Dim_Title         = Carbon13
Dim_Units         = [ppm]
Dimensions        = X
Site              = JNM-ECA500II
Spectrometer      = DELTA2_NMR

Field_Strength    = 11.7473579[T] (500[MHz])
X_Acq_Duration    = 0.83361792[s]
X_Domain          = 13C
X_Freq            = 125.76529768[MHz]
X_Offset          = 100[ppm]
X_Points          = 32768
X_Prescans        = 4
X_Resolution      = 1.19959034[Hz]
X_Sweep           = 39.3081761[kHz]
X_Sweep_Clipped   = 31.44654088[kHz]
Irr_Domain        = Proton
Irr_Freq          = 500.15991521[MHz]
Irr_Offset        = 5.0[ppm]
Clipped           = FALSE
Scans             = 1500
Total_Scans       = 1500

Relaxation_Delay  = 2[s]
Recvr_Gain        = 50
Temp_Get          = 20.3[dC]
X_90_Width        = 32.3[us]
X_Acq_Time        = 0.83361792[s]
X_Angle           = 30[deg]
X_Atn             = 10[dB]
X_Pulse           = 10.76666667[us]
Irr_Atn_Dec       = 17.693[dB]
Irr_Atn_Noe       = 17.693[dB]
Irr_Noise         = WALTZ
Irr_Pwidth        = 92[us]
Decoupling        = TRUE
Initial_Wait      = 1[s]
Noe               = TRUE
Noe_Time          = 2[s]
Repetition_Time   = 2.83361792[s]
```

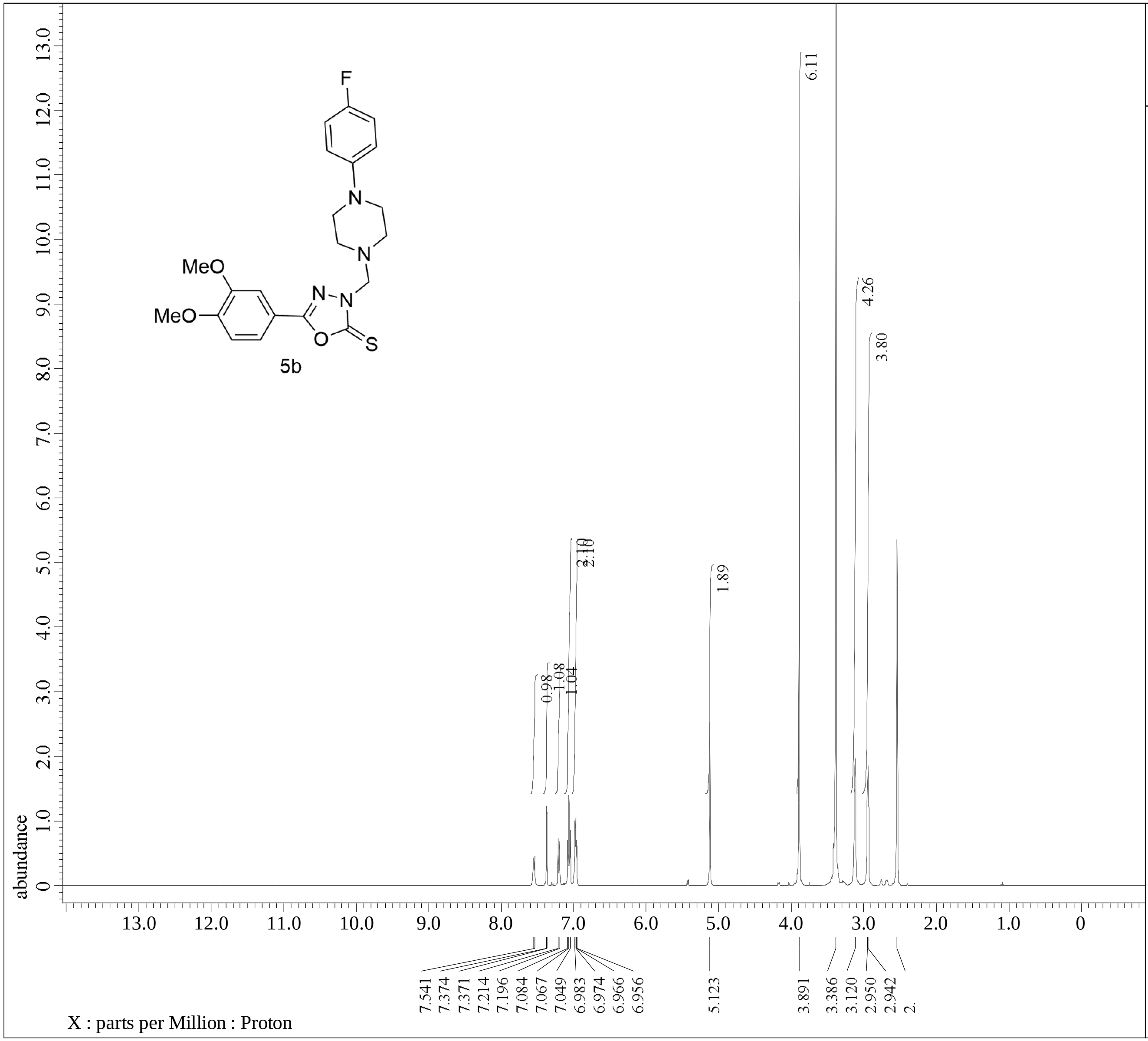


```
Filename          = Prof.DR Ali Elemam_DM_2_pr
Author            = delta
Experiment        = proton.jxp
Sample_Id         = Prof.DR Ali Elemam_DM_2
Solvent           = DMSO-D6
Creation_Time     = 14-DEC-2020 11:50:57
Revision_Time     =  5-FEB-2021 23:19:09
Current_Time      =  5-FEB-2021 23:19:16

Comment           = single_pulse
Data_Format       = 1D COMPLEX
Dim_Size          = 13107
Dim_Title         = Proton
Dim_Units         = [ppm]
Dimensions        = X
Site              = JNM-ECA500II
Spectrometer      = DELTA2_NMR

Field_Strength    = 11.7473579[T] (500[MHz])
X_Acq_Duration    = 1.24780544[s]
X_Domain          = 1H
X_Freq            = 500.15991521[MHz]
X_Offset          = 7.0[ppm]
X_Points          = 16384
X_Prescans        = 1
X_Resolution      = 0.80140699[Hz]
X_Sweep           = 13.1302521[kHz]
X_Sweep_Clipped   = 10.50420168[kHz]
Irr_Domain        = Proton
Irr_Freq          = 500.15991521[MHz]
Irr_Offset        = 5.0[ppm]
Tri_Domain        = Proton
Tri_Freq          = 500.15991521[MHz]
Tri_Offset        = 5.0[ppm]
Clipped           = FALSE
Scans             = 40
Total_Scans       = 40

Relaxation_Delay  = 5[s]
Recvr_Gain        = 50
Temp_Get          = 19[dC]
X_90_Width        = 16[us]
X_Acq_Time        = 1.24780544[s]
X_Angle           = 45[deg]
X_Atn             = 2.5[dB]
X_Pulse           = 8[us]
Irr_Mode          = Off
Tri_Mode          = Off
Dante_Presat      = FALSE
Initial_Wait      = 1[s]
Repetition_Time   = 6.24780544[s]
```

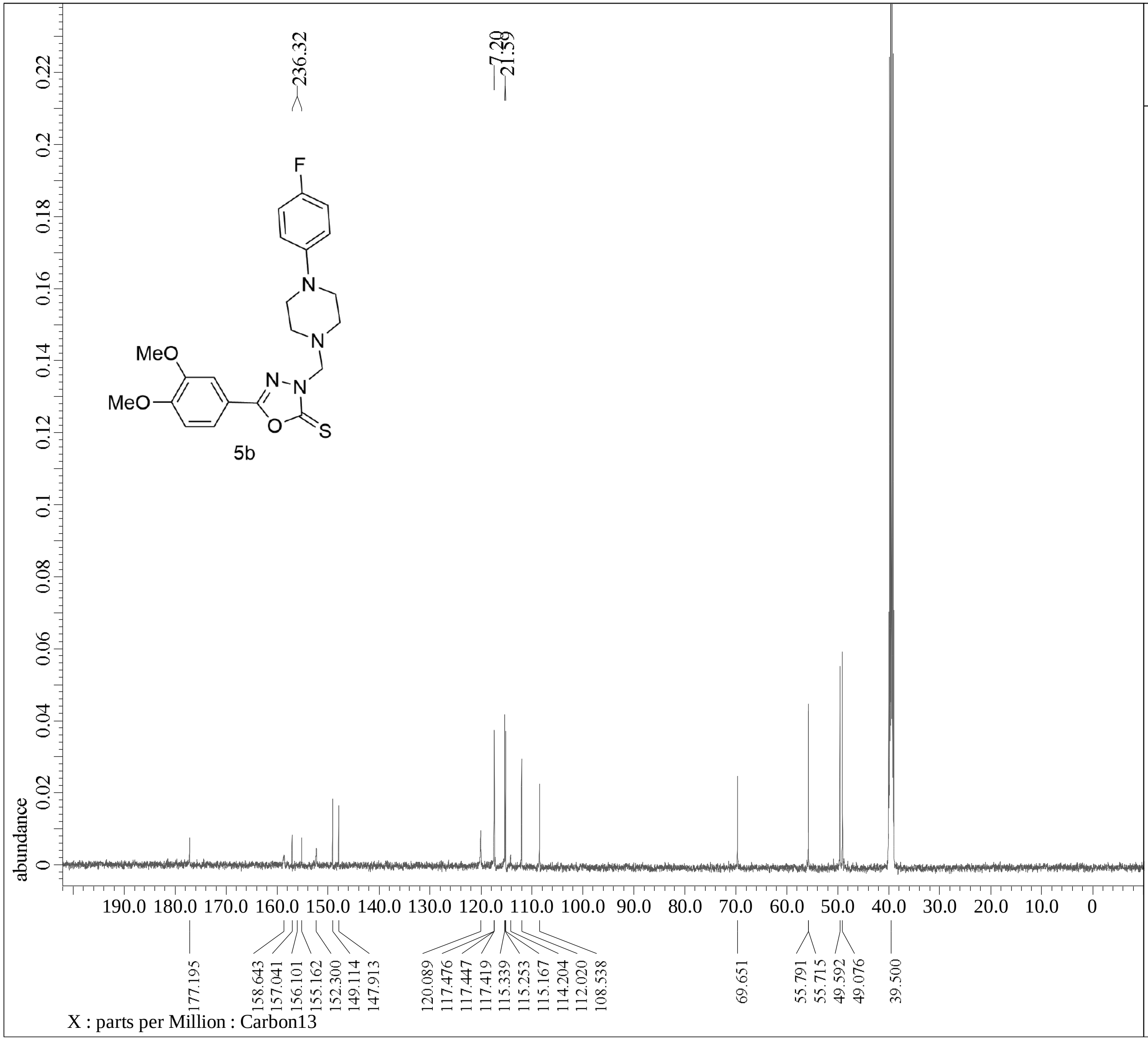


Filename = Prof.DR Ali Elemam_DM_2_ca
Author = delta
Experiment = carbon.jxp
Sample_Id = Prof.DR Ali Elemam_DM_2
Solvent = DMSO-D6
Creation_Time = 21-DEC-2020 20:44:41
Revision_Time = 5-FEB-2021 08:45:38
Current_Time = 5-FEB-2021 08:49:17

Comment = single pulse decoupled gat
Data_Format = 1D COMPLEX
Dim_Size = 26214
Dim_Title = Carbon13
Dim_Units = [ppm]
Dimensions = X
Site = JNM-ECA500II
Spectrometer = DELTA2_NMR

Field_Strength = 11.7473579[T] (500[MHz])
X_Acq_Duration = 0.83361792[s]
X_Domain = 13C
X_Freq = 125.76529768[MHz]
X_Offset = 100[ppm]
X_Points = 32768
X_Prescans = 4
X_Resolution = 1.19959034[Hz]
X_Sweep = 39.3081761[kHz]
X_Sweep_Clipped = 31.44654088[kHz]
Irr_Domain = Proton
Irr_Freq = 500.15991521[MHz]
Irr_Offset = 5.0[ppm]
Clipped = FALSE
Scans = 1500
Total_Scans = 1500

Relaxation_Delay = 2[s]
Recvr_Gain = 50
Temp_Get = 19.9[dC]
X_90_Width = 32.3[us]
X_Acq_Time = 0.83361792[s]
X_Angle = 30[deg]
X_Atn = 10[dB]
X_Pulse = 10.76666667[us]
Irr_Atn_Dec = 17.693[dB]
Irr_Atn_Noe = 17.693[dB]
Irr_Noise = WALTZ
Irr_Pwidth = 92[us]
Decoupling = TRUE
Initial_Wait = 1[s]
Noe = TRUE
Noe_Time = 2[s]
Repetition_Time = 2.83361792[s]

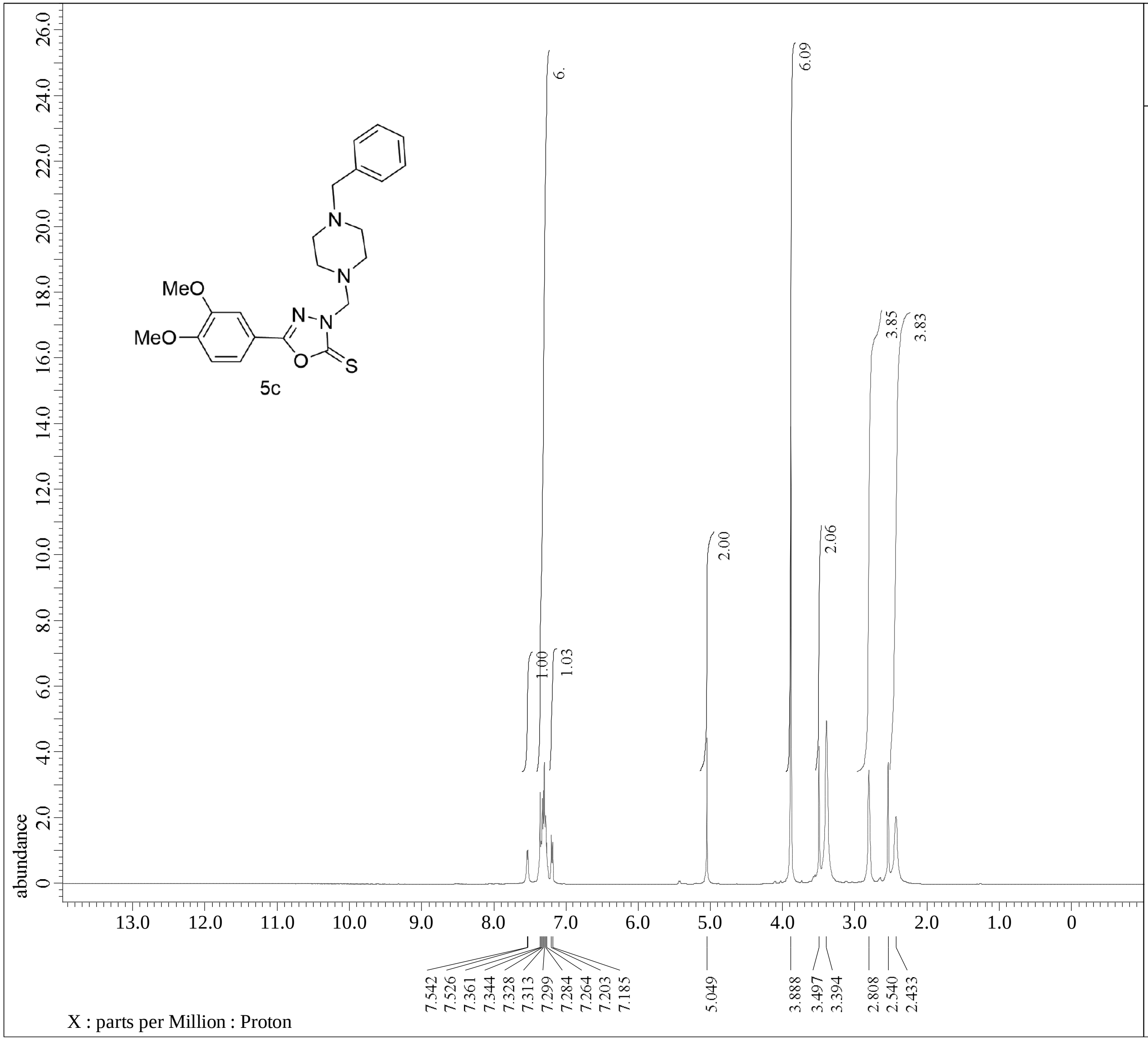


Filename = Prof.DR Ali Elemam_DM_3_pr
Author = delta
Experiment = proton.jxp
Sample_Id = Prof.DR Ali Elemam_DM_3
Solvent = DMSO-D6
Creation_Time = 14-DEC-2020 12:02:59
Revision_Time = 5-FEB-2021 23:22:48
Current_Time = 5-FEB-2021 23:23:09

Comment = single_pulse
Data_Format = 1D COMPLEX
Dim_Size = 13107
Dim_Title = Proton
Dim_Units = [ppm]
Dimensions = X
Site = JNM-ECA500II
Spectrometer = DELTA2_NMR

Field_Strength = 11.7473579[T] (500[MHz])
X_Acq_Duration = 1.24780544[s]
X_Domain = 1H
X_Freq = 500.15991521[MHz]
X_Offset = 7.0[ppm]
X_Points = 16384
X_Prescans = 1
X_Resolution = 0.80140699[Hz]
X_Sweep = 13.1302521[kHz]
X_Sweep_Clipped = 10.50420168[kHz]
Irr_Domain = Proton
Irr_Freq = 500.15991521[MHz]
Irr_Offset = 5.0[ppm]
Tri_Domain = Proton
Tri_Freq = 500.15991521[MHz]
Tri_Offset = 5.0[ppm]
Clipped = TRUE
Scans = 40
Total_Scans = 40

Relaxation_Delay = 5[s]
Recvr_Gain = 50
Temp_Get = 18.7[dC]
X_90_Width = 16[us]
X_Acq_Time = 1.24780544[s]
X_Angle = 45[deg]
X_Atn = 2.5[dB]
X_Pulse = 8[us]
Irr_Mode = Off
Tri_Mode = Off
Dante_Presat = FALSE
Initial_Wait = 1[s]
Repetition_Time = 6.24780544[s]

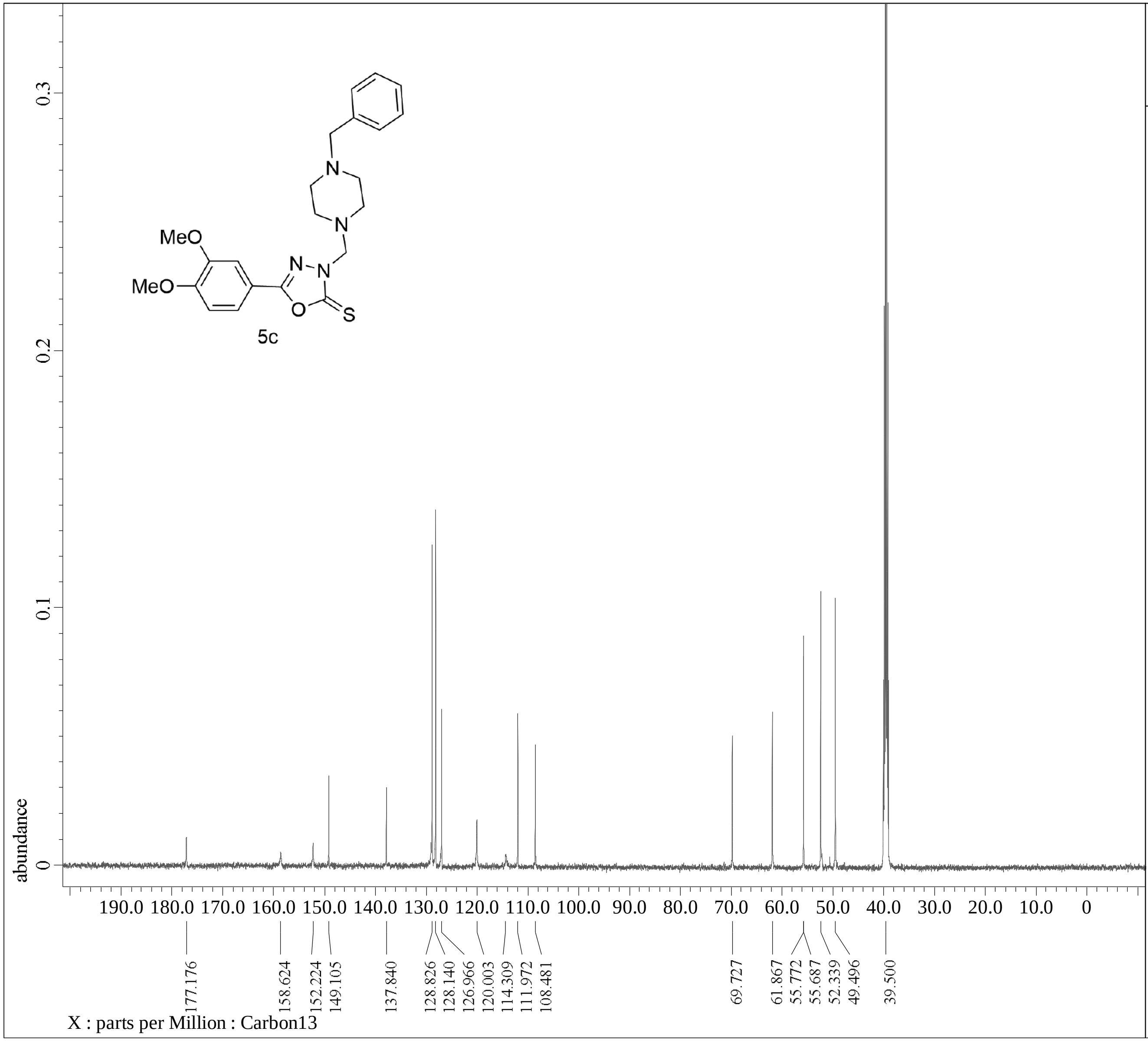


| | | |
|---|---|---|
| Filename | = | Prof.DR Ali Elemam_DM_3_ca |
| Author | = | delta |
| Experiment | = | carbon.jxp |
| Sample_Id | = | Prof.DR Ali Elemam_DM_3 |
| Solvent | = | DMSO-D6 |
| Creation_Time | = | 21-DEC-2020 22:01:52 |
| Revision_Time | = | 5-FEB-2021 08:52:07 |
| Current_Time | = | 5-FEB-2021 08:53:24 |
| Comment | = | single pulse decoupled gat |
| Data_Format | = | 1D COMPLEX |
| Dim_Size | = | 26214 |
| Dim_Title | = | Carbon13 |
| Dim_Units | = | [ppm] |
| Dimensions | = | X |
| Site | = | JNM-ECA500II |
| Spectrometer | = | DELTA2_NMR |
| Field_Strength | = | 11.7473579[T] (500[MHz]) |
| X_Acq_Duration | = | 0.83361792[s] |
| X_Domain | = | 13C |
| X_Freq | = | 125.76529768[MHz] |
| X_Offset | = | 100[ppm] |
| X_Points | = | 32768 |
| X_Prescans | = | 4 |
| X_Resolution | = | 1.19959034[Hz] |
| X_Sweep | = | 39.3081761[kHz] |
| X_Sweep_Clipped | = | 31.44654088[kHz] |
| Irr_Domain | = | Proton |
| Irr_Freq | = | 500.15991521[MHz] |
| Irr_Offset | = | 5.0[ppm] |
| Clipped | = | FALSE |
| Scans | = | 1500 |
| Total_Scans | = | 1500 |
| Relaxation_Delay | = | 2[s] |
| Recvr_Gain | = | 50 |
| Temp_Get | = | 20.1[dC] |
| X_90_Width | = | 32.3[us] |
| X_Acq_Time | = | 0.83361792[s] |
| X_Angle | = | 30[deg] |
| X_Atn | = | 10[dB] |
| X_Pulse | = | 10.76666667[us] |
| Irr_Atn_Dec | = | 17.693[dB] |
| Irr_Atn_Noe | = | 17.693[dB] |
| Irr_Noise | = | WALTZ |
| Irr_Pwidth | = | 92[us] |
| Decoupling | = | TRUE |
| Initial_Wait | = | 1[s] |
| Noe | = | TRUE |
| Noe_Time | = | 2[s] |
| Repetition_Time | = | 2.83361792[s] |

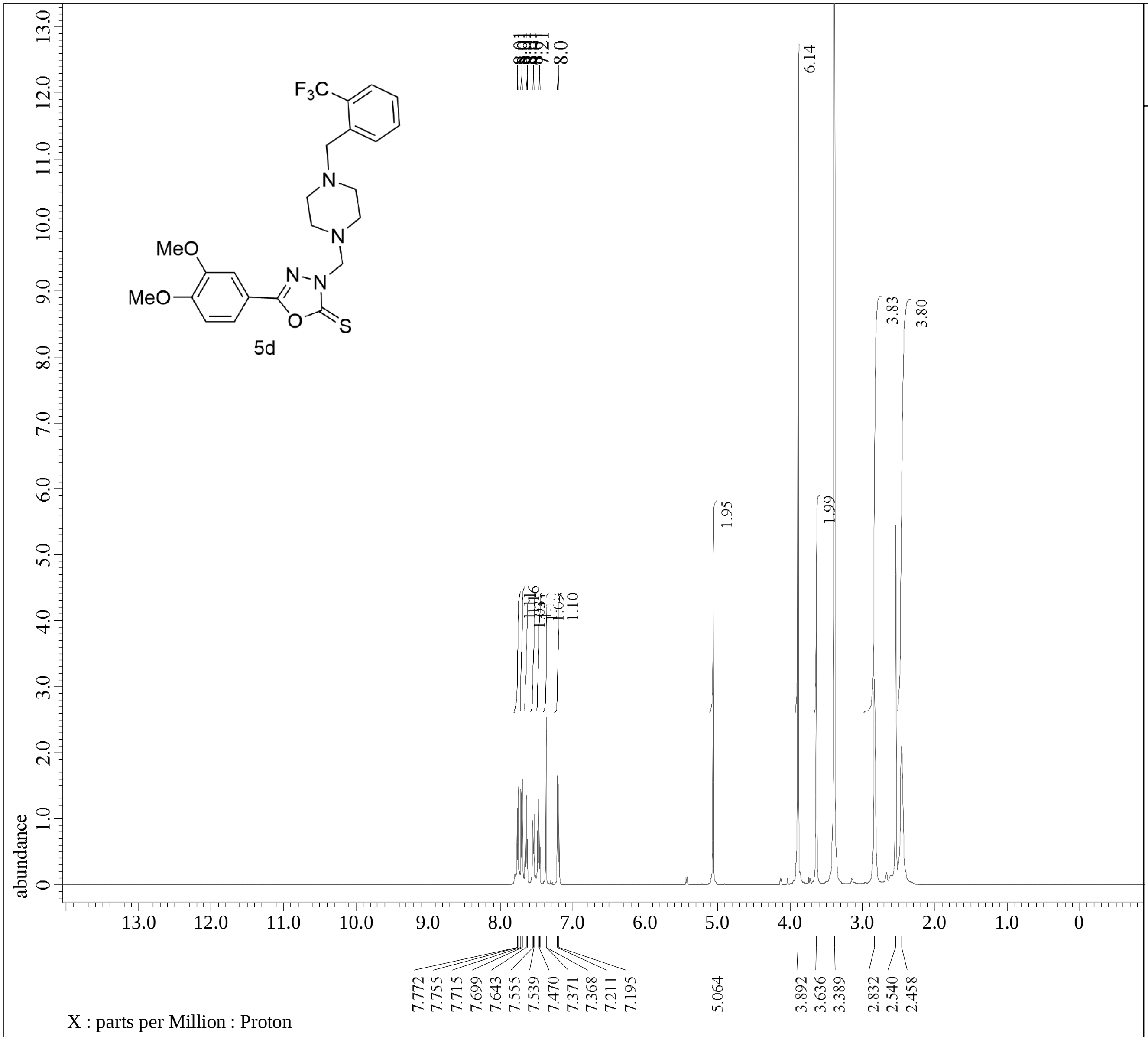


JEOL
Solutions for Innovation

```
Filename          = Prof.DR Ali Elemam_DM_4_pr
Author            = delta
Experiment        = proton.jxp
Sample_Id         = Prof.DR Ali Elemam_DM_4
Solvent           = DMSO-D6
Creation_Time     = 14-DEC-2020 12:13:29
Revision_Time     =  5-FEB-2021 23:27:13
Current_Time      =  5-FEB-2021 23:28:02

Comment           = single_pulse
Data_Format       = 1D COMPLEX
Dim_Size          = 13107
Dim_Title         = Proton
Dim_Units         = [ppm]
Dimensions        = X
Site              = JNM-ECA500II
Spectrometer      = DELTA2_NMR

Field_Strength    = 11.7473579[T] (500[MHz])
X_Acq_Duration    = 1.24780544[s]
X_Domain          = 1H
X_Freq            = 500.15991521[MHz]
X_Offset          = 7.0[ppm]
X_Points          = 16384
X_Prescans        = 1
X_Resolution      = 0.80140699[Hz]
X_Sweep           = 13.1302521[kHz]
X_Sweep_Clipped   = 10.50420168[kHz]
Irr_Domain        = Proton
Irr_Freq          = 500.15991521[MHz]
Irr_Offset        = 5.0[ppm]
Tri_Domain        = Proton
Tri_Freq          = 500.15991521[MHz]
Tri_Offset        = 5.0[ppm]
Clipped           = TRUE
Scans             = 40
Total_Scans       = 40

Relaxation_Delay  = 5[s]
Recvr_Gain        = 50
Temp_Get          = 18.8[dC]
X_90_Width        = 16[us]
X_Acq_Time        = 1.24780544[s]
X_Angle           = 45[deg]
X_Atn             = 2.5[dB]
X_Pulse           = 8[us]
Irr_Mode          = Off
Tri_Mode          = Off
Dante_Presat      = FALSE
Initial_Wait      = 1[s]
Repetition_Time   = 6.24780544[s]
```

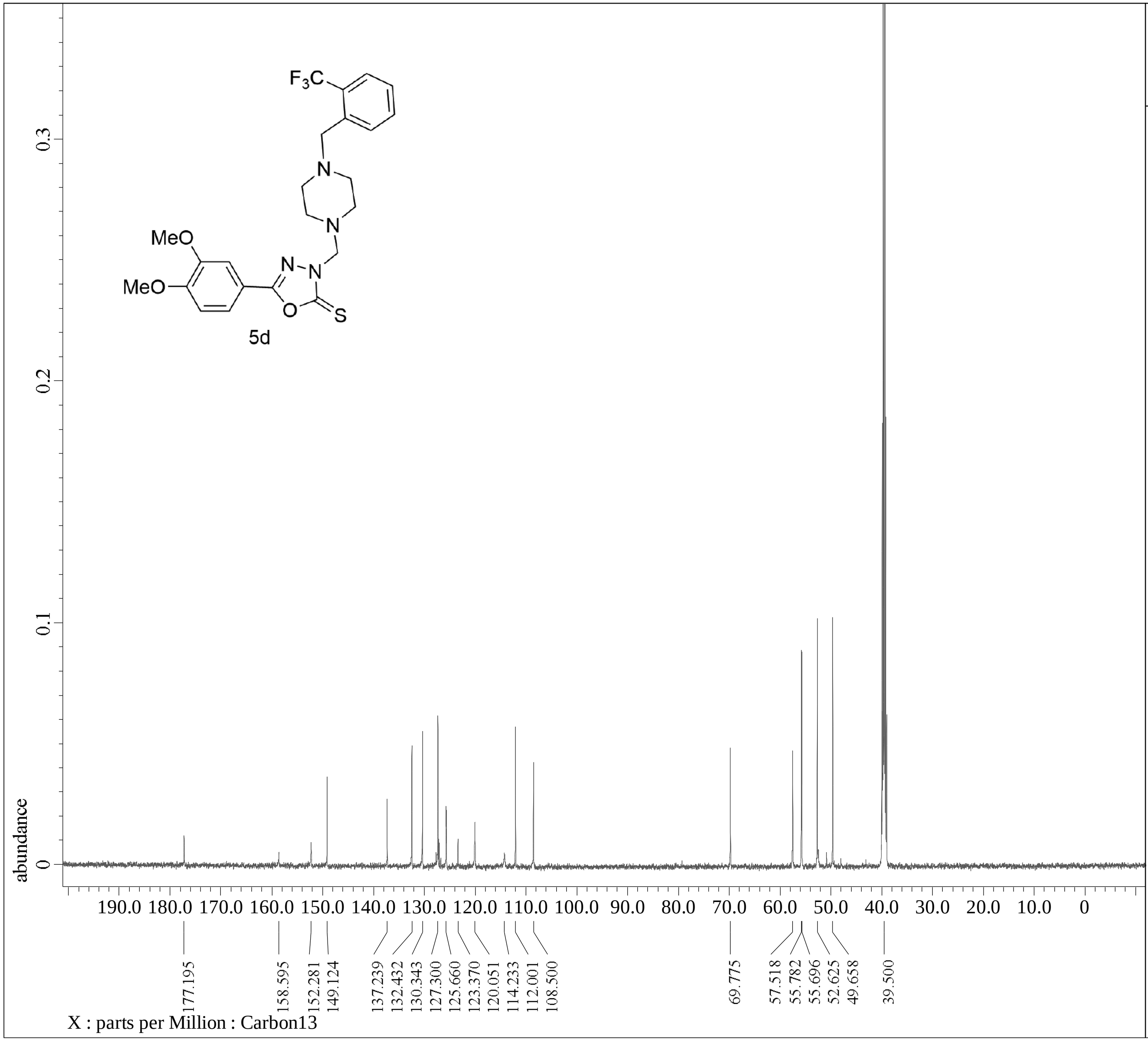


```
Filename          = Prof.DR Ali Elemam_DM_4_ca
Author            = delta
Experiment        = carbon.jxp
Sample_Id         = Prof.DR Ali Elemam_DM_4
Solvent           = DMSO-D6
Creation_Time     = 22-DEC-2020 08:58:10
Revision_Time     =  5-FEB-2021 09:06:07
Current_Time      =  5-FEB-2021 09:07:41

Comment           = single pulse decoupled gat
Data_Format       = 1D COMPLEX
Dim_Size          = 26214
Dim_Title         = Carbon13
Dim_Units         = [ppm]
Dimensions        = X
Site              = JNM-ECA500II
Spectrometer      = DELTA2_NMR

Field_Strength    = 11.7473579[T] (500[MHz])
X_Acq_Duration    = 0.83361792[s]
X_Domain          = 13C
X_Freq            = 125.76529768[MHz]
X_Offset          = 100[ppm]
X_Points          = 32768
X_Prescans        = 4
X_Resolution      = 1.19959034[Hz]
X_Sweep           = 39.3081761[kHz]
X_Sweep_Clipped   = 31.44654088[kHz]
Irr_Domain        = Proton
Irr_Freq          = 500.15991521[MHz]
Irr_Offset        = 5.0[ppm]
Clipped           = FALSE
Scans             = 1500
Total_Scans       = 1500

Relaxation_Delay  = 2[s]
Recvr_Gain        = 50
Temp_Get          = 18.9[dC]
X_90_Width        = 32.3[us]
X_Acq_Time        = 0.83361792[s]
X_Angle           = 30[deg]
X_Atn             = 10[dB]
X_Pulse           = 10.76666667[us]
Irr_Atn_Dec       = 17.693[dB]
Irr_Atn_Noe       = 17.693[dB]
Irr_Noise         = WALTZ
Irr_Pwidth        = 92[us]
Decoupling        = TRUE
Initial_Wait      = 1[s]
Noe               = TRUE
Noe_Time          = 2[s]
Repetition_Time   = 2.83361792[s]
```
